# The *map-1* Gene Family in Root-Knot Nematodes, *Meloidogyne* spp.: A Set of Taxonomically Restricted Genes Specific to Clonal Species

**DOI:** 10.1371/journal.pone.0038656

**Published:** 2012-06-18

**Authors:** Iva Tomalova, Cathy Iachia, Karine Mulet, Philippe Castagnone-Sereno

**Affiliations:** 1 French National Institute for Agriculture Research (INRA), Institut Sophia Agrobiotech, Sophia Antipolis, France; 2 University of Nice- Sophia Antipolis (UNSA), UMR Institut Sophia Agrobiotech, Sophia Antipolis, France; 3 Centre National se la Recherche Scientifique (CNRS), Institut Sophia Agrobiotech, Sophia Antipolis, France; Children’s Hospital Los Angeles, United States of America

## Abstract

Taxonomically restricted genes (TRGs), i.e., genes that are restricted to a limited subset of phylogenetically related organisms, may be important in adaptation. In parasitic organisms, TRG-encoded proteins are possible determinants of the specificity of host-parasite interactions. In the root-knot nematode (RKN) *Meloidogyne incognita*, the *map-1* gene family encodes expansin-like proteins that are secreted into plant tissues during parasitism, thought to act as effectors to promote successful root infection. MAP-1 proteins exhibit a modular architecture, with variable number and arrangement of 58 and 13-aa domains in their central part. Here, we address the evolutionary origins of this gene family using a combination of bioinformatics and molecular biology approaches. *Map-1* genes were solely identified in one single member of the phylum Nematoda, i.e., the genus *Meloidogyne*, and not detected in any other nematode, thus indicating that the *map-1* gene family is indeed a TRG family. A phylogenetic analysis of the distribution of *map-1* genes in RKNs further showed that these genes are specifically present in species that reproduce by mitotic parthenogenesis, with the exception of *M. floridensis*, and could not be detected in RKNs reproducing by either meiotic parthenogenesis or amphimixis. These results highlight the divergence between mitotic and meiotic RKN species as a critical transition in the evolutionary history of these parasites. Analysis of the sequence conservation and organization of repeated domains in *map-1* genes suggests that gene duplication(s) together with domain loss/duplication have contributed to the evolution of the *map-1* family, and that some strong selection mechanism may be acting upon these genes to maintain their functional role(s) in the specificity of the plant-RKN interactions.

## Introduction

The phylum Nematoda comprises over 25,000 described species, many of which are parasites of animals or plants [1]. Among them, root-knot nematodes (RKNs), *Meloidogyne* spp., are extremely polyphagous species able to infect the roots of almost all cultivated plants, being responsible for estimated losses of more than 80 billions Euros/year [2]. These nematodes are obligatory, sedentary endoparasites which have evolved an intimate interaction with their hosts, by inducing the redifferentiation of root cells into permanent feeding sites [3]. RKNs display very different modes of reproduction, from amphimixis to mitotic parthenogenesis. It is generally admitted that sexual reproduction is the ancestral state, and that parthenogenetic species evolved from amphimictic ancestors [4], [5], although a still running debate exists on whether the establishment of parthenogenesis in these nematodes is of ancient or recent origin [6], [7]. At a worldwide scale, the most agriculturally widespread and damaging species are by far the three closely related, mitotic parthenogens *M. incognita*, *M. javanica* and *M. arenaria.*

**Figure 1:**
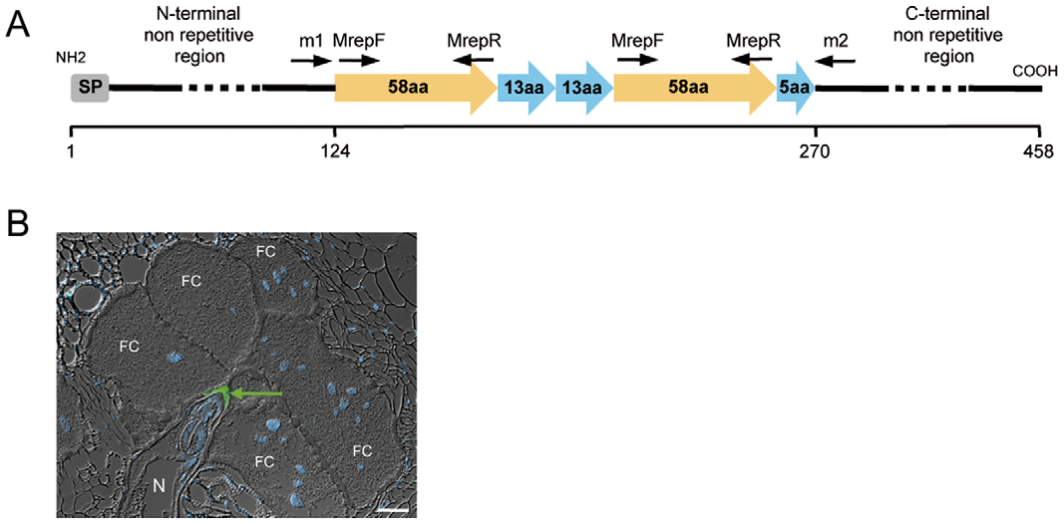
The MAP-1 protein in *Meloidogyne incognita.* (A) Schematic representation of the predicted MAP-1 protein. The black arrows indicate the location of the m1/m2 and MrepF/MrepR specific primer pairs used in this study (adapted from [8]). (B) *In planta* immunodetection of the MAP-1 protein (green arrow) secreted by the nematode (N) in the root tissues in the viscinity of the feeding cells (FC)(adapted from [11]).

In *M. incognita*, the MAP-1 protein was originally identified as a putative avirulence factor shown to be secreted by infective juveniles [8]. It was thus hypothesized that the protein could be involved in the early steps of recognition between the plant and the nematode. At the time of its discovery, *map-1* did not show any significant similarity in databases. However, a wide phylogenetic analysis recently positioned *M. incognita map-1* homologues into a cluster of genes encoding expansin-like proteins [9]. Since expansins are thought to be secreted by nematodes to loosen plant cell walls when invading their hosts [10], it reinforced the hypothesis of a role of MAP-1 during the early phase(s) of the plant-nematode interaction. However, further immunolocalisation studies demonstrated that the protein was also secreted *in planta* by both migratory and sedentary stages, suggesting an additional role of the protein in the initiation and/or maintenance of the feeding site [11], [12]. MAP-1 exhibited highly conserved repetitive motifs of 13 and 58 aa, respectively, in its internal part [8] (Fig. 1). Recently, it was shown that *map-1* belongs to a small gene family, and that variation in the number/arrangement of internal repeats in the genes was correlated with the (a)virulence of *M. incognita* near-isogenic lines [13]. These results clearly suggest that gene duplication, dynamic rearrangements in the repeats and gene loss are the main forces driving the evolution of the *map-1* gene family in this nematode.

In a previous study, the genomic distribution of *map-1*-related sequences was found restricted to the three closely related mitotic RKN species, i.e., *M. incognita*, *M. javanica* and *M. arenaria*, while no homologous sequence was detected in *M. hapla* and *M. fallax*, in more distantly related plant-parasitic species (i.e., *Globodera pallida*, *Xiphinema index*) and in the free-living species *Caenorhabditis elegans* [8]. From that point of view, members of the *map-1* gene family may be considered as ‘orphans’ or ‘taxonomically restricted genes’ (TRGs). The occurrence of such TRGs is thought to reflect important evolutionary processes, and their study could reveal the genotypic basis of exclusive ecological adaptations and the generation of lineage specific traits [14]–[16].

Recently, the genome sequences of several nematode species, including the RKNs *M. incognita* and *M. hapla* [17], [18], became available and provide new resources for in depth analysis of the evolution of the *map-1* gene family. In that respect, the objective of the present study was to further investigate its phylogenetic distribution and diversity into the Nematoda phylum, using a combination of bioinformatic and PCR-based molecular approaches. Here we present evidence that members of the *map-1* gene family are indeed TRGs, whose distribution is exclusively restricted to species belonging to the *Meloidogyne* genus. With the sole exception of *M. floridensis*, *map-1* genes were detected further in parthenogenetic mitotic RKN species only. Sequence analysis confirmed the modular architecture of the repetitive region of these genes, and allowed the characterization of three new members of the gene family. In addition, variation was observed in the distribution of gene variants among species, with one to five genes detected per species. However, individual repeats appeared extremely conserved both intra and interspecifically in terms of their primary (nucleotide) structure, which suggests that some strong evolutionary force is acting upon them. Based on these findings, we hypothesize that gene duplications together with domain shuffling events during diversification of clonal species likely accounts for the current distribution of *map-1* genes, and that these genes may be related, to some extent, to the acquisition of variable degrees of host specialization by RKNs.

## Results

### Phylogenetic Distribution of *Map-1* Homologous Sequences is Restricted to Clonal Root-knot Nematode Species

The *map-1* gene was originally identified in *M. incognita*, and a preliminary search among seven additional nematode taxa identified homologs in two closely related species only, i.e., *M. arenaria* and *M. javanica* [8]. To better address this issue, we have undertaken a molecular evolution study of the *map-1* family using bioinformatics and molecular biology approaches. In particular, sequences corresponding to all or the repeat parts of the *map-1* gene were used as queries in Blast sequence similarity searches of more extensive genomic DNA and EST databases now available in a wide range of nematode species. Under our search criteria, BlastN, BlastP and TblastX analyses against non-redundant nucleotide and/or EST databases at NCBI did not identify any discernable homologs outside of the RKN species *M. arenaria*, *M. incognita* and *M. javanica.* Similarly, except for the three latter species, no homolog could be identified when searching nematode-specific resources, either complete genome sequences at WormBase or extensive EST datasets at Nematode.net (Table S1). Together, results of these bioinformatic searches suggest that the taxonomic distribution of this gene family is restricted to some species of the *Meloidogyne* genus.

**Figure 2:**
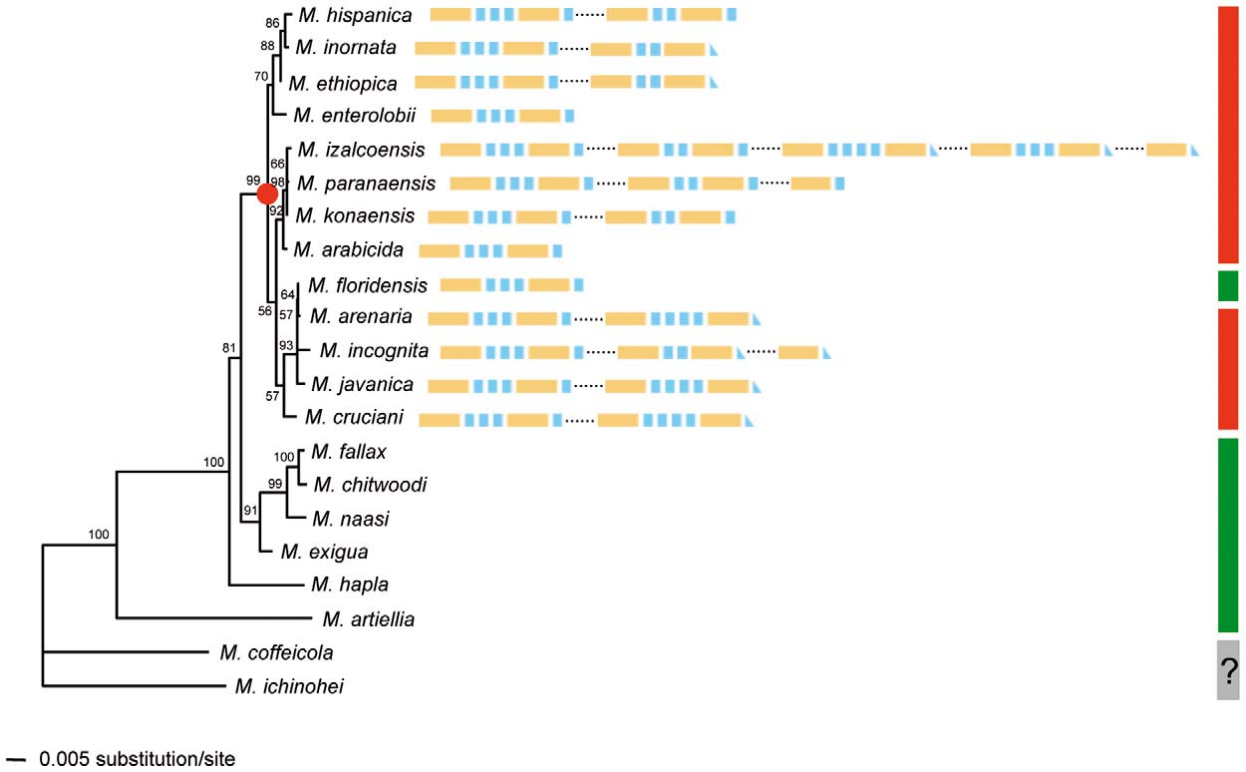
Distribution of *map-1* genes in root-knot nematodes. A neighbor-joining phylogenetic tree of the species used in this study was built using SSU rDNA sequences shown in Table S2. Bootstrap support was calculated from 1,000 replicates and values >50% are indicated at the corresponding nodes. The red dot shows the clade clustering species that reproduce by mitotic parthenogenesis, except for *M. floridensis.* Red and green lines show the species that reproduce by mitotic parthenogenesis or meiotic parthenogenesis/amphimixis, respectively. Orange and blue boxes represent the 58-aa and 13-aa domains in MAP-1 encoded proteins, respectively.

In parallel, we also developed a PCR approach to search for *map-1* homologous sequences in a total of 57 nematode species belonging to 12 families and seven orders. Cloning and sequencing of the obtained amplicons confirmed the presence of *map-1* homologs exclusively in RKNs, *Meloidogyne* spp. It is noticeable, however, that this distribution is not widespread in RKNs, but appeared to be restricted to 13 RKN species out of the 21 that were tested (Table 1). In order to infer the lineage specificity of the *map-1* homologs found in this study, we replaced their distribution in the phylogenetic framework of RKNs. For that purpose, we used SSU rDNA sequences, either available in databases or generated in the laboratory (Table S2) to build a *Meloidogyne* tree including the 21 RKN species investigated (Figure 2). This tree is fully consistent with phylogenies previously published for the genus [19]–[21]. In particular, a clade was individualized (with 99% bootstrap support) that clustered together RKN species that reproduce by mitotic parthenogenesis, except for *M. floridensis* (Figure 2). Using this species tree as the foundation, we further characterized the distribution of *map-1* genes among the genomes analyzed. Our results clearly showed that *map-1* homologs are exclusively present in all the species belonging to the clade mentioned above. With the exception of *M. floridensis*, no homologous sequence was detected in meiotic parthenogenetic species nor in *M. coffeicola* and *M. ichinohei*, both of which have a mode of reproduction that remains unknown (Figure 2).

**Table 1:** Nematode species used in this study, with taxonomic information based on [53], [54]. Presence (+) or absence (−) of *map-1* genes as inferred from PCR analysis.

Clade^a^	Order	Family	Species	*map-1*
I	Dorylaimida	Longidoridae	*Xiphinema index*	−
IV	Aphelenchida	Parasitaphelenchidae	*Bursaphelenchus mucronatus*	−
IV	Aphelenchida	Parasitaphelenchidae	*Bursaphelenchus xylophilus*	−
IV	Rhabditida	Steinernematidae	*Steinernema sp.*	−
IV	Tylenchida	Anguinidae	*Ditylenchus dipsaci*	−
IV	Tylenchida	Hoplolaimidae	*Globodera pallida*	−
IV	Tylenchida	Hoplolaimidae	*Globodera rostochiensis*	−
IV	Tylenchida	Hoplolaimidae	*Globodera sp.*	−
IV	Tylenchida	Hoplolaimidae	*Globodera tabacum*	−
IV	Tylenchida	Hoplolaimidae	*Heterodera avenae*	−
IV	Tylenchida	Hoplolaimidae	*Heterodera carotae*	−
IV	Tylenchida	Hoplolaimidae	*Heterodera filipjevi*	−
IV	Tylenchida	Hoplolaimidae	*Heterodera latipons*	−
IV	Tylenchida	Hoplolaimidae	*Heterodera sacchari*	−
IV	Tylenchida	Hoplolaimidae	*Heterodera schachtii*	−
IV	Tylenchida	Meloidogynidae	*Meloidogyne arabicida*	+
IV	Tylenchida	Meloidogynidae	*Meloidogyne arenaria*	+
IV	Tylenchida	Meloidogynidae	*Meloidogyne artiellia*	−
IV	Tylenchida	Meloidogynidae	*Meloidogyne chitwoodi*	−
IV	Tylenchida	Meloidogynidae	*Meloidogyne coffeicola*	−
IV	Tylenchida	Meloidogynidae	*Meloidogyne cruciani*	+
IV	Tylenchida	Meloidogynidae	*Meloidogyne enterolobii*	+
IV	Tylenchida	Meloidogynidae	*Meloidogyne ethiopica*	+
IV	Tylenchida	Meloidogynidae	*Meloidogyne exigua*	−
IV	Tylenchida	Meloidogynidae	*Meloidogyne fallax*	−
IV	Tylenchida	Meloidogynidae	*Meloidogyne floridensis*	+
IV	Tylenchida	Meloidogynidae	*Meloidogyne hapla*	−
IV	Tylenchida	Meloidogynidae	*Meloidogyne hispanica*	+
IV	Tylenchida	Meloidogynidae	*Meloidogyne ichinohei*	−
IV	Tylenchida	Meloidogynidae	*Meloidogyne incognita*	+
IV	Tylenchida	Meloidogynidae	*Meloidogyne inornata*	+
IV	Tylenchida	Meloidogynidae	*Meloidogyne izalcoensis*	+
IV	Tylenchida	Meloidogynidae	*Meloidogyne javanica*	+
IV	Tylenchida	Meloidogynidae	*Meloidogyne konaensis*	+
IV	Tylenchida	Meloidogynidae	*Meloidogyne naasi*	−
IV	Tylenchida	Meloidogynidae	*Meloidogyne paranaensis*	+
IV	Tylenchida	Pratylenchidae	*Hirschmaniella gracilis*	−
IV	Tylenchida	Pratylenchidae	*Nacobbus aberrans*	−
IV	Tylenchida	Pratylenchidae	*Pratylenchus coffeae*	−
IV	Tylenchida	Pratylenchidae	*Pratylenchus crenatus*	−
IV	Tylenchida	Pratylenchidae	*Pratylenchus mediterraneus*	−
IV	Tylenchida	Pratylenchidae	*Pratylenchus neglectus*	−
IV	Tylenchida	Pratylenchidae	*Pratylenchus penetrans*	−
IV	Tylenchida	Pratylenchidae	*Pratylenchus scribneri*	−
IV	Tylenchida	Pratylenchidae	*Pratylenchus thornei*	−
IV	Tylenchida	Pratylenchidae	*Pratylenchus vulnus*	−
IV	Tylenchida	Pratylenchidae	*Pratylenchus zeae*	−
IV	Tylenchida	Pratylenchidae	*Radopholus similis*	−
IV	Tylenchida	Pratylenchidae	*Radopholus sp.*	−
V	Rhabditida	Rhabditidae	*Caenorhabditis briggsae*	−
V	Rhabditida	Rhabditidae	*Caenorhabditis elegans*	−
V	Rhabditida	Rhabditidae	*Caenorhabditis remanei*	−
V	Rhabditida	Heterorhabditidae	*Heterorhabditis sp.*	−
V	Diplogasterida	Neodiplogasteridae	*Pristionchus pacificus*	−
V	Strongylida	Haemonchidae	*Haemonchus contortus*	−
V	Strongylida	Haemonchidae	*Teladorsagia circumcincta*	−
V	Strongylida	Trichostrongylidae	*Trichostrongylus colubriformis*	−

a Clades were denominated according to [55].

### Diversity of the *Map-1* Gene Family in Root-knot Nematodes

By monitoring the distribution of the *map-1* gene family across the Nematoda phylum, seven different members were identified in RKNs based on the architecture of the repeat region of the genes, among which four genes already described in literature [13], [22] and three new members of the family (Figure S2). Basically, the arrangement of the repeat region divided the genes into two groups, i.e., with a last short motif in 3′ orientation encoding a complete (13-aa) or a truncated (5-aa) repeat in MAP-1, respectively. Depending on the *Meloidogyne* species considered, one to five members of the family were detected, with a maximum number in M. izalcoensis. Interestingly, the *map-1.1* gene was found present in all the 13 RKN species containing one or several *map-1* homologs.

**Figure 3:**
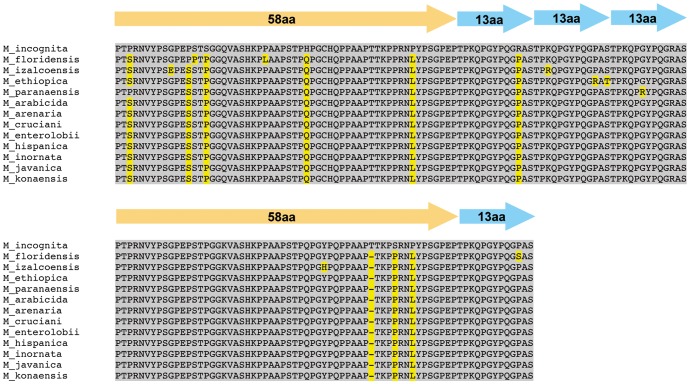
Multiple alignment of the repetitive domains of the MAP-1.1 deduced protein sequences from *Meloidogyne* spp. Dashes indicate deletions. Yellow boxes indicate mutated positions compared to the MAP-1.1 sequence from *M. incognita.*

To analyze deeper the variability of the *map-1* gene family in RKNs, we compared the deduced amino acid sequences resulting from the translation of the repeat region of the *map-1.1* genes previously cloned in these 13 RKN species. Sequence alignments clearly showed a high level of global conservation at the amino acid level (i.e., ∼94.3%) as estimated by the ratio of conserved vs. modified positions in the 12 RKN species compared to the *M. incognita* MAP-1.1 sequence (Figure 3). More precisely, six substitutions and one deletion were common to the 12 RKN species, while two additionnal substitutions were shared by 11 of the 12 RKN species, respectively. Moreover, nine substitutions present in one single RKN species were distributed in four species, i.e., *M. ethiopica*, *M. floridensis*, *M. izalcoensis* and *M. paranaensis* (Figure 3).

### Sequence Conservation of *Map-1.1* in *Meloidogyne incognita*

We further evaluated the infraspecific polymorphism of the *map-1* gene in *M. incognita*. For that purpose, a genomic fragment encompassing the *map-1.1* repetitive region and its 5′ and 3′ flanking regions was amplified and directly sequenced from individual nematodes belonging to 16 *M. incognita* isolates from different geographic origins, either avirulent or virulent against the tomato *Mi* resistance gene (Table S3). Readable sequences were obtained for 122 individual nematodes, with five to ten sequences per isolate (Table S3). After trimming, these sequences could be unambiguously aligned over 591 bp, and showed a very high level of identity between them (Figure S1). Overall, compared to the original *map-1.1* sequence (accession FM164760) [13], 13 positions over 591 (i.e., about 2.2%) exhibited mutations (either substitutions or deletions) and two additional single-bp insertions were observed. Variations were not equally distributed along the sequence analyzed: 1 single-bp insertion and one deletion (in 2 individuals over 122) were found in the 5′ flanking region, 5 mutations (in 9 individuals over 122) in the 3′ flanking region, while the remaining polymorphism was found concentrated in the region encoding the first 58-aa repeat (in 50 individuals over 122). Conversely, nucleotides encoding the second 58-aa repeat, and the four 13-aa repeats, appeared perfectly conserved in all the 122 individual nematodes tested (Figure S1). On the whole data set, no correlation was observed between the distribution of mutations and the (a)virulence of the isolates.

## Discussion

In this study, we made use of greatly expanded sequence data sets from nematode genomes, in combination with PCR experiments, to look for homologs, in the Nematoda phylum, of the *map-1* gene originally identified in the RKN species *M. incognita* [8]. *Map-1* genes were identified in a single member of the phylum Nematoda, i.e., the genus Meloidogyne, and not detected in any other nematode, including additional species of the same clade. Moreover, the distribution of these genes appeared strictly restricted to but patchy within the *Meloidoygne* genus, with *map-1* homologs found in 13 RKN species among the 21 experimentally tested. Based on these results, and in good agreement with very preliminary data [8], we concluded that the *map-1* gene family is indeed a TRG family. Obviously, such a highly restricted pattern of distribution raises question about the origin of the *map-1* gene ‘founder’, i.e., the gene that gave rise to this gene family in descendant lineages [23]. The major molecular mechanisms leading to the emergence of novel TRGs basically consist in duplication events and rearragement processes followed by fast divergence, or by de novo origination from non-coding sequences via mutations [reviewed in 24]. Alternatively, another possibility would be that *map-1* was acquired by RKNs via a horizontal gene transfer (HGT) event. Such a possibility appears all the more attractive as RKNs are prone to undergo HGT, as recently demonstrated for the functional acquisition of a large repertoire of plant cell wall-degrading enzymes from bacterial origin by these nematodes [9], [25]. However, in this hypothesis, the *map-1* donator organism remains unknown.

Interestingly, a more careful phylogenetic analysis of the distribution of *map-1* genes in RKNs further indicated that these genes are specifically present in all the members of a sub-clade clustering together the species that reproduce by mitotic parthenogenesis, with the exception of *M. floridensis*. Such a congruence between the evolutionary relationship of map-1 genes and the species phylogeny is a good indication that all the genes from the family are true orthologs. The observation that *map-1* was indeed detected in *M. floridensis* is not that surprising, since previous phylogenetic studies have shown that this species is part of the so-called clade I grouping together the mitotic RKN species, and probably results from an independent transition to meiotic parthenogenesis [19], [20], [26]. Conversely, *map-1* genes could not be detected in RKNs reproducing by either meiotic parthenogenesis or amphimixis. By June 2009, there were 97 valid species in the *Meloidogyne* genus [27], and we conducted experiments on only 21 of them. Thus, in order to provide a more exhaustive view of *map-1* representation within the genus, it will be important to examine more species as well. However, whatever its origin, we can hypothesize that the current distribution of *map-1* could result either from the acquisition of the founder gene by the common ancestor of RKNs, with a subsequent loss of the gene in meiotic species after their divergence from mitotic ones, or from a later acquisition of the founder gene by the mitotic ancestor after such a divergence. Although the early or late origin of clonality in RKNs is still under debate [5]–[7], the present results highlight the divergence between mitotic and meiotic species as a critical transition in the evolutionary history of these parasites.

**Figure 4:**
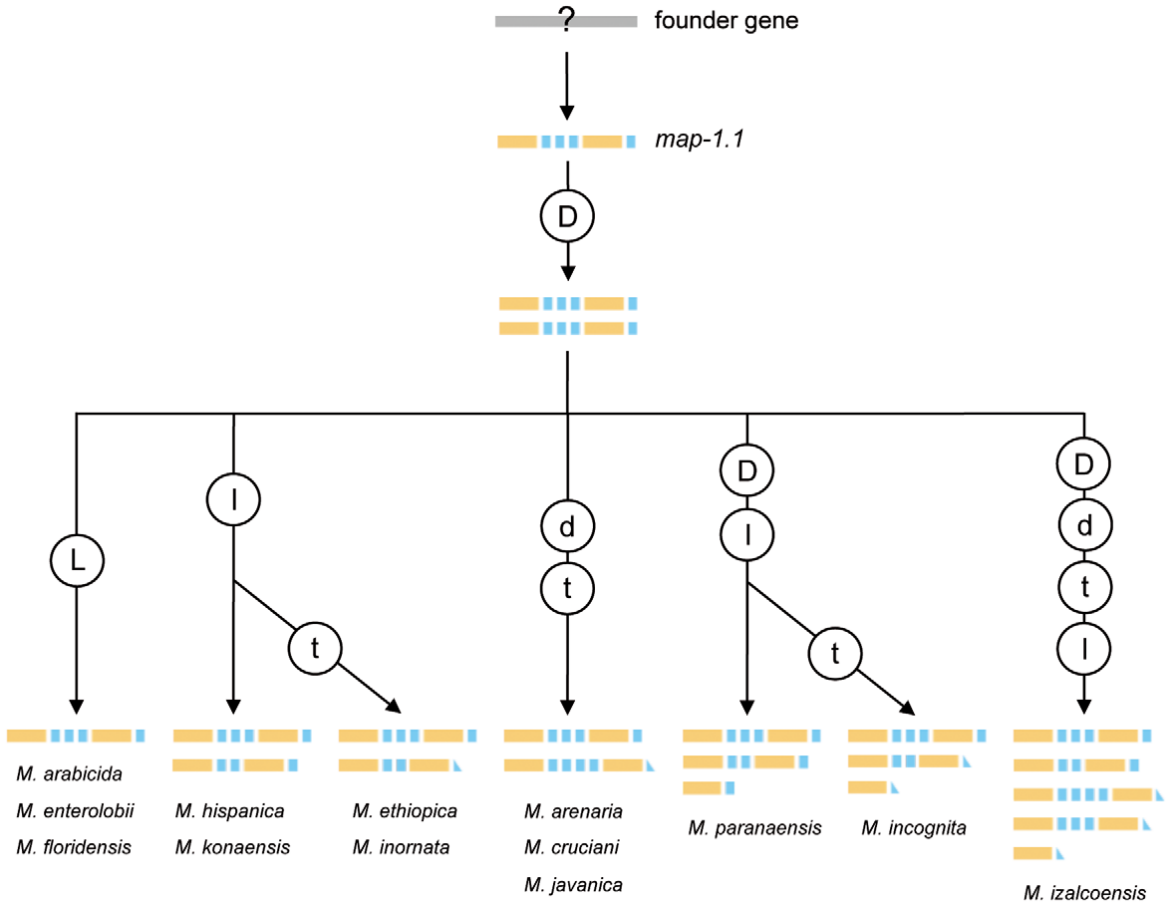
Hypothetical model for the evolution of *map-1* genes in root-knot nematodes. The actual distribution of *map-1* genes in *Meloidogyne* species is indicated at the bottom of the figure. Putative individual evolutionary events leading to such a distibution are indicated in circles according to the following code: D, L  =  gene duplication or loss, respectively; d, l, t  =  domain duplication, loss or truncation, respectively. Orange and blue boxes represent the 58-aa and 13-aa domains in MAP-1 encoded proteins, respectively.

Three new members of the *map-1* gene family have been identified in this work (i.e., genes encoding new combinations of the 58-aa and 13-aa motifs, respectively), that add up to the four members already described in *M. incognita* and *M. javanica* [8], [22], and confirm the modular architecture of the MAP-1 proteins. Many exemples of multi-domain proteins from higher eukaryotes have been described in literature [e.g., 28–31], and several molecular mechanisms are known to be involved in the creation of new gene structures including duplicated and rearranged modular domains [for review 32]. In our case, only the *map-1.1* form was present in all the RKN species harbouring map-1 gene(s) in their genome, while the other forms of the genes appeared randomly distributed. To this point, and although its architecture is not the simplest that could be designed, our current hypothesis is that *map-1.1* is presumably the closest to the founder gene of the entire family, and thus all the other forms of the gene are supposed to have evolved from it following duplication events. Indeed, gene duplications are considered as the primary source of new genes in nematodes [33], [34]. In the light of this hypothesis, and after initial or secondary gene duplication (or gene loss in the case of *M. arabicida*, *M. enterolobii* and *M. floridensis*), the observed organization of motifs within the entire set of MAP-1 proteins from closely related RKN species could result from 1) duplication or loss of one single 13-aa repeat; 2) truncation of the N-terminal 13-aa repeat; and 3) loss of both 58 and 13-aa repeats (in *M. incognita*, *M. izalcoensis* and *M. paranaensis*) (Figure 4). Such an evolutionary scenario at the sub-gene level involving duplication and/or loss of domains has already been documented in higher eukaryotes, for exemple in the case of snake venom metalloproteinase toxin genes [35], zinc finger genes in mammals [36] or sperm-egg-binding proteins in ancestral primates [37]. It also confirms and expand previous data obtained on *M. incognita* only [13]. However, considering the phylogenetic distribution of *map-1* genes among RKN species, whether such evolutionary steps occurred simultaneously or independently remains to be determined.

Comparison of the repetitive region encompassing the 58 and 13-aa domains of the MAP-1.1 proteins between all 13 RKN species harbouring the corresponding gene(s) revealed a high sequence conservation. Even more remarkably, the *map-1.1* genes amplified from more than one hundred individual nematodes belonging to 16 *M. incognita* isolates exhibited an extreme similarity in their repetitive region at the nucleotide level. Alltogether, and although further analyses are needed to confirm it definitely, this observation constitutes a reasonable indication that some purifying selection mechanism may be acting upon the gene to maintain its functional role(s). In parallel with their extensive diversity in terms of modes of reproduction, RKNs also demonstrate various degrees of specialization with respect to their host preference, with amphimictic and meiotic species having a narrow host range, while the potential host range of mitotic species encompasses the vast majority of higher plants [2], [38], [39]. Recent immunolocalisation experiments have shown that MAP-1 proteins are secreted within plant tissues during infection, whose active accumulation in the apoplasm could play a role in the recognition between the plant and the parasite [11], [12]. Do *map-1* genes act to modulate conserved plant-nematode interaction mechanisms, and/or do they provide differential species-specific parasitic abilities? Resolution of these issues require to experimentally test the biochemical or phenotypic function(s) of *map-1* genes, and to understand how selection works during their formation and evolution. Clearly, further investigation and understanding of these questions will continue to depend on theoretical and careful functional analyses of each individual member of the *map-1* gene family.

## Materials and Methods

### Biological Material and Isolation of Nucleic Acids

Samples from *Bursaphelenchus*, *Caenorhabditis*, *Meloidogyne* and *Xiphinema* genera were selected from the nematode living collection at INRA, Sophia Antipolis, France. In addition, some nematodes or DNA samples were generously provided by Géraldine Anthoine, Carla De Giorgi, Godelieve Gheysen, Eric Grenier, Hans Helder, Maurice Moens, Cédric Neveu, Ralf Sommer and Myrian Tigano. Genomic DNA was prepared from ca. 200 µl of nematodes according to a standard phenol/chlorophorm protocol [40] and stored at −80°C until use. In cases where very little amounts of biological material were available, crude extracts from individual nematodes were prepared as described previously [41]. Before PCR analyses, genomic DNA samples or crude extracts from individual nematodes were diluted to 0.1–0.5 ng.µl^−1^ or 20 times, respectively. Quality of DNA was checked by PCR using the universal/nematode primers 988F and 1912R [42] (Table 2). In the case of RKNs, sample identity to the species level was further confirmed using either isozyme electrophoretic analyses [43] or species-specific SCAR markers [41], [44]–[46].

**Table 2:** Primers used in this study.

Name	Sequence (5′ to 3′)	Reference
m1	CGTGTAACAGAGATGCCAGA	[8]
m2	GTGGAGGAACAGTAAGTGAG	[8]
MRepF	GCCAGAATCATCTACACCAGGAGGACA	this study
MRepR	TGTTGTTGGAGCTGCTGGTGGTT	this study
988F	CTCAAAGATTAAGCCATGC	[42]
1912R	TTTACGGTCAGAACTAGGG	[42]
1813F	CTGCGTGAGAGGTGAAAT	[42]
2646R	GCTACCTTGTTACGACTTTT	[42]

### PCR Analyses and Sequencing

Primer sequences used in this work are listed in Table 2. Reactions were performed in a final volume of 25 µl containing 200 µM dNTPs, 0.4 µM of primers, 1 U *Taq* DNA polymerase and 1x *Taq* DNA polymerase reaction buffer supplemented with MgCl_2_ to a final concentration of 1.5 mM according to the manufacturer’s guidelines (MP Biomedicals Taq Core kit). For the search of *map-1* homologous sequences, two pairs of primers were successively used. Primers m1 and m2 amplify the central region of the *map-1.2* gene encompassing the internal repeats in *M. incognita*, while primers MrepF and MrepR were designed at both ends of the region encoding the 58 amino-acid repeat in the MAP-1 protein (Fig. 1; Table 2). For m1/m2, cycling conditions were: 94°C for 5 min; 32x (94°C, 1 min; 58°C, 1 min; 72°C, 1 min 30 s); 72°C, 5 min. For MrepF/MrepR, cycling conditions were: 94°C for 3 min; 35x (94°C, 30 s; 54°C, 30 s; 72°C, 1 min); 72°C, 5 min. For phylogenetic analysis of RKNs, small-subunit (SSU) rDNA was amplified as two partially overlapping fragments using three universal (988F, 1813F and 2646R) and one nematode-specific (1912R) primer (Table 2) according to previously published PCR conditions [42].

Amplicons were analysed by electrophoresis in 2% agarose gels containing ethidium bromide in TBE buffer, and visualized under UV light. The selected amplicons were recovered to gel using the MiniElute Gel Extraction kit (Qiagen), ligated into a pGEM T-Easy Vector System (Promega) and transformed in *Escherichia coli* DH10ß competent cells. Recombinant clones were sequenced on both strands by GATC Biotech (Konstanz, Germany).

### EMBL Databank Deposits

The *Meloidogyne* spp. sequences obtained in this work using the m1/m2 primer pair were deposited in EMBL databank under Accession Numbers HE681888-HE681910. Newly generated *Meloidogyne* spp. SSU rDNA sequences were deposited in EMBL databank under Accession Numbers HE667738-HE667744.

### Bioinformatic Analyses

BlastN, BlastP and TBlastX search strategies [47] were conducted to identify DNA and/or protein sequences homologous to *map-1* in non-redundant nucleotide, EST and protein databases at the National Center for Biotechnology Information (NCBI at http://www.ncbi.nlm.nih.gov), using sequences corresponding to all or repeat parts of the *map-1.2* gene (accession AJ278663), and their theoretical amino acid sequence translations, as queries. At the nucleotide level, additional BlastN searches were performed against a set of nematode-specific databases, i.e., completed genomic sequences of 11 species at WormBase (http://www.wormbase.org) and EST clusters of 43 species at Nematode.net (http://www.nematode.net) [48]. For the complete list of the nematode species investigated, see Table 1. Putative homologs were checked for significance manually, and only sequences that aligned at least on 60% of the length of the query sequence with an E**-**value of 1.e^−05^ as the cutoff were considered as true homologs.

Nucleotide sequences were edited and translated into amino acid sequences using ExPASy online tools [49] at http://www.expasy.org. Nucleotide and amino acid sequences were aligned using MUSCLE v3.7 [50] with standard parameters at http://www.ebi.ac.uk, and alignments were refined by eye when necessary. Phylogenetic analyses were done using the Neighbor-Joining algorithm as implemented in PAUP* [51], based on mean character difference as genetic distance. Bootstrap resampling [52] with 1,000 pseudoreplicates was carried out to assess support for each individual branch.

## Supporting Information

Figure S1 **Alignment of** ***map-1.1*** **partial sequences amplified from 122** ***Meloidogyne incognita*** **individuals belonging to 16 isolates.** Nomenclature of isolates is as in Table S3. Stars below alignment indicate conserved positions. Orange and blue sequences above alignment encode the 58 and 13-aa repeat regions, respectively. Sequences encoding the tandemly arranged 13-aa repeats are separated by arrows. Mutated positions are highlighted in yellow.
(PDF)

Figure S2 **Distribution and structural organisation of** ***map-1*** **genes in root-knot nematodes.** The orange and blue boxes correspond to regions encoding the 58-aa and 13-aa repeats in MAP-1, respectively. The blue triangles indicate regions encoding the 13-aa truncated repeats.
(PDF)

Table S1 **List of the nematode-specific nucleotide resources used in BlastN analysis.** Grey shading identifies the species in which *map-1* homologs were found.
(PDF)

Table S2 **List of the rDNA SSU sequences used to infer phylogenetic relationships of root-knot nematodes.**
(DOCX)

Table S3 **List of the** ***Meloidogyne incognita*** **isolates used in this study, with their geographic origin and virulence status against the tomato** ***Mi-1*** **resistance gene.**
(PDF)

## References

[1] Blaxter ML (2003). Nematoda: Genes, genomes and the evolution of parasitism.. Adv Parasitol.

[2] Blok VC, Jones JT, Phillips MS, Trudgill DL (2008). Parasitism genes and host range disparities in biotrophic nematodes: the conundrum of polyphagy versus specialisation.. BioEssays.

[3] Abad P, Favery B, Rosso MN, Castagnone-Sereno P (2003). Root-knot nematode parasitism and host response: molecular basis of a sophisticated interaction.. Mol Plant Pathol.

[4] Triantaphyllou AC, Sasser JN, Carter CC (1985). Cytogenetics, cytotaxonomy and phylogeny of root-knot nematodes.. Raleigh: North Carolina State University Graphics.

[5] Castagnone-Sereno P (2006). Genetic variability and adaptive evolution in parthenogenetic root-knot nematodes.. Heredity.

[6] Lunt DH (2008). Genetic tests of ancient asexuality in root-knot nematodes reveal recent hybrid origins.. BMC Evol Biol.

[7] Fargette M, Berthier K, Richaud M, Lollier V, Franck P (2010). Crosses prior to parthenogenesis explain the current genetic diversity of tropical plant-parasitic *Meloidogyne* species (Nematoda: Tylenchida).. Infect Genet Evol.

[8] Semblat JP, Rosso MN, Hussey RS, Abad P, Castagnone-Sereno P (2001). Molecular cloning of a cDNA encoding an amphid-secreted putative avirulence protein from the root-knot nematode *Meloidogyne incognita.*. Mol Plant Microbe Interact.

[9] Danchin EGJ, Rosso MN, Vieira P, de Almeida-Engler J, Coutinho PM (2010). Multiple lateral gene transfers and duplications have promoted plant parasitism ability in nematodes.. Proc Natl Acad Sci USA.

[10] Qin L, Kudla U, Roze EHA, Goverse A, Popeijus H (2004). A nematode expansin acting on plants.. Nature.

[11] Rosso MN, Vieira P, de Almeida-Engler J, Castagnone-Sereno P (2011). Proteins secreted by root-knot nematodes accumulate in the extracellular compartment during root infection.. Plant Signal Behav.

[12] Vieira P, Danchin EGJ, Neveu C, Crozat C, Jaubert S (2011). The plant apoplasm is an important recipient compartment for nematode secreted proteins.. J Exp Bot.

[13] Castagnone-Sereno P, Semblat JP, Castagnone C (2009). Modular architecture and evolution of the *map-1* gene family in the root-knot nematode *Meloidogyne incognita.*. Mol Genet Genomics.

[14] Wilson GA, Bertrand N, Patel Y, Hughes JB, Feil EJ (2005). Orphans as taxonomically restricted and ecologically important genes.. Microbiology.

[15] Khalturin K, Hemmrich G, Fraune S, Augustin R, Bosch TCG (2009). More than just orphans: are taxonomically-restricted genes important in evolution?. Trends Genet.

[16] Johnson BR, Tsutsui ND (2011). Taxonomically restricted genes are associated with the evolution of sociality in the honey bee.. BMC Genomics.

[17] Abad P, Gouzy J, Aury JM, Castagnone-Sereno P, Danchin EG (2008). Genome sequence of the metazoan plant-parasitic nematode *Meloidogyne incognita.*. Nat Biotechnol.

[18] Opperman CH, Bird DM, Williamson VM, Rokhsar DS, Burke M (2008). Sequence and genetic map of *Meloidogyne hapla:* A compact nematode genome for plant parasitism.. Proc Natl Acad Sci USA.

[19] Tigano M, Carneiro R, Jeyaprakash A, Dickson D, Adams B (2005). Phylogeny of *Meloidogyne* spp.. based on 18S rDNA and mitochondrial DNA partial sequences Nematology.

[20] Holterman M, Karssen G, van den Elsen S, van Megen H, Bakker J (2009). Small subunit rDNA-based phylogeny of the Tylenchida sheds light on relationships among some high-impact plant-parasitic nematodes and the evolution of plant feeding.. Phytopathology.

[21] Van Megen H, van den Elsen S, Holterman M, Karssen G, Mooyman P (2009). A phylogenetic tree of nematodes based on about 1200 full-length small subunit ribosomal DNA sequences.. Nematology.

[22] Adam MAM, Phillips MS, Tzortzakakis EA, Blok VC (2009). Characterisation of mjap genes encoding novel secreted proteins from the root-knot nematode *Meloidogyne javanica.*. Nematology.

[23] Domazet-Loso T, Josip Brajkovic J, Tautz D (2007). A phylostratigraphy approach to uncover the genomic history of major adaptations in metazoan lineages.. Trends Genet.

[24] Tautz D, Domazet-Loso T (2011). The evolutionary origin of orphan genes.. Nature Rev Genet.

[25] Haegeman A, Jones JT, Danchin EGJ (2011). Horizontal gene transfer in nematodes: a catalyst for plant parasitism?. Mol Plant Microbe Interact.

[26] Denver DR, Clark KA, Raboin MJ (2011). Reproductive mode evolution in nematodes: Insights from molecular phylogenies and recently discovered species.. Mol Phylogenet Evol.

[27] Hunt DJ, Handoo ZA, Perry RN, Moens M, Starr JL (2009). Taxonomy, identification and principal species..

[28] Apic G, Huber W, Teichmann SA (2003). Multi-domain protein families and domain pairs: comparison with known strutures and a random model of domain recombination.. J Struct Funct Genomics.

[29] Garb JE, Hayashi CY (2005). Modular evolution of egg case silk genes across orb-weaving spider superfamilies.. Proc Natl Acad Sci USA.

[30] Rasteiro R, Pereira-Leal JB (2007). Multiple domain insertions and losses in the evolution of the Rab prenylation complex.. BMC Evol Biol.

[31] Wu YC, Rasmussen MD, Kellis M (2012). Evolution at the subgene level: domain rearrangements in the *Drosophila* phylogeny.. Mol Biol Evol.

[32] Moore AD, Björklund AK, Ekman D, Bornberg-Bauer E, Elofsson A (2008). Arrangements in the modular evolution of proteins.. Trends Biochem Sci.

[33] Katju V, Lynch M (2006). On the formation of novel genes by duplication in the *Caenorhabditis elegans* genome.. Mol Biol Evol.

[34] Lipinski KJ, Farslow JC, Fitzpatrick KA, Lynch M, Katju V (2011). High spontaneous rate of gene duplication in *Caenorhabditis elegans.*. Curr Biol.

[35] Casewell NR, Wagstaff SC, Harrison RA, Renjifo C, Wuester W (2011). Domain loss facilitates accelerated evolution and neofunctionalization of duplicate snake venom metalloproteinase toxin genes.. Mol Biol Evol.

[36] Tadepally HD, Burger G, Aubry M (2008). Evolution of C2H2-zinc finger genes and subfamilies in mammals: Species-specific duplication and loss of clusters, genes and effector domains.. BMC Evol Biol.

[37] Podlaha O, Webb DM, Zhang J (2006). Accelerated evolution and loss of a domain of the sperm-egg-binding protein SED1 in ancestral primates.. Mol Biol Evol.

[38] Trudgill DL, Blok VC (2001). Apomictic, polyphagous root-knot nematodes: exceptionally successful and damaging biotrophic root pathogens.. Annu Rev Phytopathol.

[39] Moens M, Perry RN, Starr JL, Perry RN, Moens M, Starr JL (2009). *Meloidogyne* species – a diverse group of novel and important plant parasites..

[40] Sambrook J, Fritsch E F, Maniatis T (1989). Molecular cloning: a laboratory manual (2nd ed).. Cold Spring Harbor, NY: Cold Spring Harbor Laboratory.

[41] Tigano M, de Siqueira K, Castagnone-Sereno P, Mulet K, Queiroz P (2010). Genetic diversity of the root-knot nematode *Meloidogyne enterolobii* and development of a SCAR marker for this guava-damaging species.. Plant Pathol.

[42] Holterman M, Van der Wurff A, Van den Elsen S, Van Megen H, Bongers T (2006). Phylum-wide analysis of SSU rDNA reveals deep phylogenetic relationships among nematodes and accelerated evolution toward crown clades.. Mol Biol Evol.

[43] Carneiro RMDG, Almeida MRA, Quénéhervé P (2000). Enzyme phenotype of *Meloidogyne* spp. populations.. Nematology.

[44] Randig O, Bongiovanni M, Carneiro MDG, Castagnone-Sereno P (2002). Genetic diversity of root-knot nematodes from Brasil and development of SCAR markers specific for the coffee-damaging species.. Genome.

[45] Zijlstra C (2000). Identification of *Meloidogyne chitwoodi*, *M. fallax* and *M. hapla* based on SCAR–PCR: a powerful way of enabling reliable identification of populations or individuals that share common traits.. Eur J Plant Pathol.

[46] Zijlstra C, Donkers-Venne DTHM, Fargette M (2000). Identification of *Meloidogyne incognita*, *M. javanica* and *M. arenaria* using sequence characterised amplified regions (SCAR) based PCR assays.. *Nematology*.

[47] Zhang Z, Schwartz S, Wagner L, Miller W (2000). A greedy algorithm for aligning DNA sequences.. J Comput Biol.

[48] Martin J, Abubucker S, Wylie T, Yin Y, Wang Z (2009). Nematode.net update 2008: improvements enabling more efficient data mining and comparative nematode genomics.. Nucleic Acids Res.

[49] Gasteiger E, Hoogland C, Gattiker A, Duvaud S, Wilkins MR, Walker JM (2005). Protein identification and analysis tools on the ExPASy server..

[50] Edgar RC (2004). MUSCLE: multiple sequence alignment with high accuracy and high throughput. Nucleic Acids Res..

[51] Swofford DL (1998). PAUP*: (Phylogenetic Analysis Using Parsimony *) and other methods Version 4 Sinauer Associates, Sunderland Mass..

[52] Felsenstein J (1985). Confidence limits on phylogenies: An approach using the bootstrap.. Evolution.

[53] Siddiqi MR (2000). Tylenchida: Parasites of plants and insects. Second edition. Wallingford, UK: CAB International Publishing.. 833 p.

[54] De Ley P, Blaxter ML, Cook R, Hunt DJ (2004). A new system for Nematoda: combining morphological characters with molecular trees, and translating clades into ranks and taxa..

[55] Blaxter ML, De Ley P, Garey JR, Liu LX, Scheldeman P (1998). A molecular evolutionary framework for the phylum Nematoda.. Nature.

