# Supplementary material for: The map-1 Gene Family in Root-Knot Nematodes, Meloidogyne spp.: A Set of Taxonomically Restricted Genes Specific to Clonal Species
Source: PLoS One. 2012 Jun 18;7(6):e38656. doi: 10.1371/journal.pone.0038656 (PMC3377709; doi:10.1371/journal.pone.0038656)
Supplement: Figure S1 — Alignment of map-1.1 partial sequences amplified from 122 Meloidogyne incognita individuals belonging to 16 isolates. Nomenclature of isolates is as in Table S3. Stars below alignment indicate conserved positions. Orange and blue sequences above alignment encode the 58 and 13-aa repeat regions, respectively. Sequences encoding the tandemly arranged 13-aa repeats are separated by arrows. Mutated positions are highlighted in yellow. (PDF) [file pone.0038656.s001.pdf]

Figure S1. Alignment of *map-1.1* partial sequences amplified from 122 *Meloidogyne incognita* individuals belonging to 16 isolates. Nomenclature of isolates is as in Table S3. Stars below alignment indicate conserved positions. Orange and blue sequences above alignment encode the 58 and 13-aa repeat regions, respectively. Sequences encoding the tandemly arranged 13-aa repeats are separated by arrows. Mutated positions are highlighted in yellow.

```
                         CCAACTTCACGAAACGTTTACCCATCTGGGCCAGAATCATCTACACCAGGAGGACAAGTAGCATCGCATAAG-CCTCCGGCAGCACCTTCTACTCCACAGCCTGGATGTCA
Minc_18_1     TACAACAGG-AGCTCAACCAACTTCACGAAACGTTTACCCATCTGGGCCAGAATCATCTACACCAGGAGGACAAGTAGCATCGCATAAG-CCTCCGGCAGCACCTTCTACTCCACAGCCTGGATGTCA
Minc_18_10    TACAACAGG-AGCTCAACCAACTTCACGAAACGTTTACCCATCTGGGCCAGAATCATCTACACCAGGAGGACAAGTAGCATCGCATAAG-CCTCCGGCAGCACCTTCTACTCCACAGCCTGGATGTCA
Minc_18_11    TACAACAGG-AGCTCAACCAACTTCACGAAACGTTTACCCATCTGGGCCAGAATCATCTACACCAGGAGGACAAGTAGCATCGCATAAG-CCTCCGGCAGCACCTTCTACTCCACAGCCTGGATGTCA
Minc_18_13    TACAACAGG-AGCTCAACCAACTTCACGAAACGTTTACCCATCTGGGCCAGAATCATCTACACCAGGAGGACAAGTAGCATCGCATAAG-CCTCCGGCAGCACCTTCTACTCCACAGCCTGGATGTCA
Minc_18_3     TACAACAGG-AGCTCAACCAACTTCACGAAACGTTTACCCATCTGGGCCAGAATCATCTACACCAGGAGGACAAGTAGCATCGCATAAG-CCTCCGGCAGCACCTTCTACTCCACAGCCTGGATGTCA
Minc_18_4     TACAACAGG-AGCTCAACCAACTTCACGAAACGTTTACCCATCTGGGCCAGAATCATCTACACCAGGAGGACAAGTAGCATCGCATAAG-CCTCCGGCAGCACCTTCTACTCCACAGCCTGGATGTCA
Minc_18_5     TACAACAGG-AGCTCAACCAACTTCACGAAACGTTTACCCATCTGGGCCAGAATCATCTACACCAGGAGGACAAGTAGCATCGCATAAG-CCTCCGGCAGCACCTTCTACTCCACAGCCTGGATGTCA
Minc_18_7     TACAACAGG-AGCTCAACCAACTTCACGAAACGTTTACCCATCTGGGCCAGAATCATCTACACCAGGAGGACAAGTAGCATCGCATAAG-CCTCCGGCAGCACCTTCTACTCCACAGCCTGGATGTCA
Minc_18_8     TACAACAGG-AGCTCAACCAACTTCACGAAACGTTTACCCATCTGGGCCAGAATCATCTACACCAGGAGGACAAGTAGCATCGCATAAG-CCTCCGGCAGCACCTTCTACTCCACAGCCTGGATGTCA
Minc_18_9     TACAACAGG-AGCTCAACCAACTTCACGAAACGTTTACCCATCTGGGCCAGAATCATCTACACCAGGAGGACAAGTAGCATCGCATAAG-CCTCCGGCAGCACCTTCTACTCCACAGCCTGGATGTCA
Minc_22_14    TACAACAGG-AGCTCAACCAACTTCACGAAACGTTTACCCATCTGGGCCAGAATCATCTACACCAGGAGGACAAGTAGCATCGCATAA---CTCCGGCAGCACCTTCTACTCCACAGCCTGGATGTCA
Minc_22_15    TACAACAGG-AGCTCAACCAACTTCACGAAACGTTTACCCATCTGGGCCAGAATCATCTACACCAGGAGGACAAGTAGCATCGCATAAC---TCCGGCAGCACCTTCTACTCCACAGCCTGGATGTCA
Minc_22_2     TACAACAGG-AGCTCAACCAACTTCACGAAACGTTTACCCATCTGGGCCAGAATCATCTACACCAGGAGGACAAGTAGCATCGCATAA---CTCCGGCAGCACCTTCTACTCCACAGCCTGGATGTCA
Minc_22_3     TACAACAGG-AGCTCAACCAACTTCACGAAACGTTTACCCATCTGGGCCAGAATCATCTACACCAGGAGGACAAGTAGCATCGCATAAG---TCCGGCAGCACCTTCTACTCCACAGCCTGGATGTCA
Minc_22_5     TACAACAGG-AGCTCAACCAACTTCACGAAACGTTTACCCATCTGGGCCAGAATCATCTACACCAGGAGGACAAGTAGCATCGCATAAC---TCCGGCAGCACCTTCTACTCCACAGCCTGGATGTCA
Minc_22_7     TACAACAGG-AGCTCAACCAACTTCACGAAACGTTTACCCATCTGGGCCAGAATCATCTACACCAGGAGGACAAGTAGCATCGCATA----CTCCGGCAGCACCTTCTACTCCACAGCCTGGATGTCA
Minc_22_8     TACAACAGG-AGCTCAACCAACTTCACGAAACGTTTACCCATCTGGGCCAGAATCATCTACACCAGGAGGACAAGTAGCATCGCATAA---CTCCGGCAGCACCTTCTACTCCACAGCCTGGATGTCA
Minc_30_1     TACAACAGG-AGCTCAACCAACTTCACGAAACGTTTACCCATCTGGGCCAGAATCATCTACACCAGGAGGACAAGTAGCATCGCATAAC---TCCGGCAGCACCTTCTACTCCACAGCCTGGATGTCA
Minc_30_10    TACAACAGG-AGCTCAACCAACTTCACGAAACGTTTACCCATCTGGGCCAGAATCATCTACACCAGGAGGACAAGTAGCATCGCATAA---CTCCGGCAGCACCTTCTACTCCACAGCCTGGATGTCA
Minc_30_11    TACAACAGG-AGCTCAACCAACTTCACGAAACGTTTACCCATCTGGGCCAGAATCATCTACACCAGGAGGACAAGTAGCATCGCATAAC---TCCGGCAGCACCTTCTACTCCACAGCCTGGATGTCA
Minc_30_4     TACAACAGG-AGCTCAACCAACTTCACGAAACGTTTACCCATCTGGGCCAGAATCATCTACACCAGGAGGACAAGTAGCATCGCATAAC---GCCGGCAGCACCTTCTACTCCACAGCCTGGATGTCA
Minc_30_5     TACAACAGG-AGCTCAACCAACTTCACGAAACGTTTACCCATCTGGGCCAGAATCATCTACACCAGGAGGACAAGTAGCATCGCATAA---CTCCGGCAGCACCTTCTACTCCACAGCCTGGATGTCA
Minc_34_1     TACAACAGG-AGCTCAACCAACTTCACGAAACGTTTACCCATCTGGGCCAGAATCATCTACACCAGGAGGACAAGTAGCATCGCATAAC---TCCGGCAGCACCTTCTACTCCACAGCCTGGATGTCA
Minc_34_10    TACAACAGG-AGCTCAACCAACTTCACGAAACGTTTACCCATCTGGGCCAGAATCATCTACACCAGGAGGACAAGTAGCATCGCATAAC---TCCGGCAGCACCTTCTACTCCACAGCCTGGATGTCA
Minc_34_11    TACAACAGG-AGCTCAACCAACTTCACGAAACGTTTACCCATCTGGGCCAGAATCATCTACACCAGGAGGACAAGTAGCATCGCATAA---CTCCGGCAGCACCTTCTACTCCACAGCCTGGATGTCA
Minc_34_12    TACAACAGG-AGCTCAACCAACTTCACGAAACGTTTACCCATCTGGGCCAGAATCATCTACACCAGGAGGACAAGTAGCATCGCATAAC---TCCGGCAGCACCTTCTACTCCACAGCCTGGATGTCA
Minc_34_3     TACAACAGG-AGCTCAACCAACTTCACGAAACGTTTACCCATCTGGGCCAGAATCATCTACACCAGGAGGACAAGTAGCATCGCATAA--C-TCCGGCAGCACCTTCTACTCCACAGCCTGGATGTCA
Minc_34_5     TACAACAGG-AGCTCAACCAACTTCACGAAACGTTTACCCATCTGGGCCAGAATCATCTACACCAGGAGGACAAGTAGCATCGCATAAC---TCCGGCAGCACCTTCTACTCCACAGCCTGGATGTCA
Minc_34_7     TACAACAGG-AGCTCAACCAACTTCACGAAACGTTTACCCATCTGGGCCAGAATCATCTACACCAGGAGGACAAGTAGCATCGCATAAC--CTCCGGCAGCACCTTCTACTCCACAGCCTGGATGTCA
Minc_34_9     TACAACAGG-AGCTCAACCAACTTCACGAAACGTTTACCCATCTGGGCCAGAATCATCTACACCAGGAGGACAAGTAGCATCGCATAAC---TCCGGCAGCACCTTCTACTCCACAGCCTGGATGTCA
Minc_37_10    TACAACAGG-AGCTCAACCAACTTCACGAAACGTTTACCCATCTGGGCCAGAATCATCTACACCAGGAGGACAAGTAGCATCGCATAAG-CCTCCGGCAGCACCTTCTACTCCACAGCCTGGATGTCA
Minc_37_12    TACAACAGG-AGCTCAACCAACTTCACGAAACGTTTACCCATCTGGGCCAGAATCATCTACACCAGGAGGACAAGTAGCATCGCATAAG-CCTCCGGCAGCACCTTCTACTCCACAGCCTGGATGTCA
Minc_37_2     TACAACAGG-AGCTCAACCAACTTCACGAAACGTTTACCCATCTGGGCCAGAATCATCTACACCAGGAGGACAAGTAGCATCGCATAAG-CCTCCGGCAGCACCTTCTACTCCACAGCCTGGATGTCA
Minc_37_3     TACAACAGG-AGCTCAACCAACTTCACGAAACGTTTACCCATCTGGGCCAGAATCATCTACACCAGGAGGACAAGTAGCATCGCATAAG-CCTCCGGCAGCACCTTCTACTCCACAGCCTGGATGTCA
Minc_37_4     TACAACAGG-AGCTCAACCAACTTCACGAAACGTTTACCCATCTGGGCCAGAATCATCTACACCAGGAGGACAAGTAGCATCGCATAAG-CCTCCGGCAGCACCTTCTACTCCACAGCCTGGATGTCA
Minc_37_5     TACAACAGG-AGCTCAACCAACTTCACGAAACGTTTACCCATCTGGGCCAGAATCATCTACACCAGGAGGACAAGTAGCATCGCATAAG-CCTCCGGCAGCACCTTCTACTCCACAGCCTGGATGTCA
Minc_37_6     TACAACAGG-AGCTCAACCAACTTCACGAAACGTTTACCCATCTGGGCCAGAATCATCTACACCAGGAGGACAAGTAGCATCGCATAAG-CCTCCGGCAGCACCTTCTACTCCACAGCCTGGATGTCA
Minc_37_8     TACAACAGG-AGCTCAACCAACTTCACGAAACGTTTACCCATCTGGGCCAGAATCATCTACACCAGGAGGACAAGTAGCATCGCATAAG-CCTCCGGCAGCACCTTCTACTCCACAGCCTGGATGTCA
Minc_37_9     TACAACAGG-AGCTCAACCAACTTCACGAAACGTTTACCCATCTGGGCCAGAATCATCTACACCAGGAGGACAAGTAGCATCGCATAAG-CCTCCGGCAGCACCTTCTACTCCACAGCCTGGATGTCA
Minc_42_1     TACAACAGG-AGCTCAACCAACTTCACGAAACGTTTACCCATCTGGGCCAGAATCATCTACACCAGGAGGACAAGTAGCATCGCATAAC---TCCGGCAGCACCTTCTACTCCACAGCCTGGATGTCA
Minc_42_10    TACAACAGG-AGCTCAACCAACTTCACGAAACGTTTACCCATCTGGGCCAGAATCATCTACACCAGGAGGACAAGTAGCATCGCATAAC---TCCGGCAGCACCTTCTACTCCACAGCCTGGATGTCA
Minc_42_11    TACAACAGG-AGCTCAACCAACTTCACGAAACGTTTACCCATCTGGGCCAGAATCATCTACACCAGGAGGACAAGTAGCATCGCATAA---CTCCGGCAGCACCTTCTACTCCACAGCCTGGATGTCA
Minc_42_12    TACAACAGG-AGCTCAACCAACTTCACGAAACGTTTACCCATCTGGGCCAGAATCATCTACACCAGGAGGACAAGTAGCATCGCATAA---CTCCGGCAGCACCTTCTACTCCACAGCCTGGATGTCA
Minc_42_3     TACAACAGG-AGCTCAACCAACTTCACGAAACGTTTACCCATCTGGGCCAGAATCATCTACACCAGGAGGACAAGTAGCATCGCATAA---CTCCGGCAGCACCTTCTACTCCACAGCCTGGATGTCA
Minc_42_4     TACAACAGG-AGCTCAACCAACTTCACGAAACGTTTACCCATCTGGGCCAGAATCATCTACACCAGGAGGACAAGTAGCATCGCATAA---CTCCGGCAGCACCTTCTACTCCACAGCCTGGATGTCA
Minc_42_5     TACAACAGG-AGCTCAACCAACTTCACGAAACGTTTACCCATCTGGGCCAGAATCATCTACACCAGGAGGACAAGTAGCATCGCATAAC---TCCGGCAGCACCTTCTACTCCACAGCCTGGATGTCA
Minc_42_7     TACAACAGG-AGCTCAACCAACTTCACGAAACGTTTACCCATCTGGGCCAGAATCATCTACACCAGGAGGACAAGTAGCATCGCATAAC---TCCGGCAGCACCTTCTACTCCACAGCCTGGATGTCA
Minc_42_8     TACAACAGG-AGCTCAACCAACTTCACGAAACGTTTACCCATCTGGGCCAGAATCATCTACACCAGGAGGACAAGTAGCATCGCATAAC---TCCGGCAGCACCTTCTACTCCACAGCCTGGATGTCA
Minc_42_9     TACAACAGG-AGCTCAACCAACTTCACGAAACGTTTACCCATCTGGGCCAGAATCATCTACACCAGGAGGACAAGTAGCATCGCATAA---CTCCGGCAGCACCTTCTACTCCACAGCCTGGATGTCA
Minc_45_10    TACAACAGG-AGCTCAACCAACTTCACGAAACGTTTACCCATCTGGGCCAGAATCATCTACACCAGGAGGACAAGTAGCATCGCATAAG-CCTCCGGCAGCACCTTCTACTCCACAGCCTGGATGTCA
Minc_45_11    TACAACAGG-AGCTCAACCAACTTCACGAAACGTTTACCCATCTGGGCCAGAATCATCTACACCAGGAGGACAAGTAGCATCGCATAAG-CCTCCGGCAGCACCTTCTACTCCACAGCCTGGATGTCA
Minc_45_12    TACAACAGG-AGCTCAACCAACTTCACGAAACGTTTACCCATCTGGGCCAGAATCATCTACACCAGGAGGACAAGTAGCATCGCATAAG-CCTCCGGCAGCACCTTCTACTCCACAGCCTGGATGTCA
Minc_45_13    TACAACAGG-AGCTCAACCAACTTCACGAAACGTTTACCCATCTGGGCCAGAATCATCTACACCAGGAGGACAAGTAGCATCGCATAAGCC-TCCGGCAGCACCTTCTACTCCACAGCCTGGATGTCA
Minc_45_7     TACAACAGG-AGCTCAACCAACTTCACGAAACGTTTACCCATCTGGGCCAGAATCATCTACACCAGGAGGACAAGTAGCATCGCATAAG-CCTCCGGCAGCACCTTCTACTCCACAGCCTGGATGTCA
Minc_45_9     TACAACAGG-AGCTCAACCAACTTCACGAAACGTTTACCCATCTGGGCCAGAATCATCTACACCAGGAGGACAAGTAGCATCGCATAAG-CCTCCGGCAGCACCTTCTACTCCACAGCCTGGATGTCA
Minc_47_1     TACAACAGG-AGCTCAACCAACTTCACGAAACGTTTACCCATCTGGGCCAGAATCATCTACACCAGGAGGACAAGTAGCATCGCATAA---CTCCGGCAGCACCTTCTACTCCACAGCCTGGATGTCA
Minc_47_10    TACAACAGG-AGCTCAACCAACTTCACGAAACGTTTACCCATCTGGGCCAGAATCATCTACACCAGGAGGACAAGTAGCATCGCATAAG-CCTCCGGCAGCACCTTCTACTCCACAGCCTGGATGTCA
Minc_47_12    TACAACAGG-AGCTCAACCAACTTCACGAAACGTTTACCCATCTGGGCCAGAATCATCTACACCAGGAGGACAAGTAGCATCGCATAA---CTCCGGCAGCACCTTCTACTCCACAGCCTGGATGTCA
Minc_47_13    TACAACAGG-AGCTCAACCAACTTCACGAAACGTTTACCCATCTGGGCCAGAATCATCTACACCAGGAGGACAAGTAGCATCGCATAAG-CCTCCGGCAGCACCTTCTACTCCACAGCCTGGATGTCA
Minc_47_3     TACAACAGG-AGCTCAACCAACTTCACGAAACGTTTACCCATCTGGGCCAGAATCATCTACACCAGGAGGACAAGTAGCATCGCATAAG-CCTCCGGCAGCACCTTCTACTCCACAGCCTGGATGTCA
Minc_47_7     TACAACAGG-AGCTCAACCAACTTCACGAAACGTTTACCCATCTGGGCCAGAATCATCTACACCAGGAGGACAAGTAGCATCGCATAAG-CCTCCGGCAGCACCTTCTACTCCACAGCCTGGATGTCA
Minc_47_8     TACAACAGG-AGCTCAACCAACTTCACGAAACGTTTACCCATCTGGGCCAGAATCATCTACACCAGGAGGACAAGTAGCATCGCATAAG-CCTCCGGCAGCACCTTCTACTCCACAGCCTGGATGTCA
Minc_51_10    TACAACAGG-AGCTCAACCAACTTCACGAAACGTTTACCCATCTGGGCCAGAATCATCTACACCAGGAGGACAAGTAGCATCGCATAAG-CCTCCGGCAGCACCTTCTACTCCACAGCCTGGATGTCA
Minc_51_11    TACAACAGG-AGCTCAACCAACTTCACGAAACGTTTACCCATCTGGGCCAGAATCATCTACACCAGGAGGACAAGTAGCATCGCATAAG-CCTCCGGCAGCACCTTCTACTCCACAGCCTGGATGTCA
Minc_51_4     TACAACAGG-AGCTCAACCAACTTCACGAAACGTTTACCCATCTGGGCCAGAATCATCTACACCAGGAGGACAAGTAGCATCGCATAAG-CCTCCGGCAGCACCTTCTACTCCACAGCCTGGATGTCA
Minc_51_6     TACAACAGG-AGCTCAACCAACTTCACGAAACGTTTACCCATCTGGGCCAGAATCATCTACACCAGGAGGACAAGTAGCATCGCATAAG-CCTCCGGCAGCACCTTCTACTCCACAGCCTGGATGTCA
Minc_51_7     TACAACAGG-AGCTCAACCAACTTCACGAAACGTTTACCCATCTGGGCCAGAATCATCTACACCAGGAGGACAAGTAGCATCGCATAA--C-TCCGGCAGCACCTTCTACTCCACAGCCTGGATGTCA
Minc_54_1     TACAACAGG-AGCTCAACCAACTTCACGAAACGTTTACCCATCTGGGCCAGAATCATCTACACCAGGAGGACAAGTAGCATCGCATAAG-CCTCCGGCAGCACCTTCTACTCCACAGCCTGGATGTCA
Minc_54_10    TACAACAGG-AGCTCAACCAACTTCACGAAACGTTTACCCATCTGGGCCAGAATCATCTACACCAGGAGGACAAGTAGCATCGCATAAG-CCTCCGGCAGCACCTTCTACTCCACAGCCTGGATGTCA
Minc_54_3     TACAACAGG-AGCTCAACCAACTTCACGAAACGTTTACCCATCTGGGCCAGAATCATCTACACCAGGAGGACAAGTAGCATCGCATAAG-CCTCCGGCAGCACCTTCTACTCCACAGCCTGGATGTCA
Minc_54_4     TACAACAGG-AGCTCAACCAACTTCACGAAACGTTTACCCATCTGGGCCAGAATCATCTACACCAGGAGGACAAGTAGCATCGCATAAG-CCTCCGGCAGCACCTTCTACTCCACAGCCTGGATGTCA
Minc_54_5     TACAACAGG-AGCTCAACCAACTTCACGAAACGTTTACCCATCTGGGCCAGAATCATCTACACCAGGAGGACAAGTAGCATCGCATAAGCC-TCCGGCAGCACCTTCTACTCCACAGCCTGGATGTCA
Minc_54_6     TACAACAGG-AGCTCAACCAACTTCACGAAACGTTTACCCATCTGGGCCAGAATCATCTACACCAGGAGGACAAGTAGCATCGCATAAG-CCTCCGGCAGCACCTTCTACTCCACAGCCTGGATGTCA
Minc_56_1     TACAACAGG-AGCTCAACCAACTTCACGAAACGTTTACCCATCTGGGCCAGAATCATCTACACCAGGAGGACAAGTAGCATCGCATAAG-CCTCCGGCAGCACCTTCTACTCCACAGCCTGGATGTCA
Minc_56_12    TACAACAGG-AGCTCAACCAACTTCACGAAACGTTTACCCATCTGGGCCAGAATCATCTACACCAGGAGGACAAGTAGCATCGCATAAG-CCTCCGGCAGCACCTTCTACTCCACAGCCTGGATGTCA
Minc_56_2     TACAACAGG-AGCTCAACCAACTTCACGAAACGTTTACCCATCTGGGCCAGAATCATCTACACCAGGAGGACAAGTAGCATCGCATAAGCC-TCCGGCAGCACCTTCTACTCCACAGCCTGGATGTCA
Minc_56_3     TACAACAGG-AGCTCAACCAACTTCACGAAACGTTTACCCATCTGGGCCAGAATCATCTACACCAGGAGGACAAGTAGCATCGCATAA---CTCCGGCAGCACCTTCTACTCCACAGCCTGGATGTCA
Minc_56_4     TACAACAGG-AGCTCAACCAACTTCACGAAACGTTTACCCATCTGGGCCAGAATCATCTACACCAGGAGGACAAGTAGCATCGCATAA---CTCCGGCAGCACCTTCTACTCCACAGCCTGGATGTCA
Minc_56_5     TACAACAGG-AGCTCAACCAACTTCACGAAACGTTTACCCATCTGGGCCAGAATCATCTACACCAGGAGGACAAGTAGCATCGCATAAC---GCCGGCAGCACCTTCTACTCCACAGCCTGGATGTCA
Minc_56_6     TACAACAGG-AGCTCAACCAACTTCACGAAACGTTTACCCATCTGGGCCAGAATCATCTACACCAGGAGGACAAGTAGCATCGCATAAC---TCCGGCAGCACCTTCTACTCCACAGCCTGGATGTCA
Minc_56_7     TACAACAGG-AGCTCAACCAACTTCACGAAACGTTTACCCATCTGGGCCAGAATCATCTACACCAGGAGGACAAGTAGCATCGCATAAC---TCCGGCAGCACCTTCTACTCCACAGCCTGGATGTCA
Minc_58_1     TACAACAGG-AGCTCAACCAACTTCACGAAACGTTTACCCATCTGGGCCAGAATCATCTACACCAGGAGGACAAGTAGCATCGCATAAG-CCTCCGGCAGCACCTTCTACTCCACAGCCTGGATGTCA
Minc_58_13    TACAACAGG-AGCTCAACCAACTTCACGAAACGTTTACCCATCTGGGCCAGAATCATCTACACCAGGAGGACAAGTAGCATCGCATAAG-CCTCCGGCAGCACCTTCTACTCCACAGCCTGGATGTCA
Minc_58_2     TACAACAGG-AGCTCAACCAACTTCACGAAACGTTTACCCATCTGGGCCAGAATCATCTACACCAGGAGGACAAGTAGCATCGCATAAG-CCTCCGGCAGCACCTTCTACTCCACAGCCTGGATGTCA
Minc_58_3     TACAACAGG-AGCTCAACCAACTTCACGAAACGTTTACCCATCTGGGCCAGAATCATCTACACCAGGAGGACAAGTAGCATCGCATAAG-CCTCCGGCAGCACCTTCTACTCCACAGCCTGGATGTCA
Minc_58_4     TACAACAGG-AGCTCAACCAACTTCACGAAACGTTTACCCATCTGGGCCAGAATCATCTACACCAGGAGGACAAGTAGCATCGCATAAG-CCTCCGGCAGCACCTTCTACTCCACAGCCTGGATGTCA
Minc_58_5     TACAACAGGTAGCTCAACCAACTTCACGAAACGTTTACCCATCTGGGCCAGAATCATCTACACCAGGAGGACAAGTAGCATCGCATAAG-CCTCCGGCAGCACCTTCTACTCCACAGCCTGGATGTCA
Minc_58_6     TACAACAGG-AGCTCAACCAACTTCACGAAACGTTTACCCATCTGGGCCAGAATCATCTACACCAGGAGGACAAGTAGCATCGCATAA---CTCCGGCAGCACCTTCTACTCCACAGCCTGGATGTCA
Minc_58_7     TACAACAGG-AGCTCAACCAACTTCACGAAACGTTTACCCATCTGGGCCAGAATCATCTACACCAGGAGGACAAGTAGCATCGCATAAG-CCTCCGGCAGCACCTTCTACTCCACAGCCTGGATGTCA
Minc_58_8     TACAACAGG-AGCTCAACCAACTTCACGAAACGTTTACCCATCTGGGCCAGAATCATCTACACCAGGAGGACAAGTAGCATCGCATAAG-CCTCCGGCAGCACCTTCTACTCCACAGCCTGGATGTCA
Minc_58_9     TACAACAGG-AGCTCAACCAACTTCACGAAACGTTTACCCATCTGGGCCAGAATCATCTACACCAGGAGGACAAGTAGCATCGCATAAG-CCTCCGGCAGCACCTTCTACTCCACAGCCTGGATGTCA
Minc_65_1     TACAACAGG-AGCTCAACCAACTTCACGAAACGTTTACCCATCTGGGCCAGAATCATCTACACCAGGAGGACAAGTAGCATCGCATAAG-CCTCCGGCAGCACCTTCTACTCCACAGCCTGGATGTCA
Minc_65_11    TACAACAGG-AGCTCAACCAACTTCACGAAACGTTTACCCATCTGGGCCAGAATCATCTACACCAGGAGGACAAGTAGCATCGCATAAG-CCTCCGGCAGCACCTTCTACTCCACAGCCTGGATGTCA
Minc_65_3     TACAACAGG-AGCTCAACCAACTTCACGAAACGTTTACCCATCTGGGCCAGAATCATCTACACCAGGAGGACAAGTAGCATCGCATAAG---TCCGGCAGCACCTTCTACTCCACAGCCTGGATGTCA
Minc_65_6     TACAACAGG-AGCTCAACCAACTTCACGAAACGTTTACCCATCTGGGCCAGAATCATCTACACCAGGAGGACAAGTAGCATCGCATAAG-CCTCCGGCAGCACCTTCTACTCCACAGCCTGGATGTCA
Minc_65_8     TACAACAGG-AGCTCAACCAACTTCACGAAACGTTTACCCATCTGGGCCAGAATCATCTACACCAGGAGGACAAGTAGCATCGCATAAG-CCTCCGGCAGCACCTTCTACTCCACAGCCTGGATGTCA
Minc_65_9     TACAACAGG-AGCTCAACCAACTTCACGAAACGTTTACCCATCTGGGCCAGAATCATCTACACCAGGAGGACAAGTAGCATCGCATAAG-CCTCCGGCAGCACCTTCTACTCCACAGCCTGGATGTCA
Minc_76_1     TACAACAGG-AGCTCAACCAACTTCACGAAACGTTTACCCATCTGGGCCAGAATCATCTACACCAGGAGGACAAGTAGCATCGCATAAG-CCTCCGGCAGCACCTTCTACTCCACAGCCTGGATGTCA
Minc_76_10    TACAACAGG-AGCTCAACCAACTTCACGAAACGTTTACCCATCTGGGCCAGAATCATCTACACCAGGAGGACAAGTAGCATCGCATAAG-CCTCCGGCAGCACCTTCTACTCCACAGCCTGGATGTCA
```

```
Minc_76_12    TACAACAGG-AGCTCA-CCAACTTCACGAAACGTTTACCCATCTGGGCCAGAATCATCTACACCAGGAGGACAAGTAGCATCGCATAAG-CCTCCGGCAGCACCTTCTACTCCACAGCCTGGATGTCA
Minc_76_13    TACAACAGG-AGCTCAACCAACTTCACGAAACGTTTACCCATCTGGGCCAGAATCATCTACACCAGGAGGACAAGTAGCATCGCATAAG-CCTCCGGCAGCACCTTCTACTCCACAGCCTGGATGTCA
Minc_76_14    TACAACAGG-AGCTCAACCAACTTCACGAAACGTTTACCCATCTGGGCCAGAATCATCTACACCAGGAGGACAAGTAGCATCGCATAAG-CCTCCGGCAGCACCTTCTACTCCACAGCCTGGATGTCA
Minc_76_3     TACAACAGG-AGCTCAACCAACTTCACGAAACGTTTACCCATCTGGGCCAGAATCATCTACACCAGGAGGACAAGTAGCATCGCATAAG-CCTCCGGCAGCACCTTCTACTCCACAGCCTGGATGTCA
Minc_76_7     TACAACAGG-AGCTCAACCAACTTCACGAAACGTTTACCCATCTGGGCCAGAATCATCTACACCAGGAGGACAAGTAGCATCGCATAA---CTCCGGCAGCACCTTCTACTCCACAGCCTGGATGTCA
Minc_76_8     TACAACAGG-AGCTCAACCAACTTCACGAAACGTTTACCCATCTGGGCCAGAATCATCTACACCAGGAGGACAAGTAGCATCGCATAAG-CCTCCGGCAGCACCTTCTACTCCACAGCCTGGATGTCA
Minc_76_9     TACAACAGG-AGCTCAACCAACTTCACGAAACGTTTACCCATCTGGGCCAGAATCATCTACACCAGGAGGACAAGTAGCATCGCATAAG-CCTCCGGCAGCACCTTCTACTCCACAGCCTGGATGTCA
Minc_78_1     TACAACAGG-AGCTCAACCAACTTCACGAAACGTTTACCCATCTGGGCCAGAATCATCTACACCAGGAGGACAAGTAGCATCGCATAA---CTCCGGCAGCACCTTCTACTCCACAGCCTGGATGTCA
Minc_78_10    TACAACAGG-AGCTCAACCAACTTCACGAAACGTTTACCCATCTGGGCCAGAATCATCTACACCAGGAGGACAAGTAGCATCGCATAAG-CCTCCGGCAGCACCTTCTACTCCACAGCCTGGATGTCA
Minc_78_11    TACAACAGG-AGCTCAACCAACTTCACGAAACGTTTACCCATCTGGGCCAGAATCATCTACACCAGGAGGACAAGTAGCATCGCATAAG-CCTCCGGCAGCACCTTCTACTCCACAGCCTGGATGTCA
Minc_78_13    TACAACAGG-AGCTCAACCAACTTCACGAAACGTTTACCCATCTGGGCCAGAATCATCTACACCAGGAGGACAAGTAGCATCGCATAAG-CCTCCGGCAGCACCTTCTACTCCACAGCCTGGATGTCA
Minc_78_15    TACAACAGG-AGCTCAACCAACTTCACGAAACGTTTACCCATCTGGGCCAGAATCATCTACACCAGGAGGACAAGTAGCATCGCATAA--C-TCCGGCAGCACCTTCTACTCCACAGCCTGGATGTCA
Minc_78_16    TACAACAGG-AGCTCAACCAACTTCACGAAACGTTTACCCATCTGGGCCAGAATCATCTACACCAGGAGGACAAGTAGCATCGCATAA--C-TCCGGCAGCACCTTCTACTCCACAGCCTGGATGTCA
Minc_78_17    TACAACAGG-AGCTCAACCAACTTCACGAAACGTTTACCCATCTGGGCCAGAATCATCTACACCAGGAGGACAAGTAGCATCGCATAA---CTCCGGCAGCACCTTCTACTCCACAGCCTGGATGTCA
Minc_78_7     TACAACAGG-AGCTCAACCAACTTCACGAAACGTTTACCCATCTGGGCCAGAATCATCTACACCAGGAGGACAAGTAGCATCGCATAAG-CCTCCGGCAGCACCTTCTACTCCACAGCCTGGATGTCA
Minc_81_12    TACAACAGG-AGCTCAACCAACTTCACGAAACGTTTACCCATCTGGGCCAGAATCATCTACACCAGGAGGACAAGTAGCATCGCATAAG-CCTCCGGCAGCACCTTCTACTCCACAGCCTGGATGTCA
Minc_81_13    TACAACAGG-AGCTCAACCAACTTCACGAAACGTTTACCCATCTGGGCCAGAATCATCTACACCAGGAGGACAAGTAGCATCGCATAA--C-TCCGGCAGCACCTTCTACTCCACAGCCTGGATGTCA
Minc_81_14    TACAACAGG-AGCTCAACCAACTTCACGAAACGTTTACCCATCTGGGCCAGAATCATCTACACCAGGAGGACAAGTAGCATCGCATAAG-CCTCCGGCAGCACCTTCTACTCCACAGCCTGGATGTCA
Minc_81_15    TACAACAGG-AGCTCAACCAACTTCACGAAACGTTTACCCATCTGGGCCAGAATCATCTACACCAGGAGGACAAGTAGCATC-CATAAG-CCTCCGG-AGCACCTTCTACTCCACAGCCTGGATGTCA
Minc_81_2     TACAACAGG-AGCTCAACCAACTTCACGAAACGTTTACCCATCTGGGCCAGAATCATCTACACCAGGAGGACAAGTAGCATCGCATAAG-CCTCCGGCAGCACCTTCTACTCCACAGCCTGGATGTCA
Minc_81_4     TACAACAGG-AGCTCAACCAACTTCACGAAACGTTTACCCATCTGGGCCAGAATCATCTACACCAGGAGGACAAGTAGCATCGCATAAG-CCTCCGGCAGCACCTTCTACTCCACAGCCTGGATGTCA
Minc_81_6     TACAACAGG-AGCTCAACCAACTTCACGAAACGTTTACCCATCTGGGCCAGAATCATCTACACCAGGAGGACAAGTAGCATCGCATAAG-CCTCCGGCAGCACCTTCTACTCCACAGCCTGGATGTCA
Minc_81_8     TACAACAGG-AGCTCAACCAACTTCACGAAACGTTTACCCATCTGGGCCAGAATCATCTACACCAGGAGGACAAGTAGCATCGCATAAG-CCTCCGGCAGCACCTTCTACTCCACAGCCTGGATGTCA
              ********* ****** ***************************************************************** ****      **** ******************************
```

```
                                                                                                                           ▼
              TCAACCACCAGCAGCTCCAACAACAAAACCTCCACGAAATTTATATCCATCTGGACCAGAACCTACTCCAAAACAACCTGGATATCCCCAAGGACCAGCATCTACTCCAAAACAACCTGGATATCCTC
Minc_18_1     TCAACCACCAGCAGCTCCAACAACAAAACCTCCACGAAATTTATATCCATCTGGACCAGAACCTACTCCAAAACAACCTGGATATCCCCAAGGACCAGCATCTACTCCAAAACAACCTGGATATCCTC
Minc_18_10    TCAACCACCAGCAGCTCCAACAACAAAACCTCCACGAAATTTATATCCATCTGGACCAGAACCTACTCCAAAACAACCTGGATATCCCCAAGGACCAGCATCTACTCCAAAACAACCTGGATATCCTC
Minc_18_11    TCAACCACCAGCAGCTCCAACAACAAAACCTCCACGAAATTTATATCCATCTGGACCAGAACCTACTCCAAAACAACCTGGATATCCCCAAGGACCAGCATCTACTCCAAAACAACCTGGATATCCTC
Minc_18_13    TCAACCACCAGCAGCTCCAACAACAAAACCTCCACGAAATTTATATCCATCTGGACCAGAACCTACTCCAAAACAACCTGGATATCCCCAAGGACCAGCATCTACTCCAAAACAACCTGGATATCCTC
Minc_18_3     TCAACCACCAGCAGCTCCAACAACAAAACCTCCACGAAATTTATATCCATCTGGACCAGAACCTACTCCAAAACAACCTGGATATCCCCAAGGACCAGCATCTACTCCAAAACAACCTGGATATCCTC
Minc_18_4     TCAACCACCAGCAGCTCCAACAACAAAACCTCCACGAAATTTATATCCATCTGGACCAGAACCTACTCCAAAACAACCTGGATATCCCCAAGGACCAGCATCTACTCCAAAACAACCTGGATATCCTC
Minc_18_5     TCAACCACCAGCAGCTCCAACAACAAAACCTCCACGAAATTTATATCCATCTGGACCAGAACCTACTCCAAAACAACCTGGATATCCCCAAGGACCAGCATCTACTCCAAAACAACCTGGATATCCTC
Minc_18_7     TCAACCACCAGCAGCTCCAACAACAAAACCTCCACGAAATTTATATCCATCTGGACCAGAACCTACTCCAAAACAACCTGGATATCCCCAAGGACCAGCATCTACTCCAAAACAACCTGGATATCCTC
Minc_18_8     TCAACCACCAGCAGCTCCAACAACAAAACCTCCACGAAATTTATATCCATCTGGACCAGAACCTACTCCAAAACAACCTGGATATCCCCAAGGACCAGCATCTACTCCAAAACAACCTGGATATCCTC
Minc_18_9     TCAACCACCAGCAGCTCCAACAACAAAACCTCCACGAAATTTATATCCATCTGGACCAGAACCTACTCCAAAACAACCTGGATATCCCCAAGGACCAGCATCTACTCCAAAACAACCTGGATATCCTC
Minc_22_14    TCAACCACCAGCAGCTCCAACAACAAAACCTCCACGAAATTTATATCCATCTGGACCAGAACCTACTCCAAAACAACCTGGATATCCCCAAGGACCAGCATCTACTCCAAAACAACCTGGATATCCTC
Minc_22_15    TCAACCACCAGCAGCTCCAACAACAAAACCTCCACGAAATTTATATCCATCTGGACCAGAACCTACTCCAAAACAACCTGGATATCCCCAAGGACCAGCATCTACTCCAAAACAACCTGGATATCCTC
Minc_22_2     TCAACCACCAGCAGCTCCAACAACAAAACCTCCACGAAATTTATATCCATCTGGACCAGAACCTACTCCAAAACAACCTGGATATCCCCAAGGACCAGCATCTACTCCAAAACAACCTGGATATCCTC
Minc_22_3     TCAACCACCAGCAGCTCCAACAACAAAACCTCCACGAAATTTATATCCATCTGGACCAGAACCTACTCCAAAACAACCTGGATATCCCCAAGGACCAGCATCTACTCCAAAACAACCTGGATATCCTC
Minc_22_5     TCAACCACCAGCAGCTCCAACAACAAAACCTCCACGAAATTTATATCCATCTGGACCAGAACCTACTCCAAAACAACCTGGATATCCCCAAGGACCAGCATCTACTCCAAAACAACCTGGATATCCTC
Minc_22_7     TCAACCACCAGCAGCTCCAACAACAAAACCTCCACGAAATTTATATCCATCTGGACCAGAACCTACTCCAAAACAACCTGGATATCCCCAAGGACCAGCATCTACTCCAAAACAACCTGGATATCCTC
Minc_22_8     TCAACCACCAGCAGCTCCAACAACAAAACCTCCACGAAATTTATATCCATCTGGACCAGAACCTACTCCAAAACAACCTGGATATCCCCAAGGACCAGCATCTACTCCAAAACAACCTGGATATCCTC
Minc_30_1     TCAACCACCAGCAGCTCCAACAACAAAACCTCCACGAAATTTATATCCATCTGGACCAGAACCTACTCCAAAACAACCTGGATATCCCCAAGGACCAGCATCTACTCCAAAACAACCTGGATATCCTC
Minc_30_10    TCAACCACCAGCAGCTCCAACAACAAAACCTCCACGAAATTTATATCCATCTGGACCAGAACCTACTCCAAAACAACCTGGATATCCCCAAGGACCAGCATCTACTCCAAAACAACCTGGATATCCTC
Minc_30_11    TCAACCACCAGCAGCTCCAACAACAAAACCTCCACGAAATTTATATCCATCTGGACCAGAACCTACTCCAAAACAACCTGGATATCCCCAAGGACCAGCATCTACTCCAAAACAACCTGGATATCCTC
Minc_30_4     TCAACCACCAGCAGCTCCAACAACAAAACCTCCACGAAATTTATATCCATCTGGACCAGAACCTACTCCAAAACAACCTGGATATCCCCAAGGACCAGCATCTACTCCAAAACAACCTGGATATCCTC
Minc_30_5     TCAACCACCAGCAGCTCCAACAACAAAACCTCCACGAAATTTATATCCATCTGGACCAGAACCTACTCCAAAACAACCTGGATATCCCCAAGGACCAGCATCTACTCCAAAACAACCTGGATATCCTC
Minc_34_1     TCAACCACCAGCAGCTCCAACAACAAAACCTCCACGAAATTTATATCCATCTGGACCAGAACCTACTCCAAAACAACCTGGATATCCCCAAGGACCAGCATCTACTCCAAAACAACCTGGATATCCTC
Minc_34_10    TCAACCACCAGCAGCTCCAACAACAAAACCTCCACGAAATTTATATCCATCTGGACCAGAACCTACTCCAAAACAACCTGGATATCCCCAAGGACCAGCATCTACTCCAAAACAACCTGGATATCCTC
Minc_34_11    TCAACCACCAGCAGCTCCAACAACAAAACCTCCACGAAATTTATATCCATCTGGACCAGAACCTACTCCAAAACAACCTGGATATCCCCAAGGACCAGCATCTACTCCAAAACAACCTGGATATCCTC
Minc_34_12    TCAACCACCAGCAGCTCCAACAACAAAACCTCCACGAAATTTATATCCATCTGGACCAGAACCTACTCCAAAACAACCTGGATATCCCCAAGGACCAGCATCTACTCCAAAACAACCTGGATATCCTC
Minc_34_3     TCAACCACCAGCAGCTCCAACAACAAAACCTCCACGAAATTTATATCCATCTGGACCAGAACCTACTCCAAAACAACCTGGATATCCCCAAGGACCAGCATCTACTCCAAAACAACCTGGATATCCTC
Minc_34_5     TCAACCACCAGCAGCTCCAACAACAAAACCTCCACGAAATTTATATCCATCTGGACCAGAACCTACTCCAAAACAACCTGGATATCCCCAAGGACCAGCATCTACTCCAAAACAACCTGGATATCCTC
Minc_34_7     TCAACCACCAGCAGCTCCAACAACAAAACCTCCACGAAATTTATATCCATCTGGACCAGAACCTACTCCAAAACAACCTGGATATCCCCAAGGACCAGCATCTACTCCAAAACAACCTGGATATCCTC
Minc_34_9     TCAACCACCAGCAGCTCCAACAACAAAACCTCCACGAAATTTATATCCATCTGGACCAGAACCTACTCCAAAACAACCTGGATATCCCCAAGGACCAGCATCTACTCCAAAACAACCTGGATATCCTC
Minc_37_10    TCAACCACCAGCAGCTCCAACAACAAAACCTCCACGAAATTTATATCCATCTGGACCAGAACCTACTCCAAAACAACCTGGATATCCCCAAGGACCAGCATCTACTCCAAAACAACCTGGATATCCTC
Minc_37_12    TCAACCACCAGCAGCTCCAACAACAAAACCTCCACGAAATTTATATCCATCTGGACCAGAACCTACTCCAAAACAACCTGGATATCCCCAAGGACCAGCATCTACTCCAAAACAACCTGGATATCCTC
Minc_37_2     TCAACCACCAGCAGCTCCAACAACAAAACCTCCACGAAATTTATATCCATCTGGACCAGAACCTACTCCAAAACAACCTGGATATCCCCAAGGACCAGCATCTACTCCAAAACAACCTGGATATCCTC
Minc_37_3     TCAACCACCAGCAGCTCCAACAACAAAACCTCCACGAAATTTATATCCATCTGGACCAGAACCTACTCCAAAACAACCTGGATATCCCCAAGGACCAGCATCTACTCCAAAACAACCTGGATATCCTC
Minc_37_4     TCAACCACCAGCAGCTCCAACAACAAAACCTCCACGAAATTTATATCCATCTGGACCAGAACCTACTCCAAAACAACCTGGATATCCCCAAGGACCAGCATCTACTCCAAAACAACCTGGATATCCTC
Minc_37_5     TCAACCACCAGCAGCTCCAACAACAAAACCTCCACGAAATTTATATCCATCTGGACCAGAACCTACTCCAAAACAACCTGGATATCCCCAAGGACCAGCATCTACTCCAAAACAACCTGGATATCCTC
Minc_37_6     TCAACCACCAGCAGCTCCAACAACAAAACCTCCACGAAATTTATATCCATCTGGACCAGAACCTACTCCAAAACAACCTGGATATCCCCAAGGACCAGCATCTACTCCAAAACAACCTGGATATCCTC
Minc_37_8     TCAACCACCAGCAGCTCCAACAACAAAACCTCCACGAAATTTATATCCATCTGGACCAGAACCTACTCCAAAACAACCTGGATATCCCCAAGGACCAGCATCTACTCCAAAACAACCTGGATATCCTC
Minc_37_9     TCAACCACCAGCAGCTCCAACAACAAAACCTCCACGAAATTTATATCCATCTGGACCAGAACCTACTCCAAAACAACCTGGATATCCCCAAGGACCAGCATCTACTCCAAAACAACCTGGATATCCTC
Minc_42_1     TCAACCACCAGCAGCTCCAACAACAAAACCTCCACGAAATTTATATCCATCTGGACCAGAACCTACTCCAAAACAACCTGGATATCCCCAAGGACCAGCATCTACTCCAAAACAACCTGGATATCCTC
Minc_42_10    TCAACCACCAGCAGCTCCAACAACAAAACCTCCACGAAATTTATATCCATCTGGACCAGAACCTACTCCAAAACAACCTGGATATCCCCAAGGACCAGCATCTACTCCAAAACAACCTGGATATCCTC
Minc_42_11    TCAACCACCAGCAGCTCCAACAACAAAACCTCCACGAAATTTATATCCATCTGGACCAGAACCTACTCCAAAACAACCTGGATATCCCCAAGGACCAGCATCTACTCCAAAACAACCTGGATATCCTC
Minc_42_12    TCAACCACCAGCAGCTCCAACAACAAAACCTCCACGAAATTTATATCCATCTGGACCAGAACCTACTCCAAAACAACCTGGATATCCCCAAGGACCAGCATCTACTCCAAAACAACCTGGATATCCTC
Minc_42_3     TCAACCACCAGCAGCTCCAACAACAAAACCTCCACGAAATTTATATCCATCTGGACCAGAACCTACTCCAAAACAACCTGGATATCCCCAAGGACCAGCATCTACTCCAAAACAACCTGGATATCCTC
Minc_42_4     TCAACCACCAGCAGCTCCAACAACAAAACCTCCACGAAATTTATATCCATCTGGACCAGAACCTACTCCAAAACAACCTGGATATCCCCAAGGACCAGCATCTACTCCAAAACAACCTGGATATCCTC
Minc_42_5     TCAACCACCAGCAGCTCCAACAACAAAACCTCCACGAAATTTATATCCATCTGGACCAGAACCTACTCCAAAACAACCTGGATATCCCCAAGGACCAGCATCTACTCCAAAACAACCTGGATATCCTC
Minc_42_7     TCAACCACCAGCAGCTCCAACAACAAAACCTCCACGAAATTTATATCCATCTGGACCAGAACCTACTCCAAAACAACCTGGATATCCCCAAGGACCAGCATCTACTCCAAAACAACCTGGATATCCTC
Minc_42_8     TCAACCACCAGCAGCTCCAACAACAAAACCTCCACGAAATTTATATCCATCTGGACCAGAACCTACTCCAAAACAACCTGGATATCCCCAAGGACCAGCATCTACTCCAAAACAACCTGGATATCCTC
Minc_42_9     TCAACCACCAGCAGCTCCAACAACAAAACCTCCACGAAATTTATATCCATCTGGACCAGAACCTACTCCAAAACAACCTGGATATCCCCAAGGACCAGCATCTACTCCAAAACAACCTGGATATCCTC
Minc_45_10    TCAACCACCAGCAGCTCCAACAACAAAACCTCCACGAAATTTATATCCATCTGGACCAGAACCTACTCCAAAACAACCTGGATATCCCCAAGGACCAGCATCTACTCCAAAACAACCTGGATATCCTC
Minc_45_11    TCAACCACCAGCAGCTCCAACAACAAAACCTCCACGAAATTTATATCCATCTGGACCAGAACCTACTCCAAAACAACCTGGATATCCCCAAGGACCAGCATCTACTCCAAAACAACCTGGATATCCTC
Minc_45_12    TCAACCACCAGCAGCTCCAACAACAAAACCTCCACGAAATTTATATCCATCTGGACCAGAACCTACTCCAAAACAACCTGGATATCCCCAAGGACCAGCATCTACTCCAAAACAACCTGGATATCCTC
Minc_45_13    TCAACCACCAGCAGCTCCAACAACAAAACCTCCACGAAATTTATATCCATCTGGACCAGAACCTACTCCAAAACAACCTGGATATCCCCAAGGACCAGCATCTACTCCAAAACAACCTGGATATCCTC
Minc_45_7     TCAACCACCAGCAGCTCCAACAACAAAACCTCCACGAAATTTATATCCATCTGGACCAGAACCTACTCCAAAACAACCTGGATATCCCCAAGGACCAGCATCTACTCCAAAACAACCTGGATATCCTC
Minc_45_9     TCAACCACCAGCAGCTCCAACAACAAAACCTCCACGAAATTTATATCCATCTGGACCAGAACCTACTCCAAAACAACCTGGATATCCCCAAGGACCAGCATCTACTCCAAAACAACCTGGATATCCTC
Minc_47_1     TCAACCACCAGCAGCTCCAACAACAAAACCTCCACGAAATTTATATCCATCTGGACCAGAACCTACTCCAAAACAACCTGGATATCCCCAAGGACCAGCATCTACTCCAAAACAACCTGGATATCCTC
Minc_47_10    TCAACCACCAGCAGCTCCAACAACAAAACCTCCACGAAATTTATATCCATCTGGACCAGAACCTACTCCAAAACAACCTGGATATCCCCAAGGACCAGCATCTACTCCAAAACAACCTGGATATCCTC
Minc_47_12    TCAACCACCAGCAGCTCCAACAACAAAACCTCCACGAAATTTATATCCATCTGGACCAGAACCTACTCCAAAACAACCTGGATATCCCCAAGGACCAGCATCTACTCCAAAACAACCTGGATATCCTC
Minc_47_13    TCAACCACCAGCAGCTCCAACAACAAAACCTCCACGAAATTTATATCCATCTGGACCAGAACCTACTCCAAAACAACCTGGATATCCCCAAGGACCAGCATCTACTCCAAAACAACCTGGATATCCTC
Minc_47_3     TCAACCACCAGCAGCTCCAACAACAAAACCTCCACGAAATTTATATCCATCTGGACCAGAACCTACTCCAAAACAACCTGGATATCCCCAAGGACCAGCATCTACTCCAAAACAACCTGGATATCCTC
Minc_47_7     TCAACCACCAGCAGCTCCAACAACAAAACCTCCACGAAATTTATATCCATCTGGACCAGAACCTACTCCAAAACAACCTGGATATCCCCAAGGACCAGCATCTACTCCAAAACAACCTGGATATCCTC
Minc_47_8     TCAACCACCAGCAGCTCCAACAACAAAACCTCCACGAAATTTATATCCATCTGGACCAGAACCTACTCCAAAACAACCTGGATATCCCCAAGGACCAGCATCTACTCCAAAACAACCTGGATATCCTC
Minc_51_10    TCAACCACCAGCAGCTCCAACAACAAAACCTCCACGAAATTTATATCCATCTGGACCAGAACCTACTCCAAAACAACCTGGATATCCCCAAGGACCAGCATCTACTCCAAAACAACCTGGATATCCTC
Minc_51_11    TCAACCACCAGCAGCTCCAACAACAAAACCTCCACGAAATTTATATCCATCTGGACCAGAACCTACTCCAAAACAACCTGGATATCCCCAAGGACCAGCATCTACTCCAAAACAACCTGGATATCCTC
Minc_51_4     TCAACCACCAGCAGCTCCAACAACAAAACCTCCACGAAATTTATATCCATCTGGACCAGAACCTACTCCAAAACAACCTGGATATCCCCAAGGACCAGCATCTACTCCAAAACAACCTGGATATCCTC
Minc_51_6     TCAACCACCAGCAGCTCCAACAACAAAACCTCCACGAAATTTATATCCATCTGGACCAGAACCTACTCCAAAACAACCTGGATATCCCCAAGGACCAGCATCTACTCCAAAACAACCTGGATATCCTC
Minc_51_7     TCAACCACCAGCAGCTCCAACAACAAAACCTCCACGAAATTTATATCCATCTGGACCAGAACCTACTCCAAAACAACCTGGATATCCCCAAGGACCAGCATCTACTCCAAAACAACCTGGATATCCTC
Minc_54_1     TCAACCACCAGCAGCTCCAACAACAAAACCTCCACGAAATTTATATCCATCTGGACCAGAACCTACTCCAAAACAACCTGGATATCCCCAAGGACCAGCATCTACTCCAAAACAACCTGGATATCCTC
Minc_54_10    TCAACCACCAGCAGCTCCAACAACAAAACCTCCACGAAATTTATATCCATCTGGACCAGAACCTACTCCAAAACAACCTGGATATCCCCAAGGACCAGCATCTACTCCAAAACAACCTGGATATCCTC
Minc_54_3     TCAACCACCAGCAGCTCCAACAACAAAACCTCCACGAAATTTATATCCATCTGGACCAGAACCTACTCCAAAACAACCTGGATATCCCCAAGGACCAGCATCTACTCCAAAACAACCTGGATATCCTC
Minc_54_4     TCAACCACCAGCAGCTCCAACAACAAAACCTCCACGAAATTTATATCCATCTGGACCAGAACCTACTCCAAAACAACCTGGATATCCCCAAGGACCAGCATCTACTCCAAAACAACCTGGATATCCTC
Minc_54_5     TCAACCACCAGCAGCTCCAACAACAAAACCTCCACGAAATTTATATCCATCTGGACCAGAACCTACTCCAAAACAACCTGGATATCCCCAAGGACCAGCATCTACTCCAAAACAACCTGGATATCCTC
Minc_54_6     TCAACCACCAGCAGCTCCAACAACAAAACCTCCACGAAATTTATATCCATCTGGACCAGAACCTACTCCAAAACAACCTGGATATCCCCAAGGACCAGCATCTACTCCAAAACAACCTGGATATCCTC
Minc_56_1     TCAACCACCAGCAGCTCCAACAACAAAACCTCCACGAAATTTATATCCATCTGGACCAGAACCTACTCCAAAACAACCTGGATATCCCCAAGGACCAGCATCTACTCCAAAACAACCTGGATATCCTC
Minc_56_12    TCAACCACCAGCAGCTCCAACAACAAAACCTCCACGAAATTTATATCCATCTGGACCAGAACCTACTCCAAAACAACCTGGATATCCCCAAGGACCAGCATCTACTCCAAAACAACCTGGATATCCTC
Minc_56_2     TCAACCACCAGCAGCTCCAACAACAAAACCTCCACGAAATTTATATCCATCTGGACCAGAACCTACTCCAAAACAACCTGGATATCCCCAAGGACCAGCATCTACTCCAAAACAACCTGGATATCCTC
Minc_56_3     TCAACCACCAGCAGCTCCAACAACAAAACCTCCACGAAATTTATATCCATCTGGACCAGAACCTACTCCAAAACAACCTGGATATCCCCAAGGACCAGCATCTACTCCAAAACAACCTGGATATCCTC
Minc_56_4     TCAACCACCAGCAGCTCCAACAACAAAACCTCCACGAAATTTATATCCATCTGGACCAGAACCTACTCCAAAACAACCTGGATATCCCCAAGGACCAGCATCTACTCCAAAACAACCTGGATATCCTC
Minc_56_5     TCAACCACCAGCAGCTCCAACAACAAAACCTCCACGAAATTTATATCCATCTGGACCAGAACCTACTCCAAAACAACCTGGATATCCCCAAGGACCAGCATCTACTCCAAAACAACCTGGATATCCTC
Minc_56_6     TCAACCACCAGCAGCTCCAACAACAAAACCTCCACGAAATTTATATCCATCTGGACCAGAACCTACTCCAAAACAACCTGGATATCCCCAAGGACCAGCATCTACTCCAAAACAACCTGGATATCCTC
Minc_56_7     TCAACCACCAGCAGCTCCAACAACAAAACCTCCACGAAATTTATATCCATCTGGACCAGAACCTACTCCAAAACAACCTGGATATCCCCAAGGACCAGCATCTACTCCAAAACAACCTGGATATCCTC
Minc_58_1     TCAACCACCAGCAGCTCCAACAACAAAACCTCCACGAAATTTATATCCATCTGGACCAGAACCTACTCCAAAACAACCTGGATATCCCCAAGGACCAGCATCTACTCCAAAACAACCTGGATATCCTC
Minc_58_13    TCAACCACCAGCAGCTCCAACAACAAAACCTCCACGAAATTTATATCCATCTGGACCAGAACCTACTCCAAAACAACCTGGATATCCCCAAGGACCAGCATCTACTCCAAAACAACCTGGATATCCTC
Minc_58_2     TCAACCACCAGCAGCTCCAACAACAAAACCTCCACGAAATTTATATCCATCTGGACCAGAACCTACTCCAAAACAACCTGGATATCCCCAAGGACCAGCATCTACTCCAAAACAACCTGGATATCCTC
Minc_58_3     TCAACCACCAGCAGCTCCAACAACAAAACCTCCACGAAATTTATATCCATCTGGACCAGAACCTACTCCAAAACAACCTGGATATCCCCAAGGACCAGCATCTACTCCAAAACAACCTGGATATCCTC
```

```
Minc_76_12   TACAACAGG-AGCTCA-CCAACTTCACGAAACGTTTACCCATCTGGGCCAGAATCATCTACACCAGGAGGACAAGTAGCATCGCATAAG-CCTCCGGCAGCACCTTCTACTCCACAGCCTGGATGTCA
Minc_76_13   TACAACAGG-AGCTCAACCAACTTCACGAAACGTTTACCCATCTGGGCCAGAATCATCTACACCAGGAGGACAAGTAGCATCGCATAAG-CCTCCGGCAGCACCTTCTACTCCACAGCCTGGATGTCA
Minc_76_14   TACAACAGG-AGCTCAACCAACTTCACGAAACGTTTACCCATCTGGGCCAGAATCATCTACACCAGGAGGACAAGTAGCATCGCATAAG-CCTCCGGCAGCACCTTCTACTCCACAGCCTGGATGTCA
Minc_76_3    TACAACAGG-AGCTCAACCAACTTCACGAAACGTTTACCCATCTGGGCCAGAATCATCTACACCAGGAGGACAAGTAGCATCGCATAAG-CCTCCGGCAGCACCTTCTACTCCACAGCCTGGATGTCA
Minc_76_7    TACAACAGG-AGCTCAACCAACTTCACGAAACGTTTACCCATCTGGGCCAGAATCATCTACACCAGGAGGACAAGTAGCATCGCATAA---CTCCGGCAGCACCTTCTACTCCACAGCCTGGATGTCA
Minc_76_8    TACAACAGG-AGCTCAACCAACTTCACGAAACGTTTACCCATCTGGGCCAGAATCATCTACACCAGGAGGACAAGTAGCATCGCATAAG-CCTCCGGCAGCACCTTCTACTCCACAGCCTGGATGTCA
Minc_76_9    TACAACAGG-AGCTCAACCAACTTCACGAAACGTTTACCCATCTGGGCCAGAATCATCTACACCAGGAGGACAAGTAGCATCGCATAAG-CCTCCGGCAGCACCTTCTACTCCACAGCCTGGATGTCA
Minc_78_1    TACAACAGG-AGCTCAACCAACTTCACGAAACGTTTACCCATCTGGGCCAGAATCATCTACACCAGGAGGACAAGTAGCATCGCATAA---CTCCGGCAGCACCTTCTACTCCACAGCCTGGATGTCA
Minc_78_10   TACAACAGG-AGCTCAACCAACTTCACGAAACGTTTACCCATCTGGGCCAGAATCATCTACACCAGGAGGACAAGTAGCATCGCATAAG-CCTCCGGCAGCACCTTCTACTCCACAGCCTGGATGTCA
Minc_78_11   TACAACAGG-AGCTCAACCAACTTCACGAAACGTTTACCCATCTGGGCCAGAATCATCTACACCAGGAGGACAAGTAGCATCGCATAAG-CCTCCGGCAGCACCTTCTACTCCACAGCCTGGATGTCA
Minc_78_13   TACAACAGG-AGCTCAACCAACTTCACGAAACGTTTACCCATCTGGGCCAGAATCATCTACACCAGGAGGACAAGTAGCATCGCATAAG-CCTCCGGCAGCACCTTCTACTCCACAGCCTGGATGTCA
Minc_78_15   TACAACAGG-AGCTCAACCAACTTCACGAAACGTTTACCCATCTGGGCCAGAATCATCTACACCAGGAGGACAAGTAGCATCGCATAA--C-TCCGGCAGCACCTTCTACTCCACAGCCTGGATGTCA
Minc_78_16   TACAACAGG-AGCTCAACCAACTTCACGAAACGTTTACCCATCTGGGCCAGAATCATCTACACCAGGAGGACAAGTAGCATCGCATAA--C-TCCGGCAGCACCTTCTACTCCACAGCCTGGATGTCA
Minc_78_17   TACAACAGG-AGCTCAACCAACTTCACGAAACGTTTACCCATCTGGGCCAGAATCATCTACACCAGGAGGACAAGTAGCATCGCATAA---CTCCGGCAGCACCTTCTACTCCACAGCCTGGATGTCA
Minc_78_7    TACAACAGG-AGCTCAACCAACTTCACGAAACGTTTACCCATCTGGGCCAGAATCATCTACACCAGGAGGACAAGTAGCATCGCATAAG-CCTCCGGCAGCACCTTCTACTCCACAGCCTGGATGTCA
Minc_81_12   TACAACAGG-AGCTCAACCAACTTCACGAAACGTTTACCCATCTGGGCCAGAATCATCTACACCAGGAGGACAAGTAGCATCGCATAAG-CCTCCGGCAGCACCTTCTACTCCACAGCCTGGATGTCA
Minc_81_13   TACAACAGG-AGCTCAACCAACTTCACGAAACGTTTACCCATCTGGGCCAGAATCATCTACACCAGGAGGACAAGTAGCATCGCATAA--C-TCCGGCAGCACCTTCTACTCCACAGCCTGGATGTCA
Minc_81_14   TACAACAGG-AGCTCAACCAACTTCACGAAACGTTTACCCATCTGGGCCAGAATCATCTACACCAGGAGGACAAGTAGCATCGCATAAG-CCTCCGGCAGCACCTTCTACTCCACAGCCTGGATGTCA
Minc_81_15   TACAACAGG-AGCTCAACCAACTTCACGAAACGTTTACCCATCTGGGCCAGAATCATCTACACCAGGAGGACAAGTAGCATC-CATAAG-CCTCCGG-AGCACCTTCTACTCCACAGCCTGGATGTCA
Minc_81_2    TACAACAGG-AGCTCAACCAACTTCACGAAACGTTTACCCATCTGGGCCAGAATCATCTACACCAGGAGGACAAGTAGCATCGCATAAG-CCTCCGGCAGCACCTTCTACTCCACAGCCTGGATGTCA
Minc_81_4    TACAACAGG-AGCTCAACCAACTTCACGAAACGTTTACCCATCTGGGCCAGAATCATCTACACCAGGAGGACAAGTAGCATCGCATAAG-CCTCCGGCAGCACCTTCTACTCCACAGCCTGGATGTCA
Minc_81_6    TACAACAGG-AGCTCAACCAACTTCACGAAACGTTTACCCATCTGGGCCAGAATCATCTACACCAGGAGGACAAGTAGCATCGCATAAG-CCTCCGGCAGCACCTTCTACTCCACAGCCTGGATGTCA
Minc_81_8    TACAACAGG-AGCTCAACCAACTTCACGAAACGTTTACCCATCTGGGCCAGAATCATCTACACCAGGAGGACAAGTAGCATCGCATAAG-CCTCCGGCAGCACCTTCTACTCCACAGCCTGGATGTCA
             ********* ****** ***************************************************************** ****      **** ******************************
```

```
             TCAACCACCAGCAGCTCCAACAACAAAACCTCCACGAAATTTATATCATCTGGACCAGAACCTACTCCAAAACAACCTGGATATCCCCAAGGACCAGCATCTACTCCAAAACAACCTGGATATCCTC
Minc_18_1    TCAACCACCAGCAGCTCCAACAACAAAACCTCCACGAAATTTATATCATCTGGACCAGAACCTACTCCAAAACAACCTGGATATCCCCAAGGACCAGCATCTACTCCAAAACAACCTGGATATCCTC
Minc_18_10   TCAACCACCAGCAGCTCCAACAACAAAACCTCCACGAAATTTATATCATCTGGACCAGAACCTACTCCAAAACAACCTGGATATCCCCAAGGACCAGCATCTACTCCAAAACAACCTGGATATCCTC
Minc_18_11   TCAACCACCAGCAGCTCCAACAACAAAACCTCCACGAAATTTATATCATCTGGACCAGAACCTACTCCAAAACAACCTGGATATCCCCAAGGACCAGCATCTACTCCAAAACAACCTGGATATCCTC
Minc_18_13   TCAACCACCAGCAGCTCCAACAACAAAACCTCCACGAAATTTATATCATCTGGACCAGAACCTACTCCAAAACAACCTGGATATCCCCAAGGACCAGCATCTACTCCAAAACAACCTGGATATCCTC
Minc_18_3    TCAACCACCAGCAGCTCCAACAACAAAACCTCCACGAAATTTATATCATCTGGACCAGAACCTACTCCAAAACAACCTGGATATCCCCAAGGACCAGCATCTACTCCAAAACAACCTGGATATCCTC
Minc_18_4    TCAACCACCAGCAGCTCCAACAACAAAACCTCCACGAAATTTATATCATCTGGACCAGAACCTACTCCAAAACAACCTGGATATCCCCAAGGACCAGCATCTACTCCAAAACAACCTGGATATCCTC
Minc_18_5    TCAACCACCAGCAGCTCCAACAACAAAACCTCCACGAAATTTATATCATCTGGACCAGAACCTACTCCAAAACAACCTGGATATCCCCAAGGACCAGCATCTACTCCAAAACAACCTGGATATCCTC
Minc_18_7    TCAACCACCAGCAGCTCCAACAACAAAACCTCCACGAAATTTATATCATCTGGACCAGAACCTACTCCAAAACAACCTGGATATCCCCAAGGACCAGCATCTACTCCAAAACAACCTGGATATCCTC
Minc_18_8    TCAACCACCAGCAGCTCCAACAACAAAACCTCCACGAAATTTATATCATCTGGACCAGAACCTACTCCAAAACAACCTGGATATCCCCAAGGACCAGCATCTACTCCAAAACAACCTGGATATCCTC
Minc_18_9    TCAACCACCAGCAGCTCCAACAACAAAACCTCCACGAAATTTATATCATCTGGACCAGAACCTACTCCAAAACAACCTGGATATCCCCAAGGACCAGCATCTACTCCAAAACAACCTGGATATCCTC
Minc_22_14   TCAACCACCAGCAGCTCCAACAACAAAACCTCCACGAAATTTATATCATCTGGACCAGAACCTACTCCAAAACAACCTGGATATCCCCAAGGACCAGCATCTACTCCAAAACAACCTGGATATCCTC
Minc_22_15   TCAACCACCAGCAGCTCCAACAACAAAACCTCCACGAAATTTATATCATCTGGACCAGAACCTACTCCAAAACAACCTGGATATCCCCAAGGACCAGCATCTACTCCAAAACAACCTGGATATCCTC
Minc_22_2    TCAACCACCAGCAGCTCCAACAACAAAACCTCCACGAAATTTATATCATCTGGACCAGAACCTACTCCAAAACAACCTGGATATCCCCAAGGACCAGCATCTACTCCAAAACAACCTGGATATCCTC
Minc_22_3    TCAACCACCAGCAGCTCCAACAACAAAACCTCCACGAAATTTATATCATCTGGACCAGAACCTACTCCAAAACAACCTGGATATCCCCAAGGACCAGCATCTACTCCAAAACAACCTGGATATCCTC
Minc_22_5    TCAACCACCAGCAGCTCCAACAACAAAACCTCCACGAAATTTATATCATCTGGACCAGAACCTACTCCAAAACAACCTGGATATCCCCAAGGACCAGCATCTACTCCAAAACAACCTGGATATCCTC
Minc_22_7    TCAACCACCAGCAGCTCCAACAACAAAACCTCCACGAAATTTATATCATCTGGACCAGAACCTACTCCAAAACAACCTGGATATCCCCAAGGACCAGCATCTACTCCAAAACAACCTGGATATCCTC
Minc_22_8    TCAACCACCAGCAGCTCCAACAACAAAACCTCCACGAAATTTATATCATCTGGACCAGAACCTACTCCAAAACAACCTGGATATCCCCAAGGACCAGCATCTACTCCAAAACAACCTGGATATCCTC
Minc_30_1    TCAACCACCAGCAGCTCCAACAACAAAACCTCCACGAAATTTATATCATCTGGACCAGAACCTACTCCAAAACAACCTGGATATCCCCAAGGACCAGCATCTACTCCAAAACAACCTGGATATCCTC
Minc_30_10   TCAACCACCAGCAGCTCCAACAACAAAACCTCCACGAAATTTATATCATCTGGACCAGAACCTACTCCAAAACAACCTGGATATCCCCAAGGACCAGCATCTACTCCAAAACAACCTGGATATCCTC
Minc_30_11   TCAACCACCAGCAGCTCCAACAACAAAACCTCCACGAAATTTATATCATCTGGACCAGAACCTACTCCAAAACAACCTGGATATCCCCAAGGACCAGCATCTACTCCAAAACAACCTGGATATCCTC
Minc_30_4    TCAACCACCAGCAGCTCCAACAACAAAACCTCCACGAAATTTATATCATCTGGACCAGAACCTACTCCAAAACAACCTGGATATCCCCAAGGACCAGCATCTACTCCAAAACAACCTGGATATCCTC
Minc_30_5    TCAACCACCAGCAGCTCCAACAACAAAACCTCCACGAAATTTATATCATCTGGACCAGAACCTACTCCAAAACAACCTGGATATCCCCAAGGACCAGCATCTACTCCAAAACAACCTGGATATCCTC
Minc_34_1    TCAACCACCAGCAGCTCCAACAACAAAACCTCCACGAAATTTATATCATCTGGACCAGAACCTACTCCAAAACAACCTGGATATCCCCAAGGACCAGCATCTACTCCAAAACAACCTGGATATCCTC
Minc_34_10   TCAACCACCAGCAGCTCCAACAACAAAACCTCCACGAAATTTATATCATCTGGACCAGAACCTACTCCAAAACAACCTGGATATCCCCAAGGACCAGCATCTACTCCAAAACAACCTGGATATCCTC
Minc_34_11   TCAACCACCAGCAGCTCCAACAACAAAACCTCCACGAAATTTATATCATCTGGACCAGAACCTACTCCAAAACAACCTGGATATCCCCAAGGACCAGCATCTACTCCAAAACAACCTGGATATCCTC
Minc_34_12   TCAACCACCAGCAGCTCCAACAACAAAACCTCCACGAAATTTATATCATCTGGACCAGAACCTACTCCAAAACAACCTGGATATCCCCAAGGACCAGCATCTACTCCAAAACAACCTGGATATCCTC
Minc_34_3    TCAACCACCAGCAGCTCCAACAACAAAACCTCCACGAAATTTATATCATCTGGACCAGAACCTACTCCAAAACAACCTGGATATCCCCAAGGACCAGCATCTACTCCAAAACAACCTGGATATCCTC
Minc_34_5    TCAACCACCAGCAGCTCCAACAACAAAACCTCCACGAAATTTATATCATCTGGACCAGAACCTACTCCAAAACAACCTGGATATCCCCAAGGACCAGCATCTACTCCAAAACAACCTGGATATCCTC
Minc_34_7    TCAACCACCAGCAGCTCCAACAACAAAACCTCCACGAAATTTATATCATCTGGACCAGAACCTACTCCAAAACAACCTGGATATCCCCAAGGACCAGCATCTACTCCAAAACAACCTGGATATCCTC
Minc_34_9    TCAACCACCAGCAGCTCCAACAACAAAACCTCCACGAAATTTATATCATCTGGACCAGAACCTACTCCAAAACAACCTGGATATCCCCAAGGACCAGCATCTACTCCAAAACAACCTGGATATCCTC
Minc_37_10   TCAACCACCAGCAGCTCCAACAACAAAACCTCCACGAAATTTATATCATCTGGACCAGAACCTACTCCAAAACAACCTGGATATCCCCAAGGACCAGCATCTACTCCAAAACAACCTGGATATCCTC
Minc_37_12   TCAACCACCAGCAGCTCCAACAACAAAACCTCCACGAAATTTATATCATCTGGACCAGAACCTACTCCAAAACAACCTGGATATCCCCAAGGACCAGCATCTACTCCAAAACAACCTGGATATCCTC
Minc_37_2    TCAACCACCAGCAGCTCCAACAACAAAACCTCCACGAAATTTATATCATCTGGACCAGAACCTACTCCAAAACAACCTGGATATCCCCAAGGACCAGCATCTACTCCAAAACAACCTGGATATCCTC
Minc_37_3    TCAACCACCAGCAGCTCCAACAACAAAACCTCCACGAAATTTATATCATCTGGACCAGAACCTACTCCAAAACAACCTGGATATCCCCAAGGACCAGCATCTACTCCAAAACAACCTGGATATCCTC
Minc_37_4    TCAACCACCAGCAGCTCCAACAACAAAACCTCCACGAAATTTATATCATCTGGACCAGAACCTACTCCAAAACAACCTGGATATCCCCAAGGACCAGCATCTACTCCAAAACAACCTGGATATCCTC
Minc_37_5    TCAACCACCAGCAGCTCCAACAACAAAACCTCCACGAAATTTATATCATCTGGACCAGAACCTACTCCAAAACAACCTGGATATCCCCAAGGACCAGCATCTACTCCAAAACAACCTGGATATCCTC
Minc_37_6    TCAACCACCAGCAGCTCCAACAACAAAACCTCCACGAAATTTATATCATCTGGACCAGAACCTACTCCAAAACAACCTGGATATCCCCAAGGACCAGCATCTACTCCAAAACAACCTGGATATCCTC
Minc_37_8    TCAACCACCAGCAGCTCCAACAACAAAACCTCCACGAAATTTATATCATCTGGACCAGAACCTACTCCAAAACAACCTGGATATCCCCAAGGACCAGCATCTACTCCAAAACAACCTGGATATCCTC
Minc_37_9    TCAACCACCAGCAGCTCCAACAACAAAACCTCCACGAAATTTATATCATCTGGACCAGAACCTACTCCAAAACAACCTGGATATCCCCAAGGACCAGCATCTACTCCAAAACAACCTGGATATCCTC
Minc_42_1    TCAACCACCAGCAGCTCCAACAACAAAACCTCCACGAAATTTATATCATCTGGACCAGAACCTACTCCAAAACAACCTGGATATCCCCAAGGACCAGCATCTACTCCAAAACAACCTGGATATCCTC
Minc_42_10   TCAACCACCAGCAGCTCCAACAACAAAACCTCCACGAAATTTATATCATCTGGACCAGAACCTACTCCAAAACAACCTGGATATCCCCAAGGACCAGCATCTACTCCAAAACAACCTGGATATCCTC
Minc_42_11   TCAACCACCAGCAGCTCCAACAACAAAACCTCCACGAAATTTATATCATCTGGACCAGAACCTACTCCAAAACAACCTGGATATCCCCAAGGACCAGCATCTACTCCAAAACAACCTGGATATCCTC
Minc_42_12   TCAACCACCAGCAGCTCCAACAACAAAACCTCCACGAAATTTATATCATCTGGACCAGAACCTACTCCAAAACAACCTGGATATCCCCAAGGACCAGCATCTACTCCAAAACAACCTGGATATCCTC
Minc_42_3    TCAACCACCAGCAGCTCCAACAACAAAACCTCCACGAAATTTATATCATCTGGACCAGAACCTACTCCAAAACAACCTGGATATCCCCAAGGACCAGCATCTACTCCAAAACAACCTGGATATCCTC
Minc_42_4    TCAACCACCAGCAGCTCCAACAACAAAACCTCCACGAAATTTATATCATCTGGACCAGAACCTACTCCAAAACAACCTGGATATCCCCAAGGACCAGCATCTACTCCAAAACAACCTGGATATCCTC
Minc_42_5    TCAACCACCAGCAGCTCCAACAACAAAACCTCCACGAAATTTATATCATCTGGACCAGAACCTACTCCAAAACAACCTGGATATCCCCAAGGACCAGCATCTACTCCAAAACAACCTGGATATCCTC
Minc_42_7    TCAACCACCAGCAGCTCCAACAACAAAACCTCCACGAAATTTATATCATCTGGACCAGAACCTACTCCAAAACAACCTGGATATCCCCAAGGACCAGCATCTACTCCAAAACAACCTGGATATCCTC
Minc_42_8    TCAACCACCAGCAGCTCCAACAACAAAACCTCCACGAAATTTATATCATCTGGACCAGAACCTACTCCAAAACAACCTGGATATCCCCAAGGACCAGCATCTACTCCAAAACAACCTGGATATCCTC
Minc_42_9    TCAACCACCAGCAGCTCCAACAACAAAACCTCCACGAAATTTATATCATCTGGACCAGAACCTACTCCAAAACAACCTGGATATCCCCAAGGACCAGCATCTACTCCAAAACAACCTGGATATCCTC
Minc_45_10   TCAACCACCAGCAGCTCCAACAACAAAACCTCCACGAAATTTATATCATCTGGACCAGAACCTACTCCAAAACAACCTGGATATCCCCAAGGACCAGCATCTACTCCAAAACAACCTGGATATCCTC
Minc_45_11   TCAACCACCAGCAGCTCCAACAACAAAACCTCCACGAAATTTATATCATCTGGACCAGAACCTACTCCAAAACAACCTGGATATCCCCAAGGACCAGCATCTACTCCAAAACAACCTGGATATCCTC
Minc_45_12   TCAACCACCAGCAGCTCCAACAACAAAACCTCCACGAAATTTATATCATCTGGACCAGAACCTACTCCAAAACAACCTGGATATCCCCAAGGACCAGCATCTACTCCAAAACAACCTGGATATCCTC
Minc_45_13   TCAACCACCAGCAGCTCCAACAACAAAACCTCCACGAAATTTATATCATCTGGACCAGAACCTACTCCAAAACAACCTGGATATCCCCAAGGACCAGCATCTACTCCAAAACAACCTGGATATCCTC
Minc_45_7    TCAACCACCAGCAGCTCCAACAACAAAACCTCCACGAAATTTATATCATCTGGACCAGAACCTACTCCAAAACAACCTGGATATCCCCAAGGACCAGCATCTACTCCAAAACAACCTGGATATCCTC
Minc_45_9    TCAACCACCAGCAGCTCCAACAACAAAACCTCCACGAAATTTATATCATCTGGACCAGAACCTACTCCAAAACAACCTGGATATCCCCAAGGACCAGCATCTACTCCAAAACAACCTGGATATCCTC
Minc_47_1    TCAACCACCAGCAGCTCCAACAACAAAACCTCCACGAAATTTATATCATCTGGACCAGAACCTACTCCAAAACAACCTGGATATCCCCAAGGACCAGCATCTACTCCAAAACAACCTGGATATCCTC
Minc_47_10   TCAACCACCAGCAGCTCCAACAACAAAACCTCCACGAAATTTATATCATCTGGACCAGAACCTACTCCAAAACAACCTGGATATCCCCAAGGACCAGCATCTACTCCAAAACAACCTGGATATCCTC
Minc_47_12   TCAACCACCAGCAGCTCCAACAACAAAACCTCCACGAAATTTATATCATCTGGACCAGAACCTACTCCAAAACAACCTGGATATCCCCAAGGACCAGCATCTACTCCAAAACAACCTGGATATCCTC
Minc_47_13   TCAACCACCAGCAGCTCCAACAACAAAACCTCCACGAAATTTATATCATCTGGACCAGAACCTACTCCAAAACAACCTGGATATCCCCAAGGACCAGCATCTACTCCAAAACAACCTGGATATCCTC
Minc_47_3    TCAACCACCAGCAGCTCCAACAACAAAACCTCCACGAAATTTATATCATCTGGACCAGAACCTACTCCAAAACAACCTGGATATCCCCAAGGACCAGCATCTACTCCAAAACAACCTGGATATCCTC
Minc_47_7    TCAACCACCAGCAGCTCCAACAACAAAACCTCCACGAAATTTATATCATCTGGACCAGAACCTACTCCAAAACAACCTGGATATCCCCAAGGACCAGCATCTACTCCAAAACAACCTGGATATCCTC
Minc_47_8    TCAACCACCAGCAGCTCCAACAACAAAACCTCCACGAAATTTATATCATCTGGACCAGAACCTACTCCAAAACAACCTGGATATCCCCAAGGACCAGCATCTACTCCAAAACAACCTGGATATCCTC
Minc_51_10   TCAACCACCAGCAGCTCCAACAACAAAACCTCCACGAAATTTATATCATCTGGACCAGAACCTACTCCAAAACAACCTGGATATCCCCAAGGACCAGCATCTACTCCAAAACAACCTGGATATCCTC
Minc_51_11   TCAACCACCAGCAGCTCCAACAACAAAACCTCCACGAAATTTATATCATCTGGACCAGAACCTACTCCAAAACAACCTGGATATCCCCAAGGACCAGCATCTACTCCAAAACAACCTGGATATCCTC
Minc_51_4    TCAACCACCAGCAGCTCCAACAACAAAACCTCCACGAAATTTATATCATCTGGACCAGAACCTACTCCAAAACAACCTGGATATCCCCAAGGACCAGCATCTACTCCAAAACAACCTGGATATCCTC
Minc_51_6    TCAACCACCAGCAGCTCCAACAACAAAACCTCCACGAAATTTATATCATCTGGACCAGAACCTACTCCAAAACAACCTGGATATCCCCAAGGACCAGCATCTACTCCAAAACAACCTGGATATCCTC
Minc_51_7    TCAACCACCAGCAGCTCCAACAACAAAACCTCCACGAAATTTATATCATCTGGACCAGAACCTACTCCAAAACAACCTGGATATCCCCAAGGACCAGCATCTACTCCAAAACAACCTGGATATCCTC
Minc_54_1    TCAACCACCAGCAGCTCCAACAACAAAACCTCCACGAAATTTATATCATCTGGACCAGAACCTACTCCAAAACAACCTGGATATCCCCAAGGACCAGCATCTACTCCAAAACAACCTGGATATCCTC
Minc_54_10   TCAACCACCAGCAGCTCCAACAACAAAACCTCCACGAAATTTATATCATCTGGACCAGAACCTACTCCAAAACAACCTGGATATCCCCAAGGACCAGCATCTACTCCAAAACAACCTGGATATCCTC
Minc_54_3    TCAACCACCAGCAGCTCCAACAACAAAACCTCCACGAAATTTATATCATCTGGACCAGAACCTACTCCAAAACAACCTGGATATCCCCAAGGACCAGCATCTACTCCAAAACAACCTGGATATCCTC
Minc_54_4    TCAACCACCAGCAGCTCCAACAACAAAACCTCCACGAAATTTATATCATCTGGACCAGAACCTACTCCAAAACAACCTGGATATCCCCAAGGACCAGCATCTACTCCAAAACAACCTGGATATCCTC
Minc_54_5    TCAACCACCAGCAGCTCCAACAACAAAACCTCCACGAAATTTATATCATCTGGACCAGAACCTACTCCAAAACAACCTGGATATCCCCAAGGACCAGCATCTACTCCAAAACAACCTGGATATCCTC
Minc_54_6    TCAACCACCAGCAGCTCCAACAACAAAACCTCCACGAAATTTATATCATCTGGACCAGAACCTACTCCAAAACAACCTGGATATCCCCAAGGACCAGCATCTACTCCAAAACAACCTGGATATCCTC
Minc_56_1    TCAACCACCAGCAGCTCCAACAACAAAACCTCCACGAAATTTATATCATCTGGACCAGAACCTACTCCAAAACAACCTGGATATCCCCAAGGACCAGCATCTACTCCAAAACAACCTGGATATCCTC
Minc_56_12   TCAACCACCAGCAGCTCCAACAACAAAACCTCCACGAAATTTATATCATCTGGACCAGAACCTACTCCAAAACAACCTGGATATCCCCAAGGACCAGCATCTACTCCAAAACAACCTGGATATCCTC
Minc_56_2    TCAACCACCAGCAGCTCCAACAACAAAACCTCCACGAAATTTATATCATCTGGACCAGAACCTACTCCAAAACAACCTGGATATCCCCAAGGACCAGCATCTACTCCAAAACAACCTGGATATCCTC
Minc_56_3    TCAACCACCAGCAGCTCCAACAACAAAACCTCCACGAAATTTATATCATCTGGACCAGAACCTACTCCAAAACAACCTGGATATCCCCAAGGACCAGCATCTACTCCAAAACAACCTGGATATCCTC
Minc_56_4    TCAACCACCAGCAGCTCCAACAACAAAACCTCCACGAAATTTATATCATCTGGACCAGAACCTACTCCAAAACAACCTGGATATCCCCAAGGACCAGCATCTACTCCAAAACAACCTGGATATCCTC
Minc_56_5    TCAACCACCAGCAGCTCCAACAACAAAACCTCCACGAAATTTATATCATCTGGACCAGAACCTACTCCAAAACAACCTGGATATCCCCAAGGACCAGCATCTACTCCAAAACAACCTGGATATCCTC
Minc_56_6    TCAACCACCAGCAGCTCCAACAACAAAACCTCCACGAAATTTATATCATCTGGACCAGAACCTACTCCAAAACAACCTGGATATCCCCAAGGACCAGCATCTACTCCAAAACAACCTGGATATCCTC
Minc_56_7    TCAACCACCAGCAGCTCCAACAACAAAACCTCCACGAAATTTATATCATCTGGACCAGAACCTACTCCAAAACAACCTGGATATCCCCAAGGACCAGCATCTACTCCAAAACAACCTGGATATCCTC
Minc_58_1    TCAACCACCAGCAGCTCCAACAACAAAACCTCCACGAAATTTATATCATCTGGACCAGAACCTACTCCAAAACAACCTGGATATCCCCAAGGACCAGCATCTACTCCAAAACAACCTGGATATCCTC
Minc_58_13   TCAACCACCAGCAGCTCCAACAACAAAACCTCCACGAAATTTATATCATCTGGACCAGAACCTACTCCAAAACAACCTGGATATCCCCAAGGACCAGCATCTACTCCAAAACAACCTGGATATCCTC
Minc_58_2    TCAACCACCAGCAGCTCCAACAACAAAACCTCCACGAAATTTATATCATCTGGACCAGAACCTACTCCAAAACAACCTGGATATCCCCAAGGACCAGCATCTACTCCAAAACAACCTGGATATCCTC
Minc_58_3    TCAACCACCAGCAGCTCCAACAACAAAACCTCCACGAAATTTATATCATCTGGACCAGAACCTACTCCAAAACAACCTGGATATCCCCAAGGACCAGCATCTACTCCAAAACAACCTGGATATCCTC
```

```
Minc_54_4    AAGGACCAGCATCTACTCCAAAACAACCTGGATATCCTCAAGGACGAGCATCACCAACTCCACGAAACGTTTACCCATCTGGACCAGAACCATCTACACCAGGAGGAAAAGTAGCATCGCATAAGCCT
Minc_54_5    AAGGACCAGCATCTACTCCAAAACAACCTGGATATCCTCAAGGACGAGCATCACCAACTCCACGAAACGTTTACCCATCTGGACCAGAACCATCTACACCAGGAGGAAAAGTAGCATCGCATAAGCCT
Minc_54_6    AAGGACCAGCATCTACTCCAAAACAACCTGGATATCCTCAAGGACGAGCATCACCAACTCCACGAAACGTTTACCCATCTGGACCAGAACCATCTACACCAGGAGGAAAAGTAGCATCGCATAAGCCT
Minc_56_1    AAGGACCAGCATCTACTCCAAAACAACCTGGATATCCTCAAGGACGAGCATCACCAACTCCACGAAACGTTTACCCATCTGGACCAGAACCATCTACACCAGGAGGAAAAGTAGCATCGCATAAGCCT
Minc_56_12   AAGGACCAGCATCTACTCCAAAACAACCTGGATATCCTCAAGGACGAGCATCACCAACTCCACGAAACGTTTACCCATCTGGACCAGAACCATCTACACCAGGAGGAAAAGTAGCATCGCATAAGCCT
Minc_56_2    AAGGACCAGCATCTACTCCAAAACAACCTGGATATCCTCAAGGACGAGCATCACCAACTCCACGAAACGTTTACCCATCTGGACCAGAACCATCTACACCAGGAGGAAAAGTAGCATCGCATAAGCCT
Minc_56_3    AAGGACCAGCATCTACTCCAAAACAACCTGGATATCCTCAAGGACGAGCATCACCAACTCCACGAAACGTTTACCCATCTGGACCAGAACCATCTACACCAGGAGGAAAAGTAGCATCGCATAAGCCT
Minc_56_4    AAGGACCAGCATCTACTCCAAAACAACCTGGATATCCTCAAGGACGAGCATCACCAACTCCACGAAACGTTTACCCATCTGGACCAGAACCATCTACACCAGGAGGAAAAGTAGCATCGCATAAGCCT
Minc_56_5    AAGGACCAGCATCTACTCCAAAACAACCTGGATATCCTCAAGGACGAGCATCACCAACTCCACGAAACGTTTACCCATCTGGACCAGAACCATCTACACCAGGAGGAAAAGTAGCATCGCATAAGCCT
Minc_56_6    AAGGACCAGCATCTACTCCAAAACAACCTGGATATCCTCAAGGACGAGCATCACCAACTCCACGAAACGTTTACCCATCTGGACCAGAACCATCTACACCAGGAGGAAAAGTAGCATCGCATAAGCCT
Minc_56_7    AAGGACCAGCATCTACTCCAAAACAACCTGGATATCCTCAAGGACGAGCATCACCAACTCCACGAAACGTTTACCCATCTGGACCAGAACCATCTACACCAGGAGGAAAAGTAGCATCGCATAAGCCT
Minc_58_1    AAGGACCAGCATCTACTCCAAAACAACCTGGATATCCTCAAGGACGAGCATCACCAACTCCACGAAACGTTTACCCATCTGGACCAGAACCATCTACACCAGGAGGAAAAGTAGCATCGCATAAGCCT
Minc_58_13   AAGGACCAGCATCTACTCCAAAACAACCTGGATATCCTCAAGGACGAGCATCACCAACTCCACGAAACGTTTACCCATCTGGACCAGAACCATCTACACCAGGAGGAAAAGTAGCATCGCATAAGCCT
Minc_58_2    AAGGACCAGCATCTACTCCAAAACAACCTGGATATCCTCAAGGACGAGCATCACCAACTCCACGAAACGTTTACCCATCTGGACCAGAACCATCTACACCAGGAGGAAAAGTAGCATCGCATAAGCCT
Minc_58_3    AAGGACCAGCATCTACTCCAAAACAACCTGGATATCCTCAAGGACGAGCATCACCAACTCCACGAAACGTTTACCCATCTGGACCAGAACCATCTACACCAGGAGGAAAAGTAGCATCGCATAAGCCT
Minc_58_4    AAGGACCAGCATCTACTCCAAAACAACCTGGATATCCTCAAGGACGAGCATCACCAACTCCACGAAACGTTTACCCATCTGGACCAGAACCATCTACACCAGGAGGAAAAGTAGCATCGCATAAGCCT
Minc_58_5    AAGGACCAGCATCTACTCCAAAACAACCTGGATATCCTCAAGGACGAGCATCACCAACTCCACGAAACGTTTACCCATCTGGACCAGAACCATCTACACCAGGAGGAAAAGTAGCATCGCATAAGCCT
Minc_58_6    AAGGACCAGCATCTACTCCAAAACAACCTGGATATCCTCAAGGACGAGCATCACCAACTCCACGAAACGTTTACCCATCTGGACCAGAACCATCTACACCAGGAGGAAAAGTAGCATCGCATAAGCCT
Minc_58_7    AAGGACCAGCATCTACTCCAAAACAACCTGGATATCCTCAAGGACGAGCATCACCAACTCCACGAAACGTTTACCCATCTGGACCAGAACCATCTACACCAGGAGGAAAAGTAGCATCGCATAAGCCT
Minc_58_8    AAGGACCAGCATCTACTCCAAAACAACCTGGATATCCTCAAGGACGAGCATCACCAACTCCACGAAACGTTTACCCATCTGGACCAGAACCATCTACACCAGGAGGAAAAGTAGCATCGCATAAGCCT
Minc_58_9    AAGGACCAGCATCTACTCCAAAACAACCTGGATATCCTCAAGGACGAGCATCACCAACTCCACGAAACGTTTACCCATCTGGACCAGAACCATCTACACCAGGAGGAAAAGTAGCATCGCATAAGCCT
Minc_65_1    AAGGACCAGCATCTACTCCAAAACAACCTGGATATCCTCAAGGACGAGCATCACCAACTCCACGAAACGTTTACCCATCTGGACCAGAACCATCTACACCAGGAGGAAAAGTAGCATCGCATAAGCCT
Minc_65_11   AAGGACCAGCATCTACTCCAAAACAACCTGGATATCCTCAAGGACGAGCATCACCAACTCCACGAAACGTTTACCCATCTGGACCAGAACCATCTACACCAGGAGGAAAAGTAGCATCGCATAAGCCT
Minc_65_3    AAGGACCAGCATCTACTCCAAAACAACCTGGATATCCTCAAGGACGAGCATCACCAACTCCACGAAACGTTTACCCATCTGGACCAGAACCATCTACACCAGGAGGAAAAGTAGCATCGCATAAGCCT
Minc_65_6    AAGGACCAGCATCTACTCCAAAACAACCTGGATATCCTCAAGGACGAGCATCACCAACTCCACGAAACGTTTACCCATCTGGACCAGAACCATCTACACCAGGAGGAAAAGTAGCATCGCATAAGCCT
Minc_65_8    AAGGACCAGCATCTACTCCAAAACAACCTGGATATCCTCAAGGACGAGCATCACCAACTCCACGAAACGTTTACCCATCTGGACCAGAACCATCTACACCAGGAGGAAAAGTAGCATCGCATAAGCCT
Minc_65_9    AAGGACCAGCATCTACTCCAAAACAACCTGGATATCCTCAAGGACGAGCATCACCAACTCCACGAAACGTTTACCCATCTGGACCAGAACCATCTACACCAGGAGGAAAAGTAGCATCGCATAAGCCT
Minc_76_1    AAGGACCAGCATCTACTCCAAAACAACCTGGATATCCTCAAGGACGAGCATCACCAACTCCACGAAACGTTTACCCATCTGGACCAGAACCATCTACACCAGGAGGAAAAGTAGCATCGCATAAGCCT
Minc_76_10   AAGGACCAGCATCTACTCCAAAACAACCTGGATATCCTCAAGGACGAGCATCACCAACTCCACGAAACGTTTACCCATCTGGACCAGAACCATCTACACCAGGAGGAAAAGTAGCATCGCATAAGCCT
Minc_76_12   AAGGACCAGCATCTACTCCAAAACAACCTGGATATCCTCAAGGACGAGCATCACCAACTCCACGAAACGTTTACCCATCTGGACCAGAACCATCTACACCAGGAGGAAAAGTAGCATCGCATAAGCCT
Minc_76_13   AAGGACCAGCATCTACTCCAAAACAACCTGGATATCCTCAAGGACGAGCATCACCAACTCCACGAAACGTTTACCCATCTGGACCAGAACCATCTACACCAGGAGGAAAAGTAGCATCGCATAAGCCT
Minc_76_14   AAGGACCAGCATCTACTCCAAAACAACCTGGATATCCTCAAGGACGAGCATCACCAACTCCACGAAACGTTTACCCATCTGGACCAGAACCATCTACACCAGGAGGAAAAGTAGCATCGCATAAGCCT
Minc_76_3    AAGGACCAGCATCTACTCCAAAACAACCTGGATATCCTCAAGGACGAGCATCACCAACTCCACGAAACGTTTACCCATCTGGACCAGAACCATCTACACCAGGAGGAAAAGTAGCATCGCATAAGCCT
Minc_76_7    AAGGACCAGCATCTACTCCAAAACAACCTGGATATCCTCAAGGACGAGCATCACCAACTCCACGAAACGTTTACCCATCTGGACCAGAACCATCTACACCAGGAGGAAAAGTAGCATCGCATAAGCCT
Minc_76_8    AAGGACCAGCATCTACTCCAAAACAACCTGGATATCCTCAAGGACGAGCATCACCAACTCCACGAAACGTTTACCCATCTGGACCAGAACCATCTACACCAGGAGGAAAAGTAGCATCGCATAAGCCT
Minc_76_9    AAGGACCAGCATCTACTCCAAAACAACCTGGATATCCTCAAGGACGAGCATCACCAACTCCACGAAACGTTTACCCATCTGGACCAGAACCATCTACACCAGGAGGAAAAGTAGCATCGCATAAGCCT
Minc_78_10   AAGGACCAGCATCTACTCCAAAACAACCTGGATATCCTCAAGGACGAGCATCACCAACTCCACGAAACGTTTACCCATCTGGACCAGAACCATCTACACCAGGAGGAAAAGTAGCATCGCATAAGCCT
Minc_78_11   AAGGACCAGCATCTACTCCAAAACAACCTGGATATCCTCAAGGACGAGCATCACCAACTCCACGAAACGTTTACCCATCTGGACCAGAACCATCTACACCAGGAGGAAAAGTAGCATCGCATAAGCCT
Minc_78_13   AAGGACCAGCATCTACTCCAAAACAACCTGGATATCCTCAAGGACGAGCATCACCAACTCCACGAAACGTTTACCCATCTGGACCAGAACCATCTACACCAGGAGGAAAAGTAGCATCGCATAAGCCT
Minc_78_14   AAGGACCAGCATCTACTCCAAAACAACCTGGATATCCTCAAGGACGAGCATCACCAACTCCACGAAACGTTTACCCATCTGGACCAGAACCATCTACACCAGGAGGAAAAGTAGCATCGCATAAGCCT
Minc_78_15   AAGGACCAGCATCTACTCCAAAACAACCTGGATATCCTCAAGGACGAGCATCACCAACTCCACGAAACGTTTACCCATCTGGACCAGAACCATCTACACCAGGAGGAAAAGTAGCATCGCATAAGCCT
Minc_78_16   AAGGACCAGCATCTACTCCAAAACAACCTGGATATCCTCAAGGACGAGCATCACCAACTCCACGAAACGTTTACCCATCTGGACCAGAACCATCTACACCAGGAGGAAAAGTAGCATCGCATAAGCCT
Minc_78_17   AAGGACCAGCATCTACTCCAAAACAACCTGGATATCCTCAAGGACGAGCATCACCAACTCCACGAAACGTTTACCCATCTGGACCAGAACCATCTACACCAGGAGGAAAAGTAGCATCGCATAAGCCT
Minc_78_7    AAGGACCAGCATCTACTCCAAAACAACCTGGATATCCTCAAGGACGAGCATCACCAACTCCACGAAACGTTTACCCATCTGGACCAGAACCATCTACACCAGGAGGAAAAGTAGCATCGCATAAGCCT
Minc_81_12   AAGGACCAGCATCTACTCCAAAACAACCTGGATATCCTCAAGGACGAGCATCACCAACTCCACGAAACGTTTACCCATCTGGACCAGAACCATCTACACCAGGAGGAAAAGTAGCATCGCATAAGCCT
Minc_81_13   AAGGACCAGCATCTACTCCAAAACAACCTGGATATCCTCAAGGACGAGCATCACCAACTCCACGAAACGTTTACCCATCTGGACCAGAACCATCTACACCAGGAGGAAAAGTAGCATCGCATAAGCCT
Minc_81_14   AAGGACCAGCATCTACTCCAAAACAACCTGGATATCCTCAAGGACGAGCATCACCAACTCCACGAAACGTTTACCCATCTGGACCAGAACCATCTACACCAGGAGGAAAAGTAGCATCGCATAAGCCT
Minc_81_15   AAGGACCAGCATCTACTCCAAAACAACCTGGATATCCTCAAGGACGAGCATCACCAACTCCACGAAACGTTTACCCATCTGGACCAGAACCATCTACACCAGGAGGAAAAGTAGCATCGCATAAGCCT
Minc_81_2    AAGGACCAGCATCTACTCCAAAACAACCTGGATATCCTCAAGGACGAGCATCACCAACTCCACGAAACGTTTACCCATCTGGACCAGAACCATCTACACCAGGAGGAAAAGTAGCATCGCATAAGCCT
Minc_81_4    AAGGACCAGCATCTACTCCAAAACAACCTGGATATCCTCAAGGACGAGCATCACCAACTCCACGAAACGTTTACCCATCTGGACCAGAACCATCTACACCAGGAGGAAAAGTAGCATCGCATAAGCCT
Minc_81_6    AAGGACCAGCATCTACTCCAAAACAACCTGGATATCCTCAAGGACGAGCATCACCAACTCCACGAAACGTTTACCCATCTGGACCAGAACCATCTACACCAGGAGGAAAAGTAGCATCGCATAAGCCT
Minc_81_8    AAGGACCAGCATCTACTCCAAAACAACCTGGATATCCTCAAGGACGAGCATCACCAACTCCACGAAACGTTTACCCATCTGGACCAGAACCATCTACACCAGGAGGAAAAGTAGCATCGCATAAGCCT
             ********************************************************************************************************************************

             CCTGCAGCACCTTCTACCCCACAGCCTGGATATCCTCAACCACCCGCAGCTCCAACAAAACCTCCACGAAACTTATATCCATCTGGACCGGAACCTACTCCAAAACAACCTGGATATCCCCAAGGACC
Minc_18_1    CCTGCAGCACCTTCTACCCCACAGCCTGGATATCCTCAACCACCCGCAGCTCCAACAAAACCTCCACGAAACTTATATCCATCTGGACCGGAACCTACTCCAAAACAACCTGGATATCCCCAAGGACC
Minc_18_10   CCTGCAGCACCTTCTACCCCACAGCCTGGATATCCTCAACCACCCGCAGCTCCAACAAAACCTCCACGAAACTTATATCCATCTGGACCGGAACCTACTCCAAAACAACCTGGATATCCCCAAGGACC
Minc_18_11   CCTGCAGCACCTTCTACCCCACAGCCTGGATATCCTCAACCACCCGCAGCTCCAACAAAACCTCCACGAAACTTATATCCATCTGGACCGGAACCTACTCCAAAACAACCTGGATATCCCCAAGGACC
Minc_18_13   CCTGCAGCACCTTCTACCCCACAGCCTGGATATCCTCAACCACCCGCAGCTCCAACAAAACCTCCACGAAACTTATATCCATCTGGACCGGAACCTACTCCAAAACAACCTGGATATCCCCAAGGACC
Minc_18_3    CCTGCAGCACCTTCTACCCCACAGCCTGGATATCCTCAACCACCCGCAGCTCCAACAAAACCTCCACGAAACTTATATCCATCTGGACCGGAACCTACTCCAAAACAACCTGGATATCCCCAAGGACC
Minc_18_4    CCTGCAGCACCTTCTACCCCACAGCCTGGATATCCTCAACCACCCGCAGCTCCAACAAAACCTCCACGAAACTTATATCCATCTGGACCGGAACCTACTCCAAAACAACCTGGATATCCCCAAGGACC
Minc_18_5    CCTGCAGCACCTTCTACCCCACAGCCTGGATATCCTCAACCACCCGCAGCTCCAACAAAACCTCCACGAAACTTATATCCATCTGGACCGGAACCTACTCCAAAACAACCTGGATATCCCCAAGGACC
Minc_18_7    CCTGCAGCACCTTCTACCCCACAGCCTGGATATCCTCAACCACCCGCAGCTCCAACAAAACCTCCACGAAACTTATATCCATCTGGACCGGAACCTACTCCAAAACAACCTGGATATCCCCAAGGACC
Minc_18_8    CCTGCAGCACCTTCTACCCCACAGCCTGGATATCCTCAACCACCCGCAGCTCCAACAAAACCTCCACGAAACTTATATCCATCTGGACCGGAACCTACTCCAAAACAACCTGGATATCCCCAAGGACC
Minc_18_9    CCTGCAGCACCTTCTACCCCACAGCCTGGATATCCTCAACCACCCGCAGCTCCAACAAAACCTCCACGAAACTTATATCCATCTGGACCGGAACCTACTCCAAAACAACCTGGATATCCCCAAGGACC
Minc_22_14   CCTGCAGCACCTTCTACCCCACAGCCTGGATATCCTCAACCACCCGCAGCTCCAACAAAACCTCCACGAAACTTATATCCATCTGGACCGGAACCTACTCCAAAACAACCTGGATATCCCCAAGGACC
Minc_22_15   CCTGCAGCACCTTCTACCCCACAGCCTGGATATCCTCAACCACCCGCAGCTCCAACAAAACCTCCACGAAACTTATATCCATCTGGACCGGAACCTACTCCAAAACAACCTGGATATCCCCAAGGACC
Minc_22_2    CCTGCAGCACCTTCTACCCCACAGCCTGGATATCCTCAACCACCCGCAGCTCCAACAAAACCTCCACGAAACTTATATCCATCTGGACCGGAACCTACTCCAAAACAACCTGGATATCCCCAAGGACC
Minc_22_3    CCTGCAGCACCTTCTACCCCACAGCCTGGATATCCTCAACCACCCGCAGCTCCAACAAAACCTCCACGAAACTTATATCCATCTGGACCGGAACCTACTCCAAAACAACCTGGATATCCCCAAGGACC
Minc_22_5    CCTGCAGCACCTTCTACCCCACAGCCTGGATATCCTCAACCACCCGCAGCTCCAACAAAACCTCCACGAAACTTATATCCATCTGGACCGGAACCTACTCCAAAACAACCTGGATATCCCCAAGGACC
Minc_22_7    CCTGCAGCACCTTCTACCCCACAGCCTGGATATCCTCAACCACCCGCAGCTCCAACAAAACCTCCACGAAACTTATATCCATCTGGACCGGAACCTACTCCAAAACAACCTGGATATCCCCAAGGACC
Minc_22_8    CCTGCAGCACCTTCTACCCCACAGCCTGGATATCCTCAACCACCCGCAGCTCCAACAAAACCTCCACGAAACTTATATCCATCTGGACCGGAACCTACTCCAAAACAACCTGGATATCCCCAAGGACC
Minc_30_1    CCTGCAGCACCTTCTACCCCACAGCCTGGATATCCTCAACCACCCGCAGCTCCAACAAAACCTCCACGAAACTTATATCCATCTGGACCGGAACCTACTCCAAAACAACCTGGATATCCCCAAGGACC
Minc_30_10   CCTGCAGCACCTTCTACCCCACAGCCTGGATATCCTCAACCACCCGCAGCTCCAACAAAACCTCCACGAAACTTATATCCATCTGGACCGGAACCTACTCCAAAACAACCTGGATATCCCCAAGGACC
Minc_30_11   CCTGCAGCACCTTCTACCCCACAGCCTGGATATCCTCAACCACCCGCAGCTCCAACAAAACCTCCACGAAACTTATATCCATCTGGACCGGAACCTACTCCAAAACAACCTGGATATCCCCAAGGACC
Minc_30_4    CCTGCAGCACCTTCTACCCCACAGCCTGGATATCCTCAACCACCCGCAGCTCCAACAAAACCTCCACGAAACTTATATCCATCTGGACCGGAACCTACTCCAAAACAACCTGGATATCCCCAAGGACC
Minc_30_5    CCTGCAGCACCTTCTACCCCACAGCCTGGATATCCTCAACCACCCGCAGCTCCAACAAAACCTCCACGAAACTTATATCCATCTGGACCGGAACCTACTCCAAAACAACCTGGATATCCCCAAGGACC
Minc_34_1    CCTGCAGCACCTTCTACCCCACAGCCTGGATATCCTCAACCACCCGCAGCTCCAACAAAACCTCCACGAAACTTATATCCATCTGGACCGGAACCTACTCCAAAACAACCTGGATATCCCCAAGGACC
Minc_34_10   CCTGCAGCACCTTCTACCCCACAGCCTGGATATCCTCAACCACCCGCAGCTCCAACAAAACCTCCACGAAACTTATATCCATCTGGACCGGAACCTACTCCAAAACAACCTGGATATCCCCAAGGACC
Minc_34_11   CCTGCAGCACCTTCTACCCCACAGCCTGGATATCCTCAACCACCCGCAGCTCCAACAAAACCTCCACGAAACTTATATCCATCTGGACCGGAACCTACTCCAAAACAACCTGGATATCCCCAAGGACC
Minc_34_12   CCTGCAGCACCTTCTACCCCACAGCCTGGATATCCTCAACCACCCGCAGCTCCAACAAAACCTCCACGAAACTTATATCCATCTGGACCGGAACCTACTCCAAAACAACCTGGATATCCCCAAGGACC
Minc_34_3    CCTGCAGCACCTTCTACCCCACAGCCTGGATATCCTCAACCACCCGCAGCTCCAACAAAACCTCCACGAAACTTATATCCATCTGGACCGGAACCTACTCCAAAACAACCTGGATATCCCCAAGGACC
Minc_34_5    CCTGCAGCACCTTCTACCCCACAGCCTGGATATCCTCAACCACCCGCAGCTCCAACAAAACCTCCACGAAACTTATATCCATCTGGACCGGAACCTACTCCAAAACAACCTGGATATCCCCAAGGACC
Minc_34_7    CCTGCAGCACCTTCTACCCCACAGCCTGGATATCCTCAACCACCCGCAGCTCCAACAAAACCTCCACGAAACTTATATCCATCTGGACCGGAACCTACTCCAAAACAACCTGGATATCCCCAAGGACC
Minc_34_9    CCTGCAGCACCTTCTACCCCACAGCCTGGATATCCTCAACCACCCGCAGCTCCAACAAAACCTCCACGAAACTTATATCCATCTGGACCGGAACCTACTCCAAAACAACCTGGATATCCCCAAGGACC
Minc_37_10   CCTGCAGCACCTTCTACCCCACAGCCTGGATATCCTCAACCACCCGCAGCTCCAACAAAACCTCCACGAAACTTATATCCATCTGGACCGGAACCTACTCCAAAACAACCTGGATATCCCCAAGGACC
Minc_37_12   CCTGCAGCACCTTCTACCCCACAGCCTGGATATCCTCAACCACCCGCAGCTCCAACAAAACCTCCACGAAACTTATATCCATCTGGACCGGAACCTACTCCAAAACAACCTGGATATCCCCAAGGACC
Minc_37_2    CCTGCAGCACCTTCTACCCCACAGCCTGGATATCCTCAACCACCCGCAGCTCCAACAAAACCTCCACGAAACTTATATCCATCTGGACCGGAACCTACTCCAAAACAACCTGGATATCCCCAAGGACC
Minc_37_3    CCTGCAGCACCTTCTACCCCACAGCCTGGATATCCTCAACCACCCGCAGCTCCAACAAAACCTCCACGAAACTTATATCCATCTGGACCGGAACCTACTCCAAAACAACCTGGATATCCCCAAGGACC
Minc_37_4    CCTGCAGCACCTTCTACCCCACAGCCTGGATATCCTCAACCACCCGCAGCTCCAACAAAACCTCCACGAAACTTATATCCATCTGGACCGGAACCTACTCCAAAACAACCTGGATATCCCCAAGGACC
Minc_37_5    CCTGCAGCACCTTCTACCCCACAGCCTGGATATCCTCAACCACCCGCAGCTCCAACAAAACCTCCACGAAACTTATATCCATCTGGACCGGAACCTACTCCAAAACAACCTGGATATCCCCAAGGACC
Minc_37_6    CCTGCAGCACCTTCTACCCCACAGCCTGGATATCCTCAACCACCCGCAGCTCCAACAAAACCTCCACGAAACTTATATCCATCTGGACCGGAACCTACTCCAAAACAACCTGGATATCCCCAAGGACC
Minc_37_8    CCTGCAGCACCTTCTACCCCACAGCCTGGATATCCTCAACCACCCGCAGCTCCAACAAAACCTCCACGAAACTTATATCCATCTGGACCGGAACCTACTCCAAAACAACCTGGATATCCCCAAGGACC
Minc_37_9    CCTGCAGCACCTTCTACCCCACAGCCTGGATATCCTCAACCACCCGCAGCTCCAACAAAACCTCCACGAAACTTATATCCATCTGGACCGGAACCTACTCCAAAACAACCTGGATATCCCCAAGGACC
Minc_42_1    CCTGCAGCACCTTCTACCCCACAGCCTGGATATCCTCAACCACCCGCAGCTCCAACAAAACCTCCACGAAACTTATATCCATCTGGACCGGAACCTACTCCAAAACAACCTGGATATCCCCAAGGACC
Minc_42_10   CCTGCAGCACCTTCTACCCCACAGCCTGGATATCCTCAACCACCCGCAGCTCCAACAAAACCTCCACGAAACTTATATCCATCTGGACCGGAACCTACTCCAAAACAACCTGGATATCCCCAAGGACC
Minc_42_11   CCTGCAGCACCTTCTACCCCACAGCCTGGATATCCTCAACCACCCGCAGCTCCAACAAAACCTCCACGAAACTTATATCCATCTGGACCGGAACCTACTCCAAAACAACCTGGATATCCCCAAGGACC
Minc_42_12   CCTGCAGCACCTTCTACCCCACAGCCTGGATATCCTCAACCACCCGCAGCTCCAACAAAACCTCCACGAAACTTATATCCATCTGGACCGGAACCTACTCCAAAACAACCTGGATATCCCCAAGGACC
Minc_42_3    CCTGCAGCACCTTCTACCCCACAGCCTGGATATCCTCAACCACCCGCAGCTCCAACAAAACCTCCACGAAACTTATATCCATCTGGACCGGAACCTACTCCAAAACAACCTGGATATCCCCAAGGACC
Minc_42_4    CCTGCAGCACCTTCTACCCCACAGCCTGGATATCCTCAACCACCCGCAGCTCCAACAAAACCTCCACGAAACTTATATCCATCTGGACCGGAACCTACTCCAAAACAACCTGGATATCCCCAAGGACC
Minc_42_5    CCTGCAGCACCTTCTACCCCACAGCCTGGATATCCTCAACCACCCGCAGCTCCAACAAAACCTCCACGAAACTTATATCCATCTGGACCGGAACCTACTCCAAAACAACCTGGATATCCCCAAGGACC
Minc_42_7    CCTGCAGCACCTTCTACCCCACAGCCTGGATATCCTCAACCACCCGCAGCTCCAACAAAACCTCCACGAAACTTATATCCATCTGGACCGGAACCTACTCCAAAACAACCTGGATATCCCCAAGGACC
Minc_42_8    CCTGCAGCACCTTCTACCCCACAGCCTGGATATCCTCAACCACCCGCAGCTCCAACAAAACCTCCACGAAACTTATATCCATCTGGACCGGAACCTACTCCAAAACAACCTGGATATCCCCAAGGACC
Minc_42_9    CCTGCAGCACCTTCTACCCCACAGCCTGGATATCCTCAACCACCCGCAGCTCCAACAAAACCTCCACGAAACTTATATCCATCTGGACCGGAACCTACTCCAAAACAACCTGGATATCCCCAAGGACC
Minc_45_10   CCTGCAGCACCTTCTACCCCACAGCCTGGATATCCTCAACCACCCGCAGCTCCAACAAAACCTCCACGAAACTTATATCCATCTGGACCGGAACCTACTCCAAAACAACCTGGATATCCCCAAGGACC
Minc_45_11   CCTGCAGCACCTTCTACCCCACAGCCTGGATATCCTCAACCACCCGCAGCTCCAACAAAACCTCCACGAAACTTATATCCATCTGGACCGGAACCTACTCCAAAACAACCTGGATATCCCCAAGGACC
Minc_45_12   CCTGCAGCACCTTCTACCCCACAGCCTGGATATCCTCAACCACCCGCAGCTCCAACAAAACCTCCACGAAACTTATATCCATCTGGACCGGAACCTACTCCAAAACAACCTGGATATCCCCAAGGACC
Minc_45_13   CCTGCAGCACCTTCTACCCCACAGCCTGGATATCCTCAACCACCCGCAGCTCCAACAAAACCTCCACGAAACTTATATCCATCTGGACCGGAACCTACTCCAAAACAACCTGGATATCCCCAAGGACC
Minc_45_7    CCTGCAGCACCTTCTACCCCACAGCCTGGATATCCTCAACCACCCGCAGCTCCAACAAAACCTCCACGAAACTTATATCCATCTGGACCGGAACCTACTCCAAAACAACCTGGATATCCCCAAGGACC
Minc_45_9    CCTGCAGCACCTTCTACCCCACAGCCTGGATATCCTCAACCACCCGCAGCTCCAACAAAACCTCCACGAAACTTATATCCATCTGGACCGGAACCTACTCCAAAACAACCTGGATATCCCCAAGGACC
```

```
Minc_47_1     CCTGCAGCACCTTCTACCCCACAGCCTGGATATCCTCAACCACCCGCAGCTCCAACAAAACCTCCACGAAACTTATATCCATCTGGACCGGAACCTACTCCAAAACAACCTGGATATCCCCAAGGACC
Minc_47_10    CCTGCAGCACCTTCTACCCCACAGCCTGGATATCCTCAACCACCCGCAGCTCCAACAAAACCTCCACGAAACTTATATCCATCTGGACCGGAACCTACTCCAAAACAACCTGGATATCCCCAAGGACC
Minc_47_12    CCTGCAGCACCTTCTACCCCACAGCCTGGATATCCTCAACCACCCGCAGCTCCAACAAAACCTCCACGAAACTTATATCCATCTGGACCGGAACCTACTCCAAAACAACCTGGATATCCCCAAGGACC
Minc_47_13    CCTGCAGCACCTTCTACCCCACAGCCTGGATATCCTCAACCACCCGCAGCTCCAACAAAACCTCCACGAAACTTATATCCATCTGGACCGGAACCTACTCCAAAACAACCTGGATATCCCCAAGGACC
Minc_47_3     CCTGCAGCACCTTCTACCCCACAGCCTGGATATCCTCAACCACCCGCAGCTCCAACAAAACCTCCACGAAACTTATATCCATCTGGACCGGAACCTACTCCAAAACAACCTGGATATCCCCAAGGACC
Minc_47_7     CCTGCAGCACCTTCTACCCCACAGCCTGGATATCCTCAACCACCCGCAGCTCCAACAAAACCTCCACGAAACTTATATCCATCTGGACCGGAACCTACTCCAAAACAACCTGGATATCCCCAAGGACC
Minc_47_8     CCTGCAGCACCTTCTACCCCACAGCCTGGATATCCTCAACCACCCGCAGCTCCAACAAAACCTCCACGAAACTTATATCCATCTGGACCGGAACCTACTCCAAAACAACCTGGATATCCCCAAGGACC
Minc_51_10    CCTGCAGCACCTTCTACCCCACAGCCTGGATATCCTCAACCACCCGCAGCTCCAACAAAACCTCCACGAAACTTATATCCATCTGGACCGGAACCTACTCCAAAACAACCTGGATATCCCCAAGGACC
Minc_51_11    CCTGCAGCACCTTCTACCCCACAGCCTGGATATCCTCAACCACCCGCAGCTCCAACAAAACCTCCACGAAACTTATATCCATCTGGACCGGAACCTACTCCAAAACAACCTGGATATCCCCAAGGACC
Minc_51_4     CCTGCAGCACCTTCTACCCCACAGCCTGGATATCCTCAACCACCCGCAGCTCCAACAAAACCTCCACGAAACTTATATCCATCTGGACCGGAACCTACTCCAAAACAACCTGGATATCCCCAAGGACC
Minc_51_6     CCTGCAGCACCTTCTACCCCACAGCCTGGATATCCTCAACCACCCGCAGCTCCAACAAAACCTCCACGAAACTTATATCCATCTGGACCGGAACCTACTCCAAAACAACCTGGATATCCCCAAGGACC
Minc_51_7     CCTGCAGCACCTTCTACCCCACAGCCTGGATATCCTCAACCACCCGCAGCTCCAACAAAACCTCCACGAAACTTATATCCATCTGGACCGGAACCTACTCCAAAACAACCTGGATATCCCCAAGGACC
Minc_54_1     CCTGCAGCACCTTCTACCCCACAGCCTGGATATCCTCAACCACCCGCAGCTCCAACAAAACCTCCACGAAACTTATATCCATCTGGACCGGAACCTACTCCAAAACAACCTGGATATCCCCAAGGACC
Minc_54_10    CCTGCAGCACCTTCTACCCCACAGCCTGGATATCCTCAACCACCCGCAGCTCCAACAAAACCTCCACGAAACTTATATCCATCTGGACCGGAACCTACTCCAAAACAACCTGGATATCCCCAAGGACC
Minc_54_3     CCTGCAGCACCTTCTACCCCACAGCCTGGATATCCTCAACCACCCGCAGCTCCAACAAAACCTCCACGAAACTTATATCCATCTGGACCGGAACCTACTCCAAAACAACCTGGATATCCCCAAGGACC
Minc_54_4     CCTGCAGCACCTTCTACCCCACAGCCTGGATATCCTCAACCACCCGCAGCTCCAACAAAACCTCCACGAAACTTATATCCATCTGGACCGGAACCTACTCCAAAACAACCTGGATATCCCCAAGGACC
Minc_54_5     CCTGCAGCACCTTCTACCCCACAGCCTGGATATCCTCAACCACCCGCAGCTCCAACAAAACCTCCACGAAACTTATATCCATCTGGACCGGAACCTACTCCAAAACAACCTGGATATCCCCAAGGACC
Minc_54_6     CCTGCAGCACCTTCTACCCCACAGCCTGGATATCCTCAACCACCCGCAGCTCCAACAAAACCTCCACGAAACTTATATCCATCTGGACCGGAACCTACTCCAAAACAACCTGGATATCCCCAAGGACC
Minc_56_1     CCTGCAGCACCTTCTACCCCACAGCCTGGATATCCTCAACCACCCGCAGCTCCAACAAAACCTCCACGAAACTTATATCCATCTGGACCGGAACCTACTCCAAAACAACCTGGATATCCCCAAGGACC
Minc_56_12    CCTGCAGCACCTTCTACCCCACAGCCTGGATATCCTCAACCACCCGCAGCTCCAACAAAACCTCCACGAAACTTATATCCATCTGGACCGGAACCTACTCCAAAACAACCTGGATATCCCCAAGGACC
Minc_56_2     CCTGCAGCACCTTCTACCCCACAGCCTGGATATCCTCAACCACCCGCAGCTCCAACAAAACCTCCACGAAACTTATATCCATCTGGACCGGAACCTACTCCAAAACAACCTGGATATCCCCAAGGACC
Minc_56_3     CCTGCAGCACCTTCTACCCCACAGCCTGGATATCCTCAACCACCCGCAGCTCCAACAAAACCTCCACGAAACTTATATCCATCTGGACCGGAACCTACTCCAAAACAACCTGGATATCCCCAAGGACC
Minc_56_4     CCTGCAGCACCTTCTACCCCACAGCCTGGATATCCTCAACCACCCGCAGCTCCAACAAAACCTCCACGAAACTTATATCCATCTGGACCGGAACCTACTCCAAAACAACCTGGATATCCCCAAGGACC
Minc_56_5     CCTGCAGCACCTTCTACCCCACAGCCTGGATATCCTCAACCACCCGCAGCTCCAACAAAACCTCCACGAAACTTATATCCATCTGGACCGGAACCTACTCCAAAACAACCTGGATATCCCCAAGGACC
Minc_56_6     CCTGCAGCACCTTCTACCCCACAGCCTGGATATCCTCAACCACCCGCAGCTCCAACAAAACCTCCACGAAACTTATATCCATCTGGACCGGAACCTACTCCAAAACAACCTGGATATCCCCAAGGACC
Minc_56_7     CCTGCAGCACCTTCTACCCCACAGCCTGGATATCCTCAACCACCCGCAGCTCCAACAAAACCTCCACGAAACTTATATCCATCTGGACCGGAACCTACTCCAAAACAACCTGGATATCCCCAAGGACC
Minc_58_1     CCTGCAGCACCTTCTACCCCACAGCCTGGATATCCTCAACCACCCGCAGCTCCAACAAAACCTCCACGAAACTTATATCCATCTGGACCGGAACCTACTCCAAAACAACCTGGATATCCCCAAGGACC
Minc_58_13    CCTGCAGCACCTTCTACCCCACAGCCTGGATATCCTCAACCACCCGCAGCTCCAACAAAACCTCCACGAAACTTATATCCATCTGGACCGGAACCTACTCCAAAACAACCTGGATATCCCCAAGGACC
Minc_58_2     CCTGCAGCACCTTCTACCCCACAGCCTGGATATCCTCAACCACCCGCAGCTCCAACAAAACCTCCACGAAACTTATATCCATCTGGACCGGAACCTACTCCAAAACAACCTGGATATCCCCAAGGACC
Minc_58_3     CCTGCAGCACCTTCTACCCCACAGCCTGGATATCCTCAACCACCCGCAGCTCCAACAAAACCTCCACGAAACTTATATCCATCTGGACCGGAACCTACTCCAAAACAACCTGGATATCCCCAAGGACC
Minc_58_4     CCTGCAGCACCTTCTACCCCACAGCCTGGATATCCTCAACCACCCGCAGCTCCAACAAAACCTCCACGAAACTTATATCCATCTGGACCGGAACCTACTCCAAAACAACCTGGATATCCCCAAGGACC
Minc_58_5     CCTGCAGCACCTTCTACCCCACAGCCTGGATATCCTCAACCACCCGCAGCTCCAACAAAACCTCCACGAAACTTATATCCATCTGGACCGGAACCTACTCCAAAACAACCTGGATATCCCCAAGGACC
Minc_58_6     CCTGCAGCACCTTCTACCCCACAGCCTGGATATCCTCAACCACCCGCAGCTCCAACAAAACCTCCACGAAACTTATATCCATCTGGACCGGAACCTACTCCAAAACAACCTGGATATCCCCAAGGACC
Minc_58_7     CCTGCAGCACCTTCTACCCCACAGCCTGGATATCCTCAACCACCCGCAGCTCCAACAAAACCTCCACGAAACTTATATCCATCTGGACCGGAACCTACTCCAAAACAACCTGGATATCCCCAAGGACC
Minc_58_8     CCTGCAGCACCTTCTACCCCACAGCCTGGATATCCTCAACCACCCGCAGCTCCAACAAAACCTCCACGAAACTTATATCCATCTGGACCGGAACCTACTCCAAAACAACCTGGATATCCCCAAGGACC
Minc_58_9     CCTGCAGCACCTTCTACCCCACAGCCTGGATATCCTCAACCACCCGCAGCTCCAACAAAACCTCCACGAAACTTATATCCATCTGGACCGGAACCTACTCCAAAACAACCTGGATATCCCCAAGGACC
Minc_65_1     CCTGCAGCACCTTCTACCCCACAGCCTGGATATCCTCAACCACCCGCAGCTCCAACAAAACCTCCACGAAACTTATATCCATCTGGACCGGAACCTACTCCAAAACAACCTGGATATCCCCAAGGACC
Minc_65_11    CCTGCAGCACCTTCTACCCCACAGCCTGGATATCCTCAACCACCCGCAGCTCCAACAAAACCTCCACGAAACTTATATCCATCTGGACCGGAACCTACTCCAAAACAACCTGGATATCCCCAAGGACC
Minc_65_3     CCTGCAGCACCTTCTACCCCACAGCCTGGATATCCTCAACCACCCGCAGCTCCAACAAAACCTCCACGAAACTTATATCCATCTGGACCGGAACCTACTCCAAAACAACCTGGATATCCCCAAGGACC
Minc_65_6     CCTGCAGCACCTTCTACCCCACAGCCTGGATATCCTCAACCACCCGCAGCTCCAACAAAACCTCCACGAAACTTATATCCATCTGGACCGGAACCTACTCCAAAACAACCTGGATATCCCCAAGGACC
Minc_65_8     CCTGCAGCACCTTCTACCCCACAGCCTGGATATCCTCAACCACCCGCAGCTCCAACAAAACCTCCACGAAACTTATATCCATCTGGACCGGAACCTACTCCAAAACAACCTGGATATCCCCAAGGACC
Minc_65_9     CCTGCAGCACCTTCTACCCCACAGCCTGGATATCCTCAACCACCCGCAGCTCCAACAAAACCTCCACGAAACTTATATCCATCTGGACCGGAACCTACTCCAAAACAACCTGGATATCCCCAAGGACC
Minc_76_1     CCTGCAGCACCTTCTACCCCACAGCCTGGATATCCTCAACCACCCGCAGCTCCAACAAAACCTCCACGAAACTTATATCCATCTGGACCGGAACCTACTCCAAAACAACCTGGATATCCCCAAGGACC
Minc_76_10    CCTGCAGCACCTTCTACCCCACAGCCTGGATATCCTCAACCACCCGCAGCTCCAACAAAACCTCCACGAAACTTATATCCATCTGGACCGGAACCTACTCCAAAACAACCTGGATATCCCCAAGGACC
Minc_76_12    CCTGCAGCACCTTCTACCCCACAGCCTGGATATCCTCAACCACCCGCAGCTCCAACAAAACCTCCACGAAACTTATATCCATCTGGACCGGAACCTACTCCAAAACAACCTGGATATCCCCAAGGACC
Minc_76_13    CCTGCAGCACCTTCTACCCCACAGCCTGGATATCCTCAACCACCCGCAGCTCCAACAAAACCTCCACGAAACTTATATCCATCTGGACCGGAACCTACTCCAAAACAACCTGGATATCCCCAAGGACC
Minc_76_14    CCTGCAGCACCTTCTACCCCACAGCCTGGATATCCTCAACCACCCGCAGCTCCAACAAAACCTCCACGAAACTTATATCCATCTGGACCGGAACCTACTCCAAAACAACCTGGATATCCCCAAGGACC
Minc_76_3     CCTGCAGCACCTTCTACCCCACAGCCTGGATATCCTCAACCACCCGCAGCTCCAACAAAACCTCCACGAAACTTATATCCATCTGGACCGGAACCTACTCCAAAACAACCTGGATATCCCCAAGGACC
Minc_76_7     CCTGCAGCACCTTCTACCCCACAGCCTGGATATCCTCAACCACCCGCAGCTCCAACAAAACCTCCACGAAACTTATATCCATCTGGACCGGAACCTACTCCAAAACAACCTGGATATCCCCAAGGACC
Minc_76_8     CCTGCAGCACCTTCTACCCCACAGCCTGGATATCCTCAACCACCCGCAGCTCCAACAAAACCTCCACGAAACTTATATCCATCTGGACCGGAACCTACTCCAAAACAACCTGGATATCCCCAAGGACC
Minc_76_9     CCTGCAGCACCTTCTACCCCACAGCCTGGATATCCTCAACCACCCGCAGCTCCAACAAAACCTCCACGAAACTTATATCCATCTGGACCGGAACCTACTCCAAAACAACCTGGATATCCCCAAGGACC
Minc_78_10    CCTGCAGCACCTTCTACCCCACAGCCTGGATATCCTCAACCACCCGCAGCTCCAACAAAACCTCCACGAAACTTATATCCATCTGGACCGGAACCTACTCCAAAACAACCTGGATATCCCCAAGGACC
Minc_78_11    CCTGCAGCACCTTCTACCCCACAGCCTGGATATCCTCAACCACCCGCAGCTCCAACAAAACCTCCACGAAACTTATATCCATCTGGACCGGAACCTACTCCAAAACAACCTGGATATCCCCAAGGACC
Minc_78_13    CCTGCAGCACCTTCTACCCCACAGCCTGGATATCCTCAACCACCCGCAGCTCCAACAAAACCTCCACGAAACTTATATCCATCTGGACCGGAACCTACTCCAAAACAACCTGGATATCCCCAAGGACC
Minc_78_14    CCTGCAGCACCTTCTACCCCACAGCCTGGATATCCTCAACCACCCGCAGCTCCAACAAAACCTCCACGAAACTTATATCCATCTGGACCGGAACCTACTCCAAAACAACCTGGATATCCCCAAGGACC
Minc_78_15    CCTGCAGCACCTTCTACCCCACAGCCTGGATATCCTCAACCACCCGCAGCTCCAACAAAACCTCCACGAAACTTATATCCATCTGGACCGGAACCTACTCCAAAACAACCTGGATATCCCCAAGGACC
Minc_78_16    CCTGCAGCACCTTCTACCCCACAGCCTGGATATCCTCAACCACCCGCAGCTCCAACAAAACCTCCACGAAACTTATATCCATCTGGACCGGAACCTACTCCAAAACAACCTGGATATCCCCAAGGACC
Minc_78_17    CCTGCAGCACCTTCTACCCCACAGCCTGGATATCCTCAACCACCCGCAGCTCCAACAAAACCTCCACGAAACTTATATCCATCTGGACCGGAACCTACTCCAAAACAACCTGGATATCCCCAAGGACC
Minc_78_7     CCTGCAGCACCTTCTACCCCACAGCCTGGATATCCTCAACCACCCGCAGCTCCAACAAAACCTCCACGAAACTTATATCCATCTGGACCGGAACCTACTCCAAAACAACCTGGATATCCCCAAGGACC
Minc_81_12    CCTGCAGCACCTTCTACCCCACAGCCTGGATATCCTCAACCACCCGCAGCTCCAACAAAACCTCCACGAAACTTATATCCATCTGGACCGGAACCTACTCCAAAACAACCTGGATATCCCCAAGGACC
Minc_81_13    CCTGCAGCACCTTCTACCCCACAGCCTGGATATCCTCAACCACCCGCAGCTCCAACAAAACCTCCACGAAACTTATATCCATCTGGACCGGAACCTACTCCAAAACAACCTGGATATCCCCAAGGACC
Minc_81_14    CCTGCAGCACCTTCTACCCCACAGCCTGGATATCCTCAACCACCCGCAGCTCCAACAAAACCTCCACGAAACTTATATCCATCTGGACCGGAACCTACTCCAAAACAACCTGGATATCCCCAAGGACC
Minc_81_15    CCTGCAGCACCTTCTACCCCACAGCCTGGATATCCTCAACCACCCGCAGCTCCAACAAAACCTCCACGAAACTTATATCCATCTGGACCGGAACCTACTCCAAAACAACCTGGATATCCCCAAGGACC
Minc_81_2     CCTGCAGCACCTTCTACCCCACAGCCTGGATATCCTCAACCACCCGCAGCTCCAACAAAACCTCCACGAAACTTATATCCATCTGGACCGGAACCTACTCCAAAACAACCTGGATATCCCCAAGGACC
Minc_81_4     CCTGCAGCACCTTCTACCCCACAGCCTGGATATCCTCAACCACCCGCAGCTCCAACAAAACCTCCACGAAACTTATATCCATCTGGACCGGAACCTACTCCAAAACAACCTGGATATCCCCAAGGACC
Minc_81_6     CCTGCAGCACCTTCTACCCCACAGCCTGGATATCCTCAACCACCCGCAGCTCCAACAAAACCTCCACGAAACTTATATCCATCTGGACCGGAACCTACTCCAAAACAACCTGGATATCCCCAAGGACC
Minc_81_8     CCTGCAGCACCTTCTACCCCACAGCCTGGATATCCTCAACCACCCGCAGCTCCAACAAAACCTCCACGAAACTTATATCCATCTGGACCGGAACCTACTCCAAAACAACCTGGATATCCCCAAGGACC
              ********************************************************************************************************************************
```

```
              AGCATCA
Minc_18_1     AGCATCACCAACTCCACCAAGCCACAACCAACCAAAACAGCCAAGTCCTACTCCACCAAGCTCAGCTCCTTCTCCACCAGG
Minc_18_10    AGCATCACCAACTCCACCAAGCCACAACCAACCAAAACAGCCAAGTCCTACTCCACCAAGCTCAGCTCCTTCTCCACCAGG
Minc_18_11    AGCATCACCAACTCCACCAAGCCACAACCAACCAAAACAGCCAAGTCCTACTCCACCAAGCTCAGCTCCTTCTCCACCAGG
Minc_18_13    AGCATCACCAACTCCACCAAGCCACAACCAACCAAAACAGCCAAGTCCTACTCCACCAAGCTCAGCTCCTTCTCCACCAGG
Minc_18_3     AGCATCACCAACTCCACCAAGCCACAACCAACCAAAACAGCCAAGTCCTACTCCACCAAGCTCAGCTCCTTCTCCACCAGG
Minc_18_4     AGCATCACCAACTCCACCAAGCCACAACCAACCAAAACAGCCAAGTCCTACTCCACCAAGCTCAGCTCCTTCTCCACCAGG
Minc_18_5     AGCATCACCAACTCCACCAAGCCACAACCAACCAAAACAGCCAAGTCCTACTCCACCAAGCTCAGCTCCTTCTCCACCAGG
Minc_18_7     AGCATCACCAACTCCACCAAGCCACAACCAACCAAAACAGCCAAGTCCTACTCCACCAAGCTCAGCTCCTTCTCCACCAGG
Minc_18_8     AGCATCACCAACTCCACCAAGCCACAACCAACCAAAACAGCCAAGTCCTACTCCACCAAGCTCAGCTCCTTCTCCACCAGG
Minc_18_9     AGCATCACCAACTCCACCAAGCCACAACCAACCAAAACAGCCAAGTCCTACTCCACCAAGCTCAGCTCCTTCTCCACCAGG
Minc_22_14    AGCATCACCAACTCCACCAAGCCACAACCAACCAAAACAGCCAAGTCCTACTCCACCAAGCTCAGCTCCTTCTCCACCAGG
Minc_22_15    AGCATCACCAACTCCACCAAGCCACAACCAACCAAAACAGCCAAGTCCTACTCCACCAAGCTCAGCTCCTTCTCCACCAGG
Minc_22_2     AGCATCACCAACTCCACCAAGCCACAACCAACCAAAACAGCCAAGTCCTACTCCACCAAGCTCAGCTCCTTCTCCACCAGG
Minc_22_3     AGCATCACCAACTCCACCAAGCCACAACCAACCAAAACAGCCAAGTCCTACTCCACCAAGCTCAGCTCCTTCTCCACCAGG
Minc_22_5     AGCATCACCAACTCCACCAAGCCACAACCAACCAAAACAGCCAAGTCCTACTCCACCAAGCTCAGCTCCTTCTCCACCAGG
Minc_22_7     AGCATCACCAACTCCACCAAGCCACAACCAACCAAAACAGCCAAGTCCTACTCCACCAAGCTCAGCTCCTTCTCCACCAGG
Minc_22_8     AGCATCACCAACTCCACCAAGCCACAACCAACCAAAACAGCCAAGTCCTACTCCACCAAGCTCAGCTCCTTCTCCACCAGG
Minc_30_1     AGCATCACCAACTCCACCAAGCCACAACCAACCAAAACAGCCAAGTCCTACTCCACCAAGCTCAGCTCCTTCTCCACCAGG
Minc_30_10    AGCATCACCAACTCCACCAAGCCACAACCAACCAAAACAGCCAAGTCCTACTCCACCAAGCTCAGCTCCTTCTCCACCAGG
Minc_30_11    AGCATCACCAACTCCACCAAGCCACAACCAACCAAAACAGCCAAGTCCTACTCCACCAAGCTCAGCTCCTTCTCCACCAGG
Minc_30_4     AGCATCACCAACTCCACCAAGCCACAACCAACCAAAACAGCCAAGTCCTACTCCACCAAG-TCAGG-CCTTCTCCACCAGG
Minc_30_5     AGCATCACCAACTCCACCAAGCCACAACCAACCAAAACAGCCAAGTCCTACTCCACCAAGCTCAGCTCCTTCTCCACCAGG
Minc_34_1     AGCATCACCAACTCCACCAAGCCACAACCAACCAAAACAGCCAAGTCCTACTCCACCAAGCTCAGCTCCTTCTCCACCAGG
Minc_34_10    AGCATCACCAACTCCACCAAGCCACAACCAACCAAAACAGCCAAGTCCTACTCCACCAAGCTCAGCTCCTTCTCCACCAGG
Minc_34_11    AGCATCACCAACTCCACCAAGCCACAACCAACCAAAACAGCCAAGTCCTACTCCACCAAGCTCAGCTCCTTCTCCACCAGG
Minc_34_12    AGCATCACCAACTCCACCAAGCCACAACCAACCAAAACAGCCAAGTCCTACTCCACCAAGCTCAGCTCCTTCTCCACCAGG
Minc_34_3     AGCATCACCAACTCCACCAAGCCACAACCAACCAAAACAGCCAAGTCCTACTCCACCAAGCTCAGCTCCTTCTCCACCAGG
Minc_34_5     AGCATCACCAACTCCACCAAGCCACAACCAACCAAAACAGCCAAGTCCTACTCCACCAAGCTCAGCTCCTTCTCCACCAGG
Minc_34_7     AGCATCACCAACTCCACCAAGCCACAACCAACCAAAACAGCCAAGTCCTACTCCACCAAGCTCAGCTCCTTCTCCACCAGG
Minc_34_9     AGCATCACCAACTCCACCAAGCCACAACCAACCAAAACAGCCAAGTCCTACTCCACCAAGCTCAGCTCCTTCTCCACCAGG
Minc_37_10    AGCATCACCAACTCCACCAAGCCACAACCAACCAAAACAGCCAAGTCCTACTCCACCAAGCTCAGCTCCTTCTCCACCAGG
Minc_37_12    AGCATCACCAACTCCACCAAGCCACAACCAACCAAAACAGCCAAGTCCTACTCCACCAAGCTCAGCTCCTTCTCCACCAGG
Minc_37_2     AGCATCACCAACTCCACCAAGCCACAACCAACCAAAACAGCCAAGTCCTACTCCACCAAGCTCAGCTCCTTCTCCACCAGG
Minc_37_3     AGCATCACCAACTCCACCAAGCCACAACCAACCAAAACAGCCAAGTCCTACTCCACCAAGCTCAGCTCCTTCTCCACCAGG
Minc_37_4     AGCATCACCAACTCCACCAAGCCACAACCAACCAAAACAGCCAAGTCCTACTCCACCAAGCTCAGCTCCTTCTCCACCAGG
Minc_37_5     AGCATCACCAACTCCACCAAGCCACAACCAACCAAAACAGCCAAGTCCTACTCCACCAAGCTCAGCTCCTTCTCCACCAGG
Minc_37_6     AGCATCACCAACTCCACCAAGCCACAACCAACCAAAACAGCCAAGTCCTACTCCACCAAGCTCAGCTCCTTCTCCACCAGG
Minc_37_8     AGCATCACCAACTCCACCAAGCCACAACCAACCAAAACAGCCAAGTCCTACTCCACCAAGCTCAGCTCCTTCTCCACCAGG
Minc_37_9     AGCATCACCAACTCCACCAAGCCACAACCAACCAAAACAGCCAAGTCCTACTCCACCAAGCTCAGCTCCTTCTCCACCAGG
Minc_42_1     AGCATCACCAACTCCACCAAGCCACAACCAACCAAAACAGCCAAGTCCTACTCCACCAAGCTCAGCTCCTTCTCCACCAGG
```

Minc_42_10 AGCATCACCAACTCCACCAAGCCACAACCAACCAAAACAGCCAAGTCCTACTCCACCAAGCTCAGCTCCTTCTCCACCAGG
Minc_42_11 AGCATCACCAACTCCACCAAGCCACAACCAACCAAAACAGCCAAGTCCTACTCCACCAAGCTCAGCTCCTTCTCCACCAGG
Minc_42_12 AGCATCACCAACTCCACCAAGCCACAACCAACCAAAACAGCCAAGTCCTACTCCACCAAGCTCAGCTCCTTCTCCACCAGG
Minc_42_3 AGCATCACCAACTCCACCAAGCCACAACCAACCAAAACAGCCAAGTCCTACTCCACCAAGCTCAGCTCCTTCTCCACCAGG
Minc_42_4 AGCATCACCAACTCCACCAAGCCACAACCAACCAAAACAGCCAAGTCCTACTCCACCAAGCTCAGCTCCTTCTCCACCAGG
Minc_42_5 AGCATCACCAACTCCACCAAGCCACAACCAACCAAAACAGCCAAGTCCTACTCCACCAAGCTCAGCTCCTTCTCCACCAGG
Minc_42_7 AGCATCACCAACTCCACCAAGCCACAACCAACCAAAACAGCCAAGTCCTACTCCACCAAGCTCAGCTCCTTCTCCACCAGG
Minc_42_8 AGCATCACCAACTCCACCAAGCCACAACCAACCAAAACAGCCAAGTCCTACTCCACCAAGCTCAGCTCCTTCTCCACCAGG
Minc_42_9 AGCATCACCAACTCCACCAAGCCACAACCAACCAAAACAGCCAAGTCCTACTCCACCAAGCTCAGCTCCTTCTCCACCAGG
Minc_45_10 AGCATCACCAACTCCACCAAGCCACAACCAACCAAAACAGCCAAGTCCTACTCCACCAAGCTCAGCTCCTTCTCCACCAGG
Minc_45_11 AGCATCACCAACTCCACCAAGCCACAACCAACCAAAACAGCCAAGTCCTACTCCACCAAGCTCAGCTCCTTCTCCACCAGG
Minc_45_12 AGCATCACCAACTCCACCAAGCCACAACCAACCAAAACAGCCAAGTCCTACTCCACCAAGCTCAGCTCCTTCTCCACCAGG
Minc_45_13 AGCATCACCAACTCCACCAAGCCACAACCAACCAAAACAGCCAAGTCCTACTCCACCAAGCTCAGCTCCTTCTCCACCAGG
Minc_45_7 AGCATCACCAACTCCACCAAGCCACAACCAACCAAAACAGCCAAGTCCTACTCCACCAAGCTCAGCTCCTTCTCCACCAGG
Minc_45_9 AGCATCACCAACTCCACCAAGCCACAACCAACCAAAACAGCCAAGTCCTACTCCACCAAGCTCAGCTCCTTCTCCACCAGG
Minc_47_1 AGCATCACCAACTCCACCAAGCCACAACCAACCAAAACAGCCAAGTCCTACTCCACCAAGCTCAGCTCCTTCTCCACCAGG
Minc_47_10 AGCATCACCAACTCCACCAAGCCACAACCAACCAAAACAGCCAAGTCCTACTCCACCAAGCTCAGCTCCTTCTCCACCAGG
Minc_47_12 AGCATCACCAACTCCACCAAGCCACAACCAACCAAAACAGCCAAGTCCTACTCCACCAAGCTCAGCTCCTTCTCCACCAGG
Minc_47_13 AGCATCACCAACTCCACCAAGCCACAACCAACCAAAACAGCCAAGTCCTACTCCACCAAGCTCAGCTCCTTCTCCACCAGG
Minc_47_3 AGCATCACCAACTCCACCAAGCCACAACCAACCAAAACAGCCAAGTCCTACTCCACCAAACTCAGCTCCTTCTCCACCAGG
Minc_47_7 AGCATCACCAACTCCACCAAGCCACAACCAACCAAAACAGCCAAGTCCTACTCCACCAAGCTCAGCTCCTTCTCCACCAGG
Minc_47_8 AGCATCACCAACTCCACCAAGCCACAACCAACCAAAACAGCCAAGTCCTACTCCACCAAGCTCAGCTCCTTCTCCACCAGG
Minc_51_10 AGCATCACCAACTCCACCAAGCCACAACCAACCAAAACAGCCAAGTCCTACTCCACCAAGCTCAGCTCCTTCTCCACCAGG
Minc_51_11 AGCATCACCAACTCCACCAAGCCACAACCAACCAAAACAGCCAAGTCCTACTCCACCAAACTCAGCTCCTTCTCCACCAGG
Minc_51_4 AGCATCACCAACTCCACCAAGCCACAACCAACCAAAACAGCCAAGTCCTACTCCACCAAACTCAGCTCCTTCTCCACCAGG
Minc_51_6 AGCATCACCAACTCCACCAAGCCACAACCAACCAAAACAGCCAAGTCCTACTCCACCAAGCTCAGCTCCTTCTCCACCAGG
Minc_51_7 AGCATCACCAACTCCACCAAGCCACAACCAACCAAAACAGCCAAGTCCTACTCCACCAAGCTCAGCTCCTTCTCCACCAGG
Minc_54_1 AGCATCACCAACTCCACCAAGCCACAACCAACCAAAACAGCCAAGTCCTACTCCACCAAGCTCAGCTCCTTCTCCACCAGG
Minc_54_10 AGCATCACCAACTCCACCAAGCCACAACCAACCAAAACAGCCAAGTCCTACTCCACCAAGCTCAGCTCCTTCTCCACCAGG
Minc_54_3 AGCATCACCAACTCCACCAAGCCACAACCAACCAAAACAGCCAAGTCCTACTCCACCAAGCTCAGCTCCTTCTCCACCAGG
Minc_54_4 AGCATCACCAACTCCACCAAGCCACAACCAACCAAAACAGCCAAGTCCTACTCCACCAAGCTCAGCTCCTTCTCCACCAGG
Minc_54_5 AGCATCACCAACTCCACCAAGCCACAACCAACCAAAACAGCCAAGTCCTACTCCACCAAGCTCAGCTCCTTCTCCACCAGG
Minc_54_6 AGCATCACCAACTCCACCAAGCCACAACCAACCAAAACAGCCAAGTCCTACTCCACCAAGCTCAGCTCCTTCTCCACCAGG
Minc_56_1 AGCATCACCAACTCCACCAAGCCACAACCAACCAAAACAGCCAAGTCCTACTCCACCAAGCTCAGCTCCTTCTCCACCAGG
Minc_56_12 AGCATCACCAACTCCACCAAGCCACAACCAACCAAAACAGCCAAGTCCTACTCCACCAAGCTCAGCTCCTTCTCCACCAGG
Minc_56_2 AGCATCACCAACTCCACCAAGCCACAACCAACCAAAACAGCCAAGTCCTACTCCACCAAGCTCAGCTCCTTCTCCACCAGG
Minc_56_3 AGCATCACCAACTCCACCAAGCCACAACCAACCAAAACAGCCAAGTCCTACTCCACCAAGCTCAGCTCCTTCTCCACCAGG
Minc_56_4 AGCATCACCAACTCCACCAAGCCACAACCAACCAAAACAGCCAAGTCCTACTCCACCAAG-TCAGCTCCTTCTCCACCAGG
Minc_56_5 AGCATCACCAACTCCACCAAGCCACAACCAACCAAAACAGCCAAGTCCTACTCCACCAAG-TCAGGTCCTTCTCCACCAGG
Minc_56_6 AGCATCACCAACTCCACCAAGCCACAACCAACCAAAACAGCCAAGTCCTACTCCACCAAG-TCAGCTCCTTCTCCACCAGG
Minc_56_7 AGCATCACCAACTCCACCAAGCCACAACCAACCAAAACAGCCAAGTCCTACTCCACCAAG-TCAGCTCCTTCTCCACCAGG
Minc_58_1 AGCATCACCAACTCCACCAAGCCACAACCAACCAAAACAGCCAAGTCCTACTCCACCAAGCTCAGCTCCTTCTCCACCAGG
Minc_58_13 AGCATCACCAACTCCACCAAGCCACAACCAACCAAAACAGCCAAGTCCTACTCCACCAAGCTCAGCTCCTTCTCCACCAGG
Minc_58_2 AGCATCACCAACTCCACCAAGCCACAACCAACCAAAACAGCCAAGTCCTACTCCACCAAGCTCAGCTCCTTCTCCACCAGG
Minc_58_3 AGCATCACCAACTCCACCAAGCCACAACCAACCAAAACAGCCAAGTCCTACTCCACCAAGCTCAGCTCCTTCTCCACCAGG
Minc_58_4 AGCATCACCAACTCCACCAAGCCACAACCAACCAAAACAGCCAAGTCCTACTCCACCAAGCTCAGCTCCTTCTCCACCAGG
Minc_58_5 AGCATCACCAACTCCACCAAGCCACAACCAACCAAAACAGCCAAGTCCTACTCCACCAAGCTCAGCTCCTTCTCCACCAGG
Minc_58_6 AGCATCACCAACTCCACCAAGCCACAACCAACCAAAACAGCCAAGTCCTACTCCACCAAGCTCAGCTCCTTCTCCACCAGG
Minc_58_7 AGCATCACCAACTCCACCAAGCCACAACCAACCAAAACAGCCAAGTCCTACTCCACCAAGCTCAGCTCCTTCTCCACCAGG
Minc_58_8 AGCATCACCAACTCCACCAAGCCACAACCAACCAAAACAGCCAAGTCCTACTCCACCAAGCTCAGCTCCTTCTCCACCAGG
Minc_58_9 AGCATCACCAACTCCACCAAGCCACAACCAACCAAAACAGCCAAGTCCTACTCCACCAAGCTCAGCTCCTTCTCCACCAGG
Minc_65_1 AGCATCACCAACTCCACCAAGCCACAACCAACCAAAACAGCCAAGTCCTACTCCACCAAGCTCAGCTCCTTCTCCACCAGG
Minc_65_11 AGCATCACCAACTCCACCAAGCCACAACCAACCAAAACAGCCAAGTCCTACTCCACCAAGCTCAGCTCCTTCTCCACCAGG
Minc_65_3 AGCATCACCAACTCCACCAAGCCACAACCAACCAAAACAGCCAAGTCCTACTCCACCAAGCTCAGCTCCTTCTCCACCAAG
Minc_65_6 AGCATCACCAACTCCACCAAGCCACAACCAACCAAAACAGCCAAGTCCTACTCCACCAAGCTCAGCTCCTTCTCCACCAGG
Minc_65_8 AGCATCACCAACTCCACCAAGCCACAACCAACCAAAACAGCCAAGTCCTACTCCACCAAGCTCAGCTCCTTCTCCACCAGG
Minc_65_9 AGCATCACCAACTCCACCAAGCCACAACCAACCAAAACAGCCAAGTCCTACTCCACCAAGCTCAGCTCCTTCTCCACCAGG
Minc_76_1 AGCATCACCAACTCCACCAAGCCACAACCAACCAAAACAGCCAAGTCCTACTCCACCAAGCTCAGCTCCTTCTCCACCAGG
Minc_76_10 AGCATCACCAACTCCACCAAGCCACAACCAACCAAAACAGCCAAGTCCTACTCCACCAAGCTCAGCTCCTTCTCCACCAGG
Minc_76_12 AGCATCACCAACTCCACCAAGCCACAACCAACCAAAACAGCCAAGTCCTACTCCACCAAGCTCAGCTCCTTCTCCACCAGG
Minc_76_13 AGCATCACCAACTCCACCAAGCCACAACCAACCAAAACAGCCAAGTCCTACTCCACCAAGCTCAGCTCCTTCTCCACCAGG
Minc_76_14 AGCATCACCAACTCCACCAAGCCACAACCAACCAAAACAGCCAAGTCCTACTCCACCAAGCTCAGCTCCTTCTCCACCAGG
Minc_76_3 AGCATCACCAACTCCACCAAGCCACAACCAACCAAAACAGCCAAGTCCTACTCCACCAAGCTCAGCTCCTTCTCCACCAGG
Minc_76_7 AGCATCACCAACTCCACCAAGCCACAACCAACCAAAACAGCCAAGTCCTACTCCACCAAGCTCAGCTCCTTCTCCACCAGG
Minc_76_8 AGCATCACCAACTCCACCAAGCCACAACCAACCAAAACAGCCAAGTCCTACTCCACCAAGCTCAGCTCCTTCTCCACCAGG
Minc_76_9 AGCATCACCAACTCCACCAAGCCACAACCAACCAAAACAGCCAAGTCCTACTCCACCAAGCTCAGCTCCTTCTCCACCAGG
Minc_78_10 AGCATCACCAACTCCACCAAGCCACAACCAACCAAAACAGCCAAGTCCTACTCCACCAAGCTCAGCTCCTTCTCCACCAGG
Minc_78_11 AGCATCACCAACTCCACCAAGCCACAACCAACCAAAACAGCCAAGTCCTACTCCACCAAGCTCAGCTCCTTCTCCACCAGG
Minc_78_13 AGCATCACCAACTCCACCAAGCCACAACCAACCAAAACAGCCAAGTCCTACTCCACCAAGCTCAGCTCCTTCTCCACCAGG
Minc_78_14 AGCATCACCAACTCCACCAAGCCACAACCAACCAAAACAGCCAAGTCCTACTCCACCAAGCTCAGCTCCTTCTCCACCAGG
Minc_78_15 AGCATCACCAACTCCACCAAGCCACAACCAACCAAAACAGCCAAGTCCTACTCCACCAAGCTCAGCTCCTTCTCCACCAGG
Minc_78_16 AGCATCACCAACTCCACCAAGCCACAACCAACCAAAACAGCCAAGTCCTACTCCACCAAGCTCAGCTCCTTCTCCACCAGG
Minc_78_17 AGCATCACCAACTCCACCAAGCCACAACCAACCAAAACAGCCAAGTCCTACTCCACCAAGCTCAGCTCCTTCTCCACCAGG
Minc_78_7 AGCATCACCAACTCCACCAAGCCACAACCAACCAAAACAGCCAAGTCCTACTCCACCAAGCTCAGCTCCTTCTCCACCAGG
Minc_81_12 AGCATCACCAACTCCACCAAGCCACAACCAACCAAAACAGCCAAGTCCTACTCCACCAAGCTCAGCTCCTTCTCCACCAGG
Minc_81_13 AGCATCACCAACTCCACCAAGCCACAACCAACCAAAACAGCCAAGTCCTACTCCACCAAGCTCAGCTCCTTCTCCACCAGG
Minc_81_14 AGCATCACCAACTCCACCAAGCCACAACCAACCAAAACAGCCAAGTCCTACTCCACCAAGCTCAGCTCCTTCTCCACCAGG
Minc_81_15 AGCATCACCAACTCCACCAAGCCACAACCAACCAAAACAGCCAAGTCCTACTCCACCAAGCTCAGCTCCTTCTCCACCAGG
Minc_81_2 AGCATCACCAACTCCACCAAGCCACAACCAACCAAAACAGCCAAGTCCTACTCCACCAAGCTCAGCTCCTTCTCCACCAGG
Minc_81_4 AGCATCACCAACTCCACCAAGCCACAACCAACCAAAACAGCCAAGTCCTACTCCACCAAGCTCAGCTCCTTCTCCACCAGG
Minc_81_6 AGCATCACCAACTCCACCAAGCCACAACCAACCAAAACAGCCAAGTCCTACTCCACCAAGCTCAGCTCCTTCTCCACCAGG
Minc_81_8 AGCATCACCAACTCCACCAAGCCACAACCAACCAAAACAGCCAAGTCCTACTCCACCAAGCTCAGCTCCTTCTCCACCAGG
** **** ************ *
