# Supplementary material for: The map-1 Gene Family in Root-Knot Nematodes, Meloidogyne spp.: A Set of Taxonomically Restricted Genes Specific to Clonal Species
Source: PLoS One. 2012 Jun 18;7(6):e38656. doi: 10.1371/journal.pone.0038656 (PMC3377709; doi:10.1371/journal.pone.0038656)
Supplement: Figure S2 — Distribution and structural organisation of map-1 genes in root-knot nematodes. The orange and blue boxes correspond to regions encoding the 58-aa and 13-aa repeats in MAP-1, respectively. The blue triangles indicate regions encoding the 13-aa truncated repeats. (PDF) [file pone.0038656.s002.pdf]

Figure S2. Distribution and structural organisation of *map-1* genes in root-knot nematodes. The orange and blue boxes correspond to regions encoding the 58-aa and 13-aa repeats in MAP-1, respectively. The blue triangles indicate regions encoding the 13-aa truncated repeats.

***map-1* repeats**	**species**	**name of the genes**[a]	**references**
**1) with complete last short repeat:**			
	M. incognita	*map-1.1*	[13]
	M. javanica	*mj-map-1.1*	[22]
	M. arabicida, M. arenaria, M. cruciani, M. enterolobii, M. ethiopica, M. floridensis, M. hispanica, M. inornata, M. izalcoensis, M. konaensis, M. paranaensis		this study
	M. hispanica, M. izalcoensis, M. konaensis, M. paranaensis	-	this study
	M. paranaensis	-	this study
**2) with truncated last short repeat:**			
	M. javanica	*mj-map-1.2*	[22]
	M. arenaria, M. cruciani, M. izalcoensis		this study
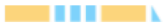	*M. izalcoensis*	-	this study
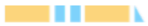	*M. incognita*	*map-1.2*	[13]
	M. ethiopica, M. inornata		this study
	M. incognita	*map-1.3*	[13]
	M. izalcoensis		this study

[a]Here are given the names of the genes already described in literature.
