# Supplementary material for: The map-1 Gene Family in Root-Knot Nematodes, Meloidogyne spp.: A Set of Taxonomically Restricted Genes Specific to Clonal Species
Source: PLoS One. 2012 Jun 18;7(6):e38656. doi: 10.1371/journal.pone.0038656 (PMC3377709; doi:10.1371/journal.pone.0038656)
Supplement: Table S1 — List of the nematode-specific nucleotide resources used in BlastN analysis. Grey shading identifies the species in which map-1 homologs were found. (PDF) [file pone.0038656.s003.pdf]

Table S1. List of the nematode-specific nucleotide resources used in BlastN analysis. Stars identify the species in which *map-1* homologs were found.

Database	Sequences searched	Species	Clade[a]
WormBase	Completed genomic sequences	*Brugia malayi*	III
		Caenorhabditis angaria	V
		Caenorhabditis brenneri	V
		Caenorhabditis briggsae	V
		Caenorhabditis elegans	V
		Caenorhabditis japonica	V
		Caenorhabditis elegans	V
		Haemonchus contortus	V
		*Meloidogyne incognita**	IV
		Meloidogyne hapla	IV
		Pristionchus pacificus	V
Nematode.net	EST clusters	*Ancylostoma caninum*	V
		Ancylostoma ceylanicum	V
		Ascaris suum	III
		Brugia malayi	III
		Caenorhabditis elegans	V
		Caenorhabditis remanei	V
		Cooperia oncophora	V
		Diriofilaria immitis	III
		Ditylenchus africanus	IV
		Dictyocaulus viviparus	IV
		Globodera pallida	IV
		Globodera rostochiensis	IV
		Haemonchus contortus	V
		Heterodera glycines	IV
		Heterodera schachtii	IV
		Heterorhabditis bacteriophora	V
		Laxus oneistus	IV
		*Meloidogyne arenaria**	IV
		Meloidogyne chitwoodi	IV
		Meloidogyne hapla	IV
		*Meloidogyne incognita**	IV
		*Meloidogyne javanica**	IV
		Meloidogyne paranaensis	IV
		Necator americanus	V
		Nippostrongylus brasiliensis	V
		Oesophagostomum dentatum	V
		Onchocerca flexuosa	III
		Onchocerca volvulus	III
		Ostertagia ostertagi	V
		Parastrongyloides trichosuri	IV
		Pratylenchus penetrans	IV
		Pristionchus pacificus	V
		Radopholus similis	IV
		Strongyloides ratti	IV
		Strongyloides stercoralis	IV
		Teladorsagia circumcincta	V
		Toxocara canis	III
		Trichinella spiralis	I
		Trichostrongylus colubriformis	V
		Trichuris muris	I
		Trichuris vulpis	I
		Xiphinema index	I
		Zeldia punctata	IV

[a]Clades were denominated according to [53]
