# Supplementary material for: The map-1 Gene Family in Root-Knot Nematodes, Meloidogyne spp.: A Set of Taxonomically Restricted Genes Specific to Clonal Species
Source: PLoS One. 2012 Jun 18;7(6):e38656. doi: 10.1371/journal.pone.0038656 (PMC3377709; doi:10.1371/journal.pone.0038656)
Supplement: Table S2 — List of the rDNA SSU sequences used to infer phylogenetic relationships of root-knot nematodes. (DOCX) [file pone.0038656.s004.docx]

**Table S2.** List of the rDNA SSU sequences used to infer phylogenetic

relationships of root-knot nematodes.

| **Species** | **Accession** | **Reference** |
| --- | --- | --- |
| *Meloidogyne arabicida* | HE667738 | this study |
| *Meloidogyne arenaria* | U42342 | [56] |
| *Meloidogyne artiellia* | AF248477 | [57] |
| *Meloidogyne chitwoodi* | AY593883 | [21] |
| *Meloidogyne coffeicola* | HE667739 | this study |
| *Meloidogyne cruciani* | HE667740 | this study |
| *Meloidogyne enterolobii* | AY942629 | [19] |
| *Meloidogyne ethiopica* | AY942630 | [19] |
| *Meloidogyne exigua* | AY942627 | [19] |
| *Meloidogyne fallax* | AY593895 | [21] |
| *Meloidogyne floridensis* | AY942621 | [19] |
| *Meloidogyne hapla* | AY593892 | [21] |
| *Meloidogyne hispanica* | HE667741 | this study |
| *Meloidogyne ichinohei* | EU669953 | [20] |
| *Meloidogyne incognita* | AY284621 | [42] |
| *Meloidogyne inornata* | HE667742 | this study |
| *Meloidogyne izalcoensis* | HE667743 | this study |
| *Meloidogyne javanica* | AY268121 | Lee & Williamson, unpublished |
| *Meloidogyne konaensis* | HE667744 | this study |
| *Meloidogyne naasi* | AY593900 | [21] |
| *Meloidogyne paranaensis* | AY942622 | [19] |

1. Aleshin VV, Kedrova OS, Milyutina IA, Vladychenskaya NS, Petrov NB (1998) Secondary structure of some elements of 18S rRNA suggests that strongylid and a part of rhabditid nematodes are monophyletic. FEBS Lett 429:4-8.
2. De Giorgi C , Veronico P , De Luca F, Natilla A, Lanave C, et al. (2002) Structural and evolutionary analysis of the ribosomal genes of the parasitic nematode *Meloidogyne artiellia* suggests its ancient origin. Mol. Biochem. Parasitol. 124: 91-94.
