# Supplementary material for: The map-1 Gene Family in Root-Knot Nematodes, Meloidogyne spp.: A Set of Taxonomically Restricted Genes Specific to Clonal Species
Source: PLoS One. 2012 Jun 18;7(6):e38656. doi: 10.1371/journal.pone.0038656 (PMC3377709; doi:10.1371/journal.pone.0038656)
Supplement: Table S3 — List of the Meloidogyne incognita isolates used in this study, with their geographic origin and virulence status against the tomato Mi-1 resistance gene. (PDF) [file pone.0038656.s005.pdf]

Table S3. List of the *Meloidogyne incognita* isolates used in this study, with their geographic origin and virulence status against the tomato *Mi-1* resistance gene.

Code	Geographic origin	Virulence	No. samples[a]
Minc_18	Wageningen, The Netherlands	virulent	10
Minc_22	Morelos, Mexico	avirulent	7
Minc_30	Calissane, France	virulent[b]	5
Minc_34	Valbonne, France	virulent	8
Minc_37	Antibes, France	avirulent	9
Minc_42	Nanjing, China	avirulent	10
Minc_45	Morelos, Mexico	virulent[b]	6
Minc_47	N'Gorom, Senegal	virulent	7
Minc_51	Calissane, France	avirulent	5
Minc_54	Kursk, Russia	virulent[b]	6
Minc_56	Ica, Peru	avirulent	8
Minc_58	Turkey	virulent	10
Minc_65	Londrina, Brazil	avirulent	6
Minc_76	Sevilla, Spain	avirulent	9
Minc_78	?	virulent	8
Minc_81	Adiopodoumé, Ivory Coast	virulent	8

[a]number of sequences amplified from individual nematodes used to study the infraspecific polymorphism of *map-1.1*

[b]isolates selected for virulence in the laboratory
